# A High-Resolution Anatomical Atlas of the Transcriptome in the Mouse Embryo

**DOI:** 10.1371/journal.pbio.1000582

**Published:** 2011-01-18

**Authors:** Graciana Diez-Roux, Sandro Banfi, Marc Sultan, Lars Geffers, Santosh Anand, David Rozado, Alon Magen, Elena Canidio, Massimiliano Pagani, Ivana Peluso, Nathalie Lin-Marq, Muriel Koch, Marchesa Bilio, Immacolata Cantiello, Roberta Verde, Cristian De Masi, Salvatore A. Bianchi, Juliette Cicchini, Elodie Perroud, Shprese Mehmeti, Emilie Dagand, Sabine Schrinner, Asja Nürnberger, Katja Schmidt, Katja Metz, Christina Zwingmann, Norbert Brieske, Cindy Springer, Ana Martinez Hernandez, Sarah Herzog, Frauke Grabbe, Cornelia Sieverding, Barbara Fischer, Kathrin Schrader, Maren Brockmeyer, Sarah Dettmer, Christin Helbig, Violaine Alunni, Marie-Annick Battaini, Carole Mura, Charlotte N. Henrichsen, Raquel Garcia-Lopez, Diego Echevarria, Eduardo Puelles, Elena Garcia-Calero, Stefan Kruse, Markus Uhr, Christine Kauck, Guangjie Feng, Nestor Milyaev, Chuang Kee Ong, Lalit Kumar, MeiSze Lam, Colin A. Semple, Attila Gyenesei, Stefan Mundlos, Uwe Radelof, Hans Lehrach, Paolo Sarmientos, Alexandre Reymond, Duncan R. Davidson, Pascal Dollé, Stylianos E. Antonarakis, Marie-Laure Yaspo, Salvador Martinez, Richard A. Baldock, Gregor Eichele, Andrea Ballabio

**Affiliations:** 1 Telethon Institute of Genetics and Medicine, Naples, Italy; 2 Max Planck Institute for Molecular Genetics, Berlin, Germany; 3 Genes and Behavior Department, Max Planck Institute of Biophysical Chemistry, Goettingen, Germany; 4 Primm, Milan, Italy; 5 Department of Genetic Medicine and Development, University of Geneva Medical School, Geneva, Switzerland; 6 Institut Clinique de la Souris, Illkirch, France; 7 Center for Integrative Genomics, University of Lausanne, Lausanne, Switzerland; 8 Experimental Embryology Lab, Instituto de Neurociencias, Universidad Miguel Hernandez, San Juan de Alicante, Spain; 9 ORGARAT, Essen, Germany; 10 Medical Research Council Human Genetics Unit, Western General Hospital, Edinburgh, United Kingdom; 11 RZPD—Deutsches Ressourcenzentrum für Genomforschung, Berlin, Germany; 12 Institut de Génétique et de Biologie Moléculaire et Cellulaire, Inserm U 964, CNRS UMR 7104, Faculté de Médecine, Université de Strasbourg; Illkirch, France; 13 University Hospitals of Geneva, Geneva, Switzerland; 14 Medical Genetics, Department of Pediatrics, Federico II University, Naples, Italy; 15 Department of Molecular and Human Genetics, Baylor College of Medicine, Houston, Texas, United States of America; 16 Jan and Dan Duncan Neurological Research Institute, Texas Children's Hospital, Houston, Texas, United States of America; Stanford University, United States of America

## Abstract

Ascertaining when and where genes are expressed is of crucial importance to understanding or predicting the physiological role of genes and proteins and how they interact to form the complex networks that underlie organ development and function. It is, therefore, crucial to determine on a genome-wide level, the spatio-temporal gene expression profiles at cellular resolution. This information is provided by colorimetric RNA in situ hybridization that can elucidate expression of genes in their native context and does so at cellular resolution. We generated what is to our knowledge the first genome-wide transcriptome atlas by RNA in situ hybridization of an entire mammalian organism, the developing mouse at embryonic day 14.5. This digital transcriptome atlas, the Eurexpress atlas (http://www.eurexpress.org), consists of a searchable database of annotated images that can be interactively viewed. We generated anatomy-based expression profiles for over 18,000 coding genes and over 400 microRNAs. We identified 1,002 tissue-specific genes that are a source of novel tissue-specific markers for 37 different anatomical structures. The quality and the resolution of the data revealed novel molecular domains for several developing structures, such as the telencephalon, a novel organization for the hypothalamus, and insight on the Wnt network involved in renal epithelial differentiation during kidney development. The digital transcriptome atlas is a powerful resource to determine co-expression of genes, to identify cell populations and lineages, and to identify functional associations between genes relevant to development and disease.

## Abstract 2

The manuscript describes the “digital transcriptome atlas” of the developing mouse embryo, a powerful resource to determine co-expression of genes, to identify cell populations and lineages and to identify functional associations between genes relevant to development and disease.

## Author Summary

In situ hybridization (ISH) can be used to visualize gene expression in cells and tissues in their native context. High-throughput ISH using nonradioactive RNA probes allowed the Eurexpress consortium to generate a comprehensive, interactive, and freely accessible digital gene expression atlas, the Eurexpress transcriptome atlas (http://www.eurexpress.org), of the E14.5 mouse embryo. Expression data for over 15,000 genes were annotated for hundreds of anatomical structures, thus allowing us to systematically identify tissue-specific and tissue-overlapping gene networks. We illustrate the value of the Eurexpress atlas by finding novel regional subdivisions in the developing brain. We also use the transcriptome atlas to allocate specific components of the complex Wnt signaling pathway to kidney development, and we identify regionally expressed genes in liver that may be markers of hematopoietic stem cell differentiation.

## Introduction

Genomic research has significantly advanced our understanding of physiological and pathophysiological processes, ranging from infectious diseases to cancer. Two fundamental aspects of this approach are the generation of large datasets and the systematic integration of the information contained therein. Transcriptome analysis has been in the forefront of this research field. Ascertaining when and where genes are expressed is of crucial importance to understanding or predicting the physiological role of genes and proteins and how they interact to form the complex networks that underlie organ development and function. Progress in understanding gene networks is driven by massive parallel approaches [1]–[4] that capture the complexity of a gene network as a whole. However, genome-scale approaches capable of unraveling events occurring in single cells or small groups of cells still pose a major challenge. In recent years, high-throughput methods that collect such information at cellular resolution on a gene-by-gene basis have been developed. Of particular relevance was the development of high-throughput technology for RNA in situ hybridization (ISH) to map gene expression patterns on tissue sections [5]–[7]. A widely used resource based on this technology is the Allen Brain Atlas (ABA) [8], a digital genome-wide atlas of gene expression in the adult mouse brain. Additional valuable resources documenting organ-specific gene expression using similar approaches include the Gene Expression Nervous System Altas (GENSAT), the GenitoUrinary Development Molecular Anatomy Project (GUDMAP), and the St. Jude Brain Gene Expression Map (BGEM) [9]–[11]. Efforts to integrate expression data that bring together information from diverse sources are the Edinburgh Mouse Atlas of Gene Expression (EMAGE) [12] and the Mouse Genome Informatics (MGI) Gene Expression Database (GXD) [13]. These databases use published gene expression data descriptions to provide expression annotations that follow standard anatomy ontology. The next challenge, partially addressed in *Drosophila melanogaster* [14],[15], is the generation of a transcriptome map of an entire organism at cellular resolution.

Here we report the generation of the Eurexpress transcriptome atlas, which delivers the expression patterns of almost all *Mus musculus* protein-coding genes (more than 18,000 genes) in the developing mouse at embryonic day 14.5 (E14.5) by RNA ISH. These data were organized and annotated to build a Web-based gene expression atlas freely available to the scientific community (http://www.eurexpress.org). This atlas is to our knowledge the first resource generated in a mammalian organism that provides a simultaneous visualization of thoroughly annotated gene expression patterns at cellular resolution at one developmental stage.

## Results

### The Transcriptome Atlas

We analyzed the expression patterns of over 18,000 transcripts (18,264), mostly corresponding to protein-coding genes, by RNA ISH in the developing wild-type laboratory mouse. The colorimetric ISH was performed on frozen sagittal sections of C57BL/6J wild-type mice at E14.5. At this developmental stage, organogenesis is largely complete, making it an adequate model to study organ architecture and function, and, in addition, stem cell division and cell differentiation are still ongoing. Each gene was analyzed on a set of 24 sagittal sections, which all together provide a complete representation of all embryonic tissues [5]. We set up semi-automated pipelines to design one appropriate probe per gene (Figure S1), with the aim of capturing most of the isoforms generated by alternative splicing. We also included a set of locked nucleic acid (LNA) probes covering the mature sequences of 444 murine microRNAs in the analysis.

**Figure 1:**
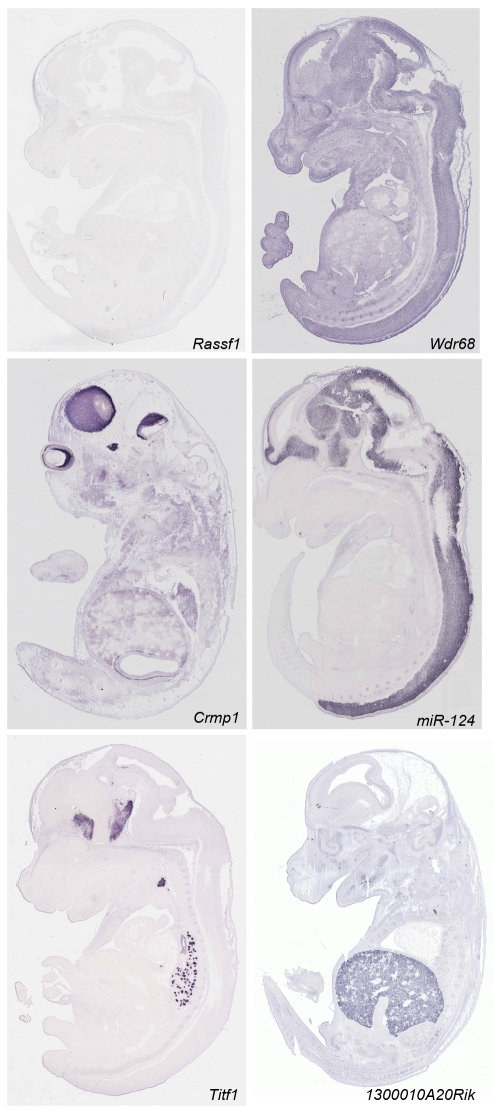
Representative examples of RNA ISH data of E14.5 embryos. The expression categories defined by the annotation summary are illustrated by the following examples. (1) Expression not detected: *Rassf1* messenger RNA is not detected at this stage. (2) Homogeneous (non-regional) signal: *Wdr68* shows hybridization signal in all tissues and structures. (3) Regionally expressed genes: *Crmp1*, *Mir124*, *Titf1*, and *1300010A20Rik*. *Crmp1* signal is evident in the brain, the V trigeminal ganglion, the spinal cord, and the neural retina. *miR124* is restricted to the nervous system. *Titf1* expression is detected in the diencephalon, hypothalamus, telencephalon, thyroid, and lung. *1300010A20Rik* is an example of a tissue-specific gene with expression limited to the liver. Complete sets of images for 19,411 genes are available at http://www.eurexpress.org.

After ISH and automated microscopy image acquisition [16], expression patterns were manually annotated by expert anatomists using a revised version of the Edinburgh Mouse Atlas Project (EMAP) anatomy ontology, which includes 1,420 anatomical terms. The EMAP mouse anatomy ontology (http://www.emouseatlas.org/Databases/Anatomy/new/theiler23.shtml) is widely accepted and is used as the basis for annotating expression patterns in other large-scale expression resources such as EMAGE and MGI. This ontology supports annotation at different levels of resolution through automatic inheritance of properties between levels. In addition to identifying expression sites, our curated annotation provided information on the expression pattern (homogeneous, regional, or single cell) and on its strength (strong, moderate, or weak), revealing detailed patterns even for genes expressed at low levels. Compiling all ∼15,500 annotated patterns allowed classifying them into three broad categories: 39% were “regional” (signal detected in a limited number of discrete locations), 43% showed a nonregional signal in all tissues, and 18% were not detected. Figure 1 shows examples of these three categories. All images and their annotation are available and searchable at http://www.eurexpress.org.

**Figure 2:**
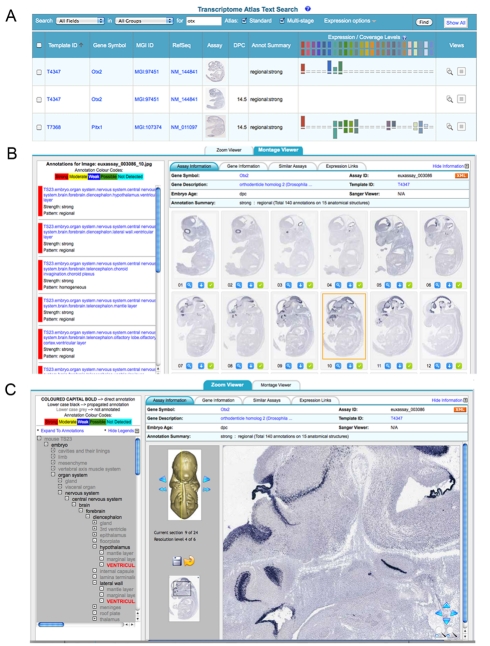
Snapshot view of the Web-based transcriptome atlas. (A) Keyword search results showing a table format including a thumbnail view of an image, and visualizing each embryonic section and associated anatomical annotation, color-coded according to expression strength. (B) Clicking on a particular image allows viewing the annotation associated with the particular image (left panel). Top tabs give additional details and links to other gene expression Web sites and genomic resources. (C) Zoom viewer. The image viewer provides full resolution images with standard zoom and pan capability. In addition, the viewed section can be selected using the 3-D embryo view. The left-hand panel shows the annotation in the context of the anatomy ontology, and the tabs provide additional detail and links to other gene expression and genomic resources.

The Eurexpress database allows basic and advanced queries by annotated anatomy, gene name, symbol, template, and gene sequence. The search interface provides both a thumbnail view of a representative section and the annotation summary (Figure 2A). The expression data can be visualized in the form of either a montage viewer (Figure 2B) or a zoom/panning viewer (virtual microscope, Figure 2C). All expression patterns are linked to expression databases, such as the ABA [8], EMAGE [12],[17], and the Gene Expression Nervous System Altas [11], and to bioinformatics resources such as Entrez Gene, ENSEMBL, and MGI. Additional features of Eurexpress include a standard anatomy reference atlas based on a set of eight sagittal histology sections that have been graphically annotated. These section views have a user-controlled overlay capability as well as the standard zoom viewer and can be used in conjunction with the assay image views to enable convenient comparison (http://www.eurexpress.org/eAtlasViewer/php/eurexpressAnatomyAtlas.php).

### Validation

A quality control study on 250 solute carrier genes (*Slc*) characterized with the same ISH protocol [18] but using probes generated by PCR amplification with specific primers revealed over 90% concordance, indicating that our template resource was reliable (see Table S1). We also compared 1,089 expression patterns (including genes with tissue-restricted expression and a subset of disease genes) to previously published data, collected at the same stage and using the same methodology, by using the literature query form of the MGI Gene Expression Database (http://www.informatics.jax.org/searches/gxdindex_form.shtml). We found data in the literature for 14% of these, and the analysis revealed 84% overall concordance between the two datasets. The comparison was done by visual inspection, and concordance/partial concordance was scored when the sites of expression were the same or overlapping in the two datasets. Table S2 includes the results and the appropriate literature references. Interestingly, if we restrict the same analysis to a subset of more characterized genes, namely, 100 disease genes, for which we found published expression data in 72% of cases, the concordance reaches 97%, giving a clear indication of the equivalence between datasets when studying well-characterized genes. Overall, these results underscore the reliability of our data as tested against published data.

We compared our expression data to those obtained from microarrays using RNA from whole E14.5 embryos [19]. This comparison revealed that 30% of the genes determined as regional by ISH could not be detected by microarray (GSE-6081) (e.g., *Titf1*; Figure 1). In addition, we also compared Eurexpress data to the results of a microarray experiment carried out using RNA from the E14.5 mouse heart (E-GEOD-1479 in the Gene Expression Omnibus database). The comparative analysis revealed that of the 397 regional genes annotated to be expressed in the heart in Eurexpress, 20% (78 genes) were not detected by the microarray experiment described above. These data underline the value of ISH for revealing the expression of genes with very specific or restricted patterns.

### Expression Analysis and Expression Clustering

**Figure 3:**
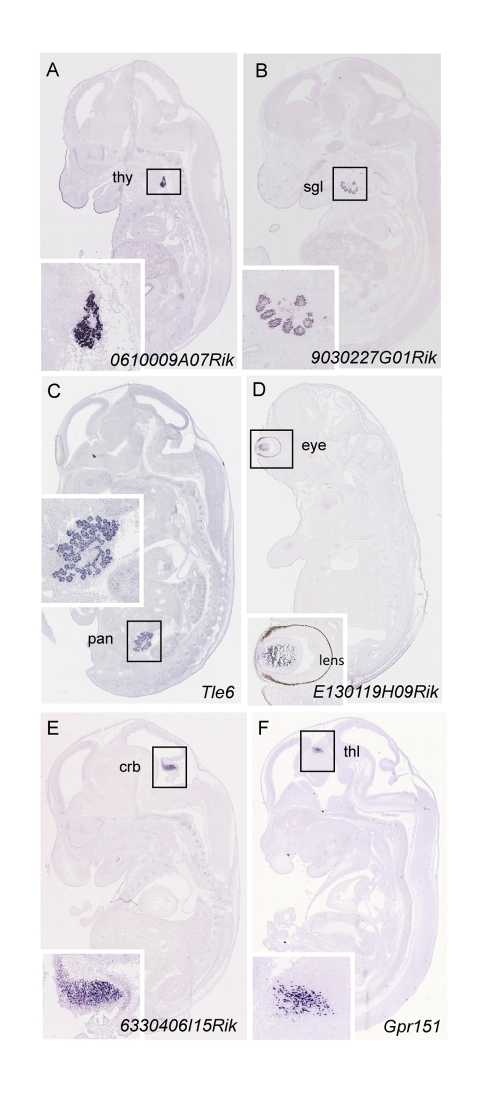
Representative examples of RNA ISH data that show gene expression patterns restricted to specific anatomical structures. (A) *0610009A07Rik* is expressed in the thyroid; (B) *9030227G01Rik* in the salivary glands; (C) *Tle6* in the pancreas; (D) *E130119H09Rik* in the eye; (E) *6330406I15Rik* in the cerebellum; and (F) *Gpr151* in the thalamus. Insets are higher magnification views of expression shown in main panels and show in greater detail the sites of expression. crb, cerebellum; pan, pancreas; sgl, salivary glands; thl, thalamus; thy, thyroid.

We performed data mining on genes annotated as regional to gain insight into the transcriptome complexity of the main organs and anatomical structures at E14.5. This analysis revealed that the tissues displaying the highest expression complexity belong to the central nervous system (CNS), accounting for 60% (*n = *3,902) of regionally expressed genes, followed by the alimentary system (45%, *n = *2,912) and the sensory organs (43%, *n = *2,730) (Figure S2). We identified approximately 1,000 genes that display exclusive expression in a specific anatomical structure (Table S3), 16% of which have unknown function. For example, we identified 106 markers for specific structures of the CNS (e.g., cerebral cortex, thalamus, hypothalamus), 218 for specific structures of the alimentary system (147 of which are exclusively expressed in the liver), and 127 for the thymus. This collection represents an extraordinary source of novel histological markers for 37 different anatomical structures (see Figure 3 for specific examples and Table S4 for a complete summary). This novel catalog of genes with restricted expression patterns constitutes an invaluable tool for the identification of sequence control elements driving gene expression in specific tissues and organs and will be useful for the design of tissue-specific mouse CRE driver lines [20].

**Figure 4:**
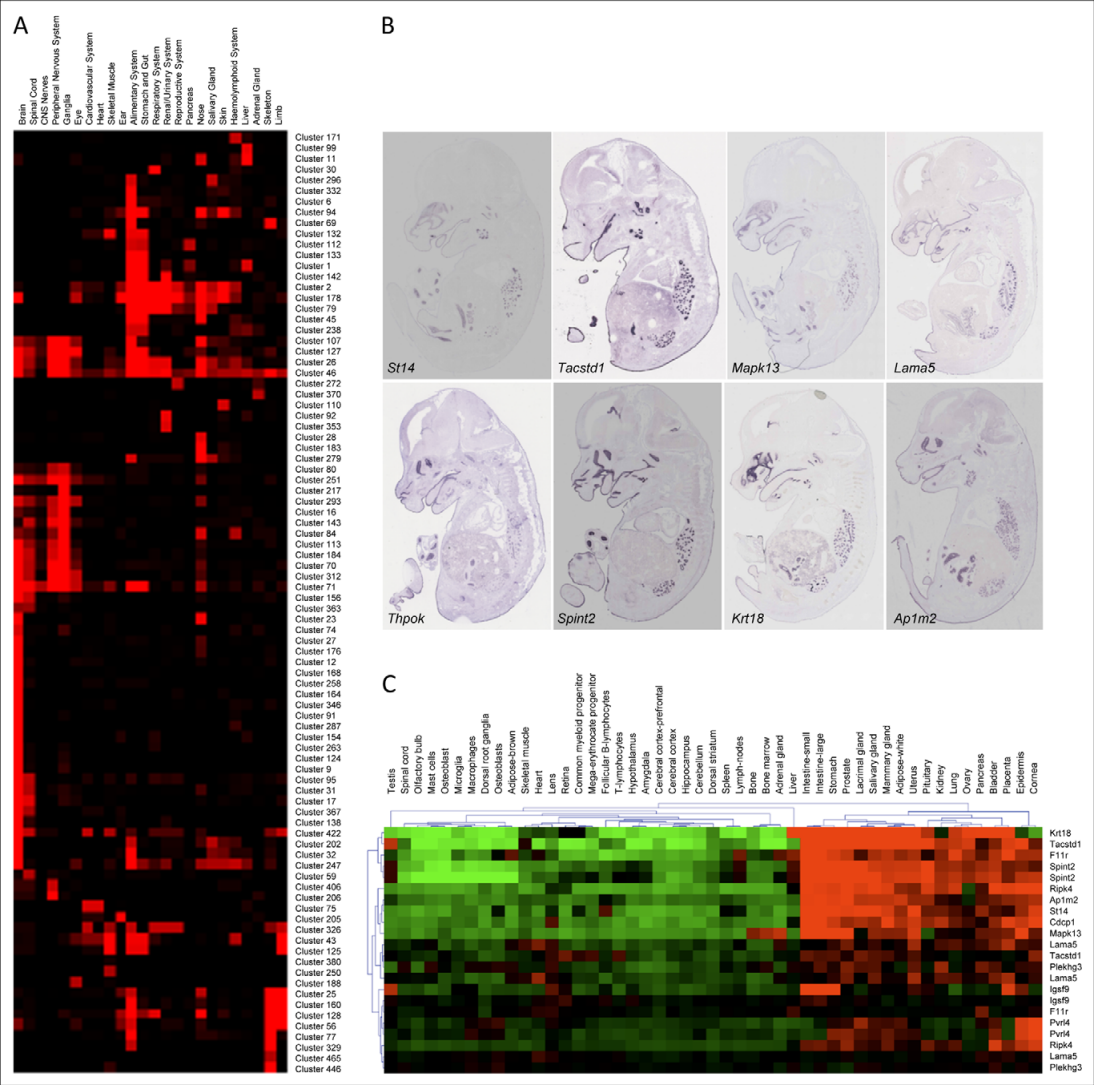
Hierarchical clustering of regionally expressed genes. (A) Graphical representation of clusters (listed on the right) with more than eight genes in terms of expression occupancy. The occupancy is calculated as the number of genes in each cluster that are expressed in the anatomical structures (listed at the top) divided by the number of genes in that cluster (normalization). The matrix of occupancy values for each tissue group clusters with tissue distribution. More information on clustering can be found at http://www.eurexpress.org/ee/project/publication/PlosBiol2010.html. (B) Cluster 83, with a Pearson coefficient of 0.73, is composed of eight different genes showing expression in epithelia (oral and nasal cavities, respiratory tract, and middle and internal auditory cavities), choroid plexus, and middle-gut mucosa. (C) Genes in Cluster 83 are also synexpressed in adult tissues. Publicly available microarray data (http://symatlas.gnf.org) were clustered using the MeV program (http://www.tm4.org/mev.html). The figure shows synexpression in intestine, stomach, lacrimal gland, salivary gland, uterus, prostate, mammary gland, placenta, and bladder. Note that some tissues listed on the top of the diagram are duplicated because they represent two independent datasets. Gene symbols are on the right.

Hierarchical clustering of expression data is a powerful tool to assess synexpression, with the ultimate goals of elucidating transcriptional pathways and dissecting gene co-regulation mechanisms. We decided to apply this methodology to our expression atlas. Towards this goal, a subset of 5,933 regionally expressed genes was clustered according to the tissue annotations across 831 anatomical terms. For each gene, an expression value was set according to the expression strength. For hierarchical clustering we then used the Pearson correlation coefficient, which means the actual selected values are normalized and only relative expression strength across the tissues is used. Clustering by annotation identified numerous synexpression groups, i.e., genes with coordinated expression and that are potentially involved in the same biological process. At a threshold value of the Pearson coefficient of *r*≥0.7, we found 496 clusters, 90 of which included at least ten genes (additional information available at http://www.eurexpress.org/ee/project/publication/cluster.jsp). We determined the expression occupancy of these clusters, which provides a measure of how many of the genes in a cluster are expressed in a specific anatomical structure. This approach allowed us to group clusters expressed in the same sets of tissues (Figure 4A), thus facilitating the identification of complex synexpression groups. Figure 4B shows an example of a cluster with a complex expression pattern (cluster 83). We found that genes in this cluster continue to be synexpressed in the adult (Figure 4C), as assessed by analysis of publicly available microarray data. This case raises the possibility that embryonic expression patterns have predictive value for adult mice. The clusters can be browsed online at http://www.eurexpress.org/ee/project/publication/cluster.jsp, a Web link that also provides interactive access to the gene lists and associated assays, and the results of the functional enrichment analysis with respect to Gene Ontology (GO), InterPro domains, Mammalian Phenotype Ontology, and cytogenetic band mappings. The individual cluster Web pages are also accessible directly from each assay view via the “Syn-Expression” link on the assay Web page (e.g., http://www.eurexpress.org/ee/databases/assay.jsp?assayID=euxassay_009028). The identification of these expression clusters will facilitate the dissection of transcriptional networks by integrating the high-resolution power of RNA ISH with the currently available high-throughput—but generally low-resolution—procedures such as microarray and next generation sequencing.

To gain insight into the dynamics of gene expression in the embryo versus the adult, we took advantage of the ABA dataset [8]. We compared gene expression patterns of 80 genes we found to be confined to the following CNS structures: cerebral cortex, striatum, thalamus, hypothalamus, midbrain, cerebellum, pons, medulla, and spinal cord (taken from Table S3). We found that 26% of the genes had a conserved expression pattern, 43% had extended their expression pattern into new domains of the adult brain, and 30% were divergent (Table S5). Figure S3 shows two examples for partial (Figure S3A and S3B) and full conservation (Figure S3C and S3D) of expression sites. A similar comparison was done for a subset of the solute carrier family of genes (*Slc*) for which a cognate ABA dataset was available (99 genes in total). Concordance for this data set was 89% (Table S6). Figure S4 illustrates examples where a particular *Slc* was expressed in progenitor (E14.5) and differentiated (adult) cells. In the future, gene expression at cellular resolution, refined by double-labeling experiments with specific cell type markers, will uncover to what extent gene expression networks are conserved across stages.

The Eurexpress atlas is highly informative with regard to expression patterns of disease-causing genes. We selected 100 disease genes that are representative examples of genes responsible for either diseases targeting specific tissues (e.g., eye, skeletal muscle, heart, skeleton, immune system) or syndromic conditions affecting multiple tissues. This analysis was carried out by comparing the information present, for each disease, in the clinical synopsis section of the Online Mendelian Inheritance of Man (OMIM) database with the gene expression annotation data present in Eurexpress. In all cases the expression pattern observed was predictive for the phenotypes seen in human (Table S7; Figure S5).

The above-described comparative analyses between embryonic and adult brain and the foray into expression of human disease genes emphasize that the reach of Eurexpress is well beyond the mid-gestation mouse embryo.

### Wnt Signaling in the Developing Kidney

**Figure 5:**
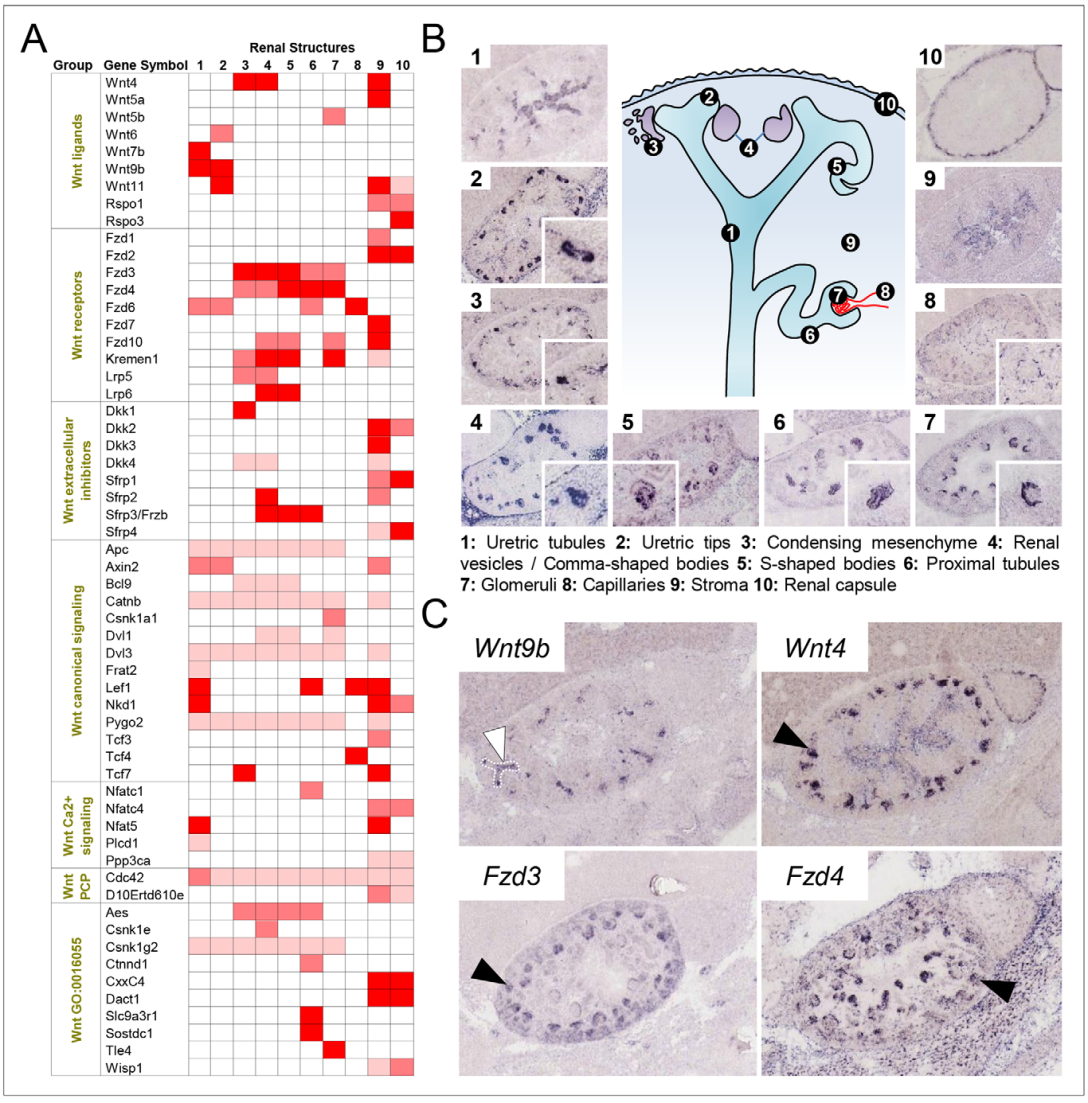
Expression sites of Wnt signaling components in the E14.5 mouse kidney. (A) The matrix shows the level of expression of all 58 regionally expressed genes in ten different renal structures that are defined in (B). Colors represent expression strength: strong (red), moderate (light red), weak (pink), and not detected (white). The Wnt signaling components are grouped into seven blocks (ligands, receptors, extracellular inhibitors, canonical signaling, Ca^2+^ signaling, PCP signaling, and GO Wnt receptor signaling pathway). (B) The scheme in the center illustrates the ten main anatomical structures characterizing the developing kidney. The image gallery composed of low- and high-power (inset) images reveals that each of the ten structures characteristically expresses a particular Wnt component. 1: *Wnt7b*; 2: *Wnt11*; 3: *Dkk1*; 4: *Sfrp2*; 5: *Lrp6*; 6: *Slc9a3r1*; 7: *Tle4*; 8: *Tcf4*; 9: *Wnt5a*; 10: *Rspo3*. (C) Wnt signaling components involved in the mesenchyme-to-epithelium transition. *Wnt9b* is expressed in the ureteric bud (white arrowhead) and acts upstream of WNT4, which is expressed in condensing mesenchyme (black arrowhead). The Wnt receptors FZD3 (black arrowhead) and FZD4 (black arrowhead) are expressed in a way that allows them to function as candidate transducers for WNT9B/WNT4 signaling and could possibly underlie a shift from canonical to noncanonical signaling.

Wnt signaling in embryogenesis is characterized by an extensive crosstalk between ligands, receptors and co-receptors, regulators, and downstream messengers [21]. Surprisingly, the expression patterns for many of the newly identified Wnt pathway components are largely elusive, a gap in knowledge Eurexpress begins to close. Table S8 summarizes the expression patterns of 117 Wnt signaling components for the major organ systems. Collectively these data illustrate which components are expressed in a given tissue and thus are an entryway into the identification of organ-relevant pathways. In the developing kidney, 58 genes of the Wnt signaling pathway show regional expression. Figure 5A displays the expression strength of these genes in ten renal structures that are recognizable at E14.5. The scheme in Figure 5B illustrates that the different steps of nephron formation occur concurrently at this stage. An early event is the induction of the condensing mesenchyme (Figure 5B, image 3), which subsequently undergoes a mesenchyme-to-epithelium transition leading to the development of the renal vesicle (Figure 5B, image 4). This process involves WNT9B and its downstream target WNT4 [22]. Consistent with published data [22], *Wnt9b* and *Wnt4* are expressed in the ureteric bud and the condensing mesenchyme (white and black arrows in Figure 5C). In addition to WNT4, we identified seven Wnt signaling components that were markedly expressed in the condensing mesenchyme (Figure 5A, column 3) and in cells involved in the mesenchyme-to-epithelium transition. Among them are *Fzd3* and *Fzd4* (Figure 5C, black arrows), which are both expressed in the appropriate place and time to potentially mediate downstream effects of paracrine WNT9B and autocrine WNT4 signals. The condensing mesenchyme expresses essential components of the canonical β-catenin-dependent pathway such as the Wnt co-receptor *Lrp5* and the transcription factor *Tcf7* (Figure 5A). Additionally, regulators of canonical signaling such as DKK1 and its receptor, KREMEN1, as well as AES, a repressor competing with β-catenin for binding to transcription factors, are expressed (Figure 5A). We noticed that *Fzd3* is prominently expressed in structures of early nephrogenesis (Figure 5A, columns 3–5), while *Fzd4* expression is more pronounced in the renal vesicle and in structures derived from it, such as the proximal tubules (Figure 5A, columns 5–7). This observation could support the idea of a receptor-mediated switch from canonical to noncanonical signaling thought to occur at the beginning of tubulogenesis [23]. We conclude that the comprehensive nature of the Eurexpress database allows one to select those components of signaling pathways that are expressed at the right time and location.

### Hematopoietic Stem Cell Lineages in Liver

Many of the regulators that control hepatocyte and cholangiocyte differentiation [24] are represented in the Eurexpress database. In total, 147 genes were largely confined to liver (Table S3), and these will provide markers to investigate liver development, especially at later stages. In the embryo, hepatocytes are closely associated with hematopoietic stem cells (HSCs). During fetal development, HSCs change anatomical localization several times and are abundant in liver between E10 and E18, with HSC cell number peaking at ∼5,100 around E14.5 [25],[26]. At E14.5, HSC markers such as *Itgab2* (CD41), *Ptprc* (CD45), *Ly6a* (Sca1), *Kit* (CD117), *Runx1,* and *Gata2* are strongly expressed in single, discrete cells scattered throughout the liver. Cells expressing these bona fide markers can be classified into three categories (Table S9): (1) in the case of *Gata2*, *Itgab2*, and *Runx1*, intercellular distance (*d*) is much larger than the cell diameter (cd) (*d*≫cd); (2) *Ly6a*-positive cells also obey this rule but in addition tend to form small clusters and intercluster distances are much larger than cd; and (3) cells expressing *Kit* or *Ptprc* are in proximity to each other (*d*≈cd). We mined the transcriptome atlas for genes whose expression patterns in liver fall into the above groups. Table S9 lists the members of these groups and, in addition, defines a fourth group of scattered cells where *d*≤cd. Collectively, these groups contain many genes that are implicated in immune functions encoding membrane-bound cell surface receptors, extracellular proteins, transcription factors, extracellular cytokines, protease inhibitors, focal adhesion proteins, and proteins generally involved in cell adhesion. Many of our markers tag a few thousand cells per liver, corresponding to the HSC number estimates for fetal liver [27], which raises the possibility that they identify HSCs. However, double-labeling analyses will be required to resolve which markers (or marker combinations) actually identify HSCs and which their descendants.

### Molecular Organization of the CNS

**Figure 6:**
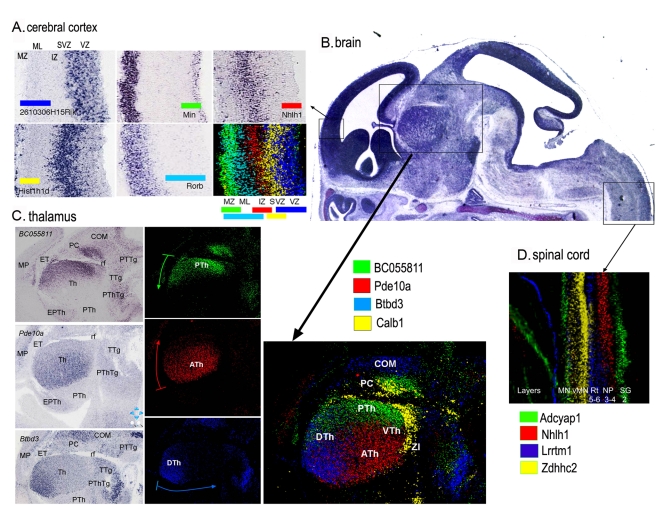
High-resolution molecular regionalization in the central nervous system. (A) Genes expressed in cells at different radial levels in the anterior pole of the dorsal pallium (presumptive frontal cortex). *2610306H15Rik* and *Hist1h1d* are localized at different apico-basal levels of the ventricular epithelium (VZ); *Nhlh1* is expressed at the subventricular zone (SVZ) and intermedial zone (IZ); *Nin* and *Rorb* are expressed in cells localized at different radial levels of the mantle layer (ML). Each transcript is depicted with a different color to show how the expression of each gene in pallial cells is complementary to others, with some degree of overlap. MZ, marginal zone. (B) Picture of a mid-sagittal section of the brain from a section series of a Eurexpress assay processed with Cresyl violet. The inserts show the area where the corresponding regions (arrows) have been localized. It is important to note the homogeneity of cellular patterns in the mantle layer of the thalamus and spinal cord, as opposed to the complex molecular patterns observed in (C) and (D). (C) Examples of three genes with a graded expression in the thalamic mantle layer (Th). *BC055811* shows strong expression in the caudal pole of the thalamus (close to the retroflexus tract [rf]), becoming weaker towards the anterior pole; *Pde10a* expression is complementary to that of *BC055811*, with a strong signal at the anterior pole of the thalamus, showing a sharp edge of its expression domain at the limit with the prethalamus (PTh). The expression of this gene becomes progressively weaker towards the caudal pole. *Btbd3* transcripts have a dorso-ventral decreasing gradient, strong at the dorsal thalamus and progressively weaker towards the ventral thalamus. The ventral pole of the thalamic mantle layer is depicted by the expression of *Calb1*. The merged picture, using a color for each gene (right panel), shows how molecular regionalization allows detection of differences in cell identities in the four areas of thalamic mantle layer: dorsal (DTh), anterior (ATh), ventral (VTh), and posterior (PTh) thalamus. COM, commissural nuclei of pretectum; EPTh, eminentia thalami; ET, epithalamus; MP, medial pallium; PC, precommisural nuclei of pretectum; PThTg, prehalamic tegmentum; PTTg, pretectal tegmentum; TTg, thalamic tegmentum; ZI, zona incerta. (D) Sagittal section of the spinal cord, showing an overlay picture where the expression patterns of four genes have been combined. The picture summarizes the localization of region-specific molecular codes in spinal cord cells. These molecular codes correspond to different structural levels of the developing spinal cord: *Adcyap1* is expressed in the gelatinous substance (SG, Rexed's layer 2) and motoneurons (MN); *Nhlh1* is expressed in the spinal cord in the central nucleus of the dorsal horn (NP, Rexed's layers 3 and 4); *Lrrtm1* is located in the spinal reticular nucleus (Rt, Rexed's layers 5 and 6); and *Zdhhc2* is located in visceral motoneurons (vMN). Note that the expression patterns reported above, with the exception of *Rorb* and *Calb*, are novel. The merged color composites are the product of alignment, superposition of sections, and editing using a computer program. A detailed description of the methods used to obtain such figures is included in Text S1.

In the E14.5 embryo, most neurons of the CNS have been generated and have migrated from the germinative epithelium into the mantle layer. However, important migratory processes that shape the future CNS have not yet initiated. Thus, this atlas is a rich source of additional gene markers that characterize diverse neuronal populations. Figure 6A shows examples of expression patterns of five genes collectively delineating the stratification of the nascent neocortex. *2610306H15Rik* and *Hist1h1d* are localized at different apico-basal levels of the ventricular epithelium, *Nhlh1* is expressed in the subventricular and intermediate zones, and *Nin* and *Rorb* are expressed in cells localized at different radial levels of the mantle layer.

At E14.5, the complex cytoarchitecture of the mature spinal cord is not evident, although most neurons have been generated and have migrated into the mantle layer. To date, many molecular markers for the motoneuron columns have been identified in the ventral horn [28], but there are few markers for the central zone and for the dorsal horn that do not show any internal subdivisions and appear as homogeneous cellular fields. We found that expression patterns of four genes revealed molecular differences of neurons at different ventro-dorsal levels along the length of the spinal cord (Figure 6D). *Nhlh* and *Lrrtm1* are expressed at different layers of the dorsal horn, *Adcyap1* is expressed in the dorsal-most cells of the dorsal horn and in motoneurons, and *Zdhhc2* is mainly expressed in visceral motoneurons. These cellular populations that show different molecular expressions may belong to the primordium of Rexed's lamina in the mature spinal cord [29].

The thalamus also appears as a homogeneous cellular field at E14.5, except for the thalamo-cortical fiber confluence (Figure 6B). Mining the transcriptome digital atlas allowed us to detect genes marking an early molecular regionalization of the thalamic mantle layer, where undifferentiated neurons accumulate. Figure 6C, shows four examples of genes that show graded expression with respect to putative diencephalic “secondary organizers” that are the basal plate and zona limitans (as sources of SHH ventralizing and rostralizing signals) and the dorsal midline (which produces FGF8, BMPs, and Wnt dorsalizing signals) [30]. These intra-thalamic regionalized genes may specify different cell fates in a concentration-dependent manner and thus underlie the development of functional domains in the mature thalamus.

The developing mammalian CNS is characterized by complex gene expression patterns, and the interpretation of these data has led to the prosomeric model of the mammalian brain [31]. This model predicts the existence of domains within the ventricular zones that give rise to diverse segments and morphogenetic fields [31]. We mined the digital expression atlas for genes that have a restricted expression pattern within the ventricular zone along the rostral–caudal axis and hence could be involved in early specification of the pallial domains of the telencephalon [31]. Nissl staining showed a mainly homogeneous cellular organization along the midline (Figure S6A) and progressive lateral sections of the telencephalic pallium (Figure S6C and S6E). Gene expression patterns clearly demonstrated a molecular heterogeneity among different regions at the level of the ventricular epithelium and mantle layer in the corresponding midline (Figure S6B) and lateral sections (Figure S6D and S6F), thus mapping the predicted molecular regions in the subpallium and pallium. For instance, while a new marker gene (*0610040j01Rik*) showed a localized expression in the medial pallium epithelium (prospective hippocampus), *Dct* and *Zic3* were expressed in progressively more anterior neuroepithelial domains (the prospective progenitors for lateral pallium and ventral pallium, respectively) (Figure S6C–S6F).

Moreover, hierarchical clustering of brain-specific transcription factors (using the approach described in Figure 4) revealed a group of ten transcription factor genes that show co-localized or complementary expression patterns in the telencephalic pallium and subpallium (*Nfe213, Hivep2, Klf7, Fos12, Satb2, Zfhx1b, Zfp184, Foxp4, Phf13*, and *Dmrtal*). Therefore, the intricate organization of molecular markers identified allowed us to develop combinatorial maps that represent the molecular organization of the telencephalon (Figure S6B, S6D, and S6F). At the same time these markers will provide an entryway into future genetic fate mapping strategies.

**Figure 7:**
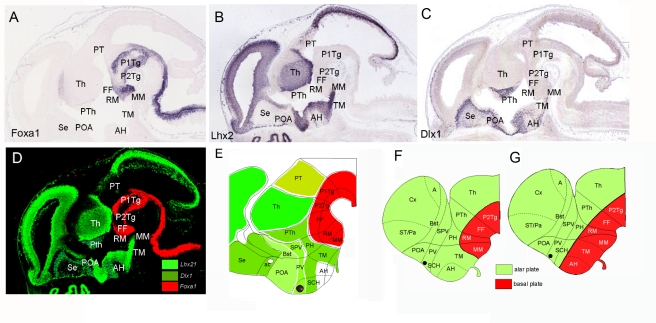
Combinatorial analysis of several transcription factors' patterns in the hypothalamus reveals a new model of mammalian hypothalamic organization. (A) *Foxa1* expression pattern in the basal plate of rhombencephalic, mesencephalic, diencephalic, and caudal hypothalamic neuroepithelium. This pattern is representative of other transcription factors such as *Lmx1a, Lmx1b, Barhl1, Dbx1, Pax7, Olig2, Rarb, Dfp3, Lhx1, Lhx5, Irx1*, and *Irx3*, expressed in the prosencephalic basal plate, including hypothalamus, where they were exclusively localized in the caudal regions: mammillar (MM) and/or retromammillar (RM) areas. (B and C) *Lhx2* and *Dlx1* expression patterns are representative of transcription factors expressed in alar prosencephalic derivatives (telencephalon, prethalamus, and thalamus) showing expression in TM and AH areas (currently described as basal plate hypothalamic domains), as well as in alar hypothalamic regions such as the suprachiasmatic (SCH), paraventricular (PV), and supraopto-paraventricular (SPV) areas. These patterns are representative of other genes expressed in alar derivatives including the TM and AH regions: *Lhx6, Lhx9, Dlx1, Dlx2, Dlx5, Unc4, Cited, Rorb, Arx, Foxa2*, and *Otx2*. (D) Photoshop composition to illustrate the alar expression patterns of *Lhx2* and *Dlx1* (green) and the *Foxa1* basal expression (red). (E) Schematic representation of the analyzed patterns suggesting that the mammillar and retromammillar areas show basal plate molecular characteristics, while the TM and AH regions showed alar plate molecular characteristics. (F and G) Representation of the new revised topologic model that incorporates the TM and AH regions into the alar plate (F), compared to the currently accepted prosomeric model (G).The merged color composites are the product of alignment, superposition of sections, and editing using a computer program. A detailed description of the methods used to obtain such figures is included in Text S1. A, amygdale; ac, anterior commissure; Bst, bed nucleus of stria terminalis; Cx, cortex; FF, forel fields; P1Tg, pretectal tegmentum; P2Tg, thalamic tegmentum; PH, posterior hypothalamic area; POA, preoptic area; PT, pretectum; PTh, prethalamus; PV, paraventricular; SCH, suprachiasmatic; Se, septum; SPV, supraopto-paraventricular; ST/Pa, striatum/pallidum; Th, thalamus.

Given that this combinatorial analysis of expression patterns in the developing diencephalon mainly agrees with previously proposed molecular maps [31]–[33], we were interested to explore the efficiency of this approach for studying regionalization and topology in the hypothalamus, where controversial models have been postulated [31],[32],[34] (see [35] for a review). Using the digital atlas we selected expression patterns of genes encoding DNA binding proteins that showed “regional expression” (1,395 genes) and analyzed in detail the expression of 126 of them expressed in brain. This analysis revealed that genes mainly expressed in the basal plate domains of the diencephalon, including the hypothalamus, were exclusively expressed in the caudal hypothalamic regions: mammillar region and retromammillar areas (13 genes were identified with this pattern: *Foxa1, Mx1a, Lmx1b, Barhl1, Dbx1, Pax7, Olig2, Rarb, Dfp3, Lhx1, Lhx5, Irx1, and Irx3*; Figure 7A and 7D). Conversely, genes mainly expressed in the diencephalic alar plate and/or in the telencephalon extended their expression into the tuberomammillar (TM) hypothalamus and/or anterior hypothalamic (AH) and suprachiasmatic nucleus (12 genes were identified with this pattern: *Lhx2, Lhx6, Lhx9, Dlx1, Dlx2, Dlx5, Unc4, Cited, Rorb, Arx, Foxa2, and Otx2*; Figure 7B–7D). Thus, this analysis revealed that both mammillar and retromammillar regions express genes of generic basal plate character, while the TM, AH, and suprachiasmatic hypothalamic areas, although classified as basal plate derivatives, express mainly “alar” genes. The expression analysis of the developing hypothalamus strongly suggests that the TM hypothalamus (including the neurohypophysis) and the anterior hypothalamus have an alar plate character. The expression patterns of *Shh* and *Nkx2.1* in the tuberal and the AH areas could be used against this new interpretation [31]. However, grafting data showed different inductive properties of diencephalic and hypothalamic SHH signals [36], suggesting that these differences in SHH signaling could be attributed to its alar and basal nature. In conclusion, our data suggest a novel regional map of the hypothalamus (Figure 7E and 7F) that interprets the data more appropriately than the previous model [31] (Figure 7G) and that allows us to understand the different inductive effects of the anterior axial mesoderm in the anterior neural plate [37] and its ability to induce basal plate and alar plate derivatives. More interestingly, this new interpretation that places primary sensorial hypothalamic areas (i.e., AH and TM areas [38]) as alar plate derivatives agrees with the hypothesis of “functional columns” in the vertebrate brain, where sensorial information is primarily processed by alar derivatives (extensively reviewed in [39]).

## Discussion

This is to our knowledge the first gene expression atlas of an entire mammalian organism that is thoroughly annotated so as to systematically capture gene expression in hundreds of organs and tissues. Because all this information is available in a searchable database, users can retrieve information tailored to their own needs. The present study provides a selection of examples demonstrating how this resource can be applied to a broad range of biomedical questions and drive scientific discovery. We showed that we can correlate disease phenotypes to sites of expression of underlying genes; we extracted information to demonstrate novel insights into the complex segmental organization of the mammalian brain; the cellular resolution provided by the Eurexpress atlas enabled the discovery of gene markers that characterize the molecular subdivision of organs, identified novel putative markers of the hematopoietic lineage, and facilitated the comprehensive organism-wide mapping of an important developmental signaling pathway. Future applications of these data might include the determination of elusive regional differences within structurally complex organs, the identification of expression signatures for specific cell populations, the search for regulatory elements that confer tissue- or region-specific expression, the establishment of gene networks that operate within and between organs, the molecular characterization of genetic or otherwise modified mice, and the design of new tissue-specific CRE driver lines and cell lineage experiments. Finally, this atlas is ideal for the evaluation of candidate genes for complex diseases and congenital disorders.

## Materials and Methods

### Template Selection and Generation

For gene selection, both the mouse ENSEMBL and the mouse Entrez Gene databases were analyzed. Templates used for the generation of the atlas were PCR products obtained from either publicly available cDNA clones or reverse transcriptase PCR reactions, a fraction of which was provided by the ABA consortium [8]. Automated ISH was performed using previously described protocols [7]. We set up semi-automated routines for designing one appropriate probe per gene (Figure S1). Our approach was aimed at covering most of the genes represented in public mouse databases (ENSEMBL and Entrez Gene). Because of the high-throughput nature of the project, we restricted our selection to one probe per gene, capturing most of the isoforms generated by alternative splicing, when possible. As an initial source of DNA for PCR template generation, we used cDNA clones (IMAGE collection or Mammalian Gene Collection) that were available and re-sequenced at the German Resource Center for Genome Research (RZPD). Approximately 10,000 clones could be used for template generation. The clones were used as direct templates for PCR and stored as glycerol stock in 384-well plates at −80°C. This initial collection was then enlarged to include about 8,000 PCR templates generated from the ABA consortium [8]. The latter templates were dilutions of first-round PCR products derived from EST clone, mouse brain cDNA, or mouse genomic DNA (ABA templates).

All clones or PCR template sequences were compared to the mouse gene reference databases (ENSEMBL and Entrez Gene) via BLAST (http://www.ncbi.nlm.nih.gov/BLAST/) prior to selection. For the probe generation we selected only templates with sequences matching the reference with at least 95% identity across at least 80% of the length. Templates were generated by PCR using appropriate oligonucleotide primers. Full information on templates, including the complete sequence of the product, the sequences of the oligonucleotides used to generate them, and the RNA polymerase promoters used for riboprobe synthesis, are available on the Eurexpress Web site.

PCR reactions were performed in a 100- µl total volume with final concentrations of 1× Taq buffer, 1.5 M Betaine, 0.2 mM dNTPs, 5 U Taq polymerase, 10 U Pfu DNA polymerase, and 0.5 µM of each primer. As template material for the PCR, we used clone glycerol stock, purified plasmid, or PCR product (ABA collection).

The quality (size and quantity) of the PCR templates was systematically assessed by standard gel electrophoresis (1% agarose gel) and by spectrophotometry (Nanodrop). PCR products yielding an unexpected size (±100 bp) or showing multiple bands were excluded from riboprobe generation.

In vitro transcription was performed as previously described [5].

### Data Annotation

Approximately 360,000 images were viewed and annotated, each of high resolution and typically 4K×4K pixels. To allow the annotators to rapidly pass through the data and assess each image, we implemented a bespoke annotation Java-based interface termed Fast Image Annotation Software (FIATAS). Key aspects of the software are the fast interfaces for image viewing, focused anatomy views with efficient menu and multi-select option annotation, data “inbox” management, quality control and multi-editor review, and automatic update to the tracking database and publication to the Web site (Figure S8). FIATAS can be installed for off-line operation or will start directly via Web-start from links on the Eurexpress Web site.

For anatomy tissue annotation we adopted the standard mouse ontology from EMAP. In the FIATAS interface, the full anatomical tree of 1,420 terms at Theiler stage 23 is provided, as well as a number of cut-down views, which can be used for more detailed access. More information on data annotation can be found in Text S1.

### Data Management

The link between the central database and each activity was managed via a combination of Web services and ftp, with data exchanged either in XLS, XML or JPEG formats. The architecture is shown in Figure S7.

### Cluster Analysis

Functional inference using Eurexpress data employed hierarchical clustering with centered Pearson correlation coefficients and the average linkage method. We employed a maximal propagation strategy, where parent terms acquire the values of child terms throughout the anatomical ontology. Four annotation types were examined: GO terms, InterPro conserved domain identifiers, Mammalian Phenotype Ontology terms, and cytogenetic band (as a proxy for genomic position). Annotation enrichment was calculated for each co-expressed cluster containing ten or more genes (to ensure sufficient annotation to carry out tests), and the significance of each test was measured using the hypergeometric distribution according to the standard practice. The significance of enrichment across all clusters in the dataset was determined using a permutation strategy: 100,000 permuted datasets were produced by permuting gene IDs with respect to their annotation, but maintaining GO term interdependencies. The numbers of tests passing given *p*-value thresholds, within each permuted dataset, were then used to calculate the significance of tests passing those thresholds in the observed dataset. This proportion provided us with a permutation-derived *p*-value, which accounted for the large number of tests performed while controlling for the interdependencies among the GO annotation terms.

The Eurexpress Web site has implemented a link to visualize clusters of co-expressed genes derived from hierarchical clustering of Eurexpress anatomical expression patterns. In each case the relevant cluster ID is given together with the average correlation coefficient between genes in the cluster, the number of genes within the cluster, and the IDs of the genes involved. Further information on the enrichment of functional annotation within each cluster is available to users by clicking on the cluster IDs. This information includes the annotation terms and enrichment *p*-values for the GO terms, the InterPro domains, the Mammalian Phenotype Ontology terms, and the cytogenetic band mappings.

## Supporting Information

Figure S1 **Eurexpress template generation and riboprobe synthesis workflow.**
(0.07 MB PDF)

Figure S2 **Transcriptome complexity of main organs and anatomical structures.** The bars represent the number of genes displaying a regional expression pattern in selected organs and structures.
(0.03 MB PDF)

Figure S3 **Comparison of expression patterns for E14.5 CNS-specific genes between embryonic and adult brain.** This figure illustrates two examples of degrees of similarity between fetal and adult brain. (A and B) show partial concordance of the expression pattern of the RFamide-related peptide gene in neurons of the dorsomedial hypothalamic nucleus (DM) at E14.5 (A) and adult (B). (C and D) show coincidence of expression of the G-protein-coupled receptor 151 gene in the presumptive region of the habenular nuclei (MHb) (C) and the habenular region (MHb and LHb) (D).
(1.14 MB PDF)

Figure S4 **Comparison of expression patterns for E14.5 CNS-specific genes between embryonic and adult brain.** This figure illustrates typical cases of equivalent (A–F), partially equivalent (G), and different (H) patterns. Images shown were downloaded from either the Eurexpress database or the ABA. 4V, fourth ventricle; bv, brain vasculature; cb, cerebellum; cp, choroid plexus; cx, cortex; ep, ependyma; hy, hypothalamus; mb, midbrain; md, medulla; pcp, Purkinje cell progenitors; pcl, Purkinje cell layer; po, pons; sn, substantia nigra; st, striatum; th, thalamus; vta, ventral tegmental area; vz, ventricular zone. (A) The glutamate transporter SLC1A6 is expressed in Purkinje cell progenitors of the developing cerebellum as well as in all adult cerebellar Purkinje neurons. (B) Glucose transporter SLC2A1 expression persists in both embryonic and adult brain vasculature. (C) SLC4A2, a chloride/bicarbonate transporter, is characteristically expressed in the epithelial lining of the choroid plexi. (D) SLC6A3, a dopamine transporter, is highly expressed in the substantia nigra and its progenitor region, the ventral tegmental area. (E) Serotonin transporter SLC6A4 is strongly expressed in raphe nuclei of the embryonic and adult brain. (F) SLC17A6 resides in synaptic vesicles and takes up glutamate for subsequent release into the synaptic cleft. It is broadly expressed in neurons in the adult brain, and this pattern is already seen in the E14.5 brain. (G) The glial high-affinity glutamate transporter SLC1A3 is strongly expressed in the ventricular lining of the developing brain. Later, in the adult brain, expression is most prominent in astroglia scattered throughout the brain and in the Purkinje cell layer of the cerebellum (see overview article [40]). The characteristic cell shape of SLC1A3-positive adult glia cells is already seen in embryonic SLC1A3-positive cells, suggesting that these are glial progenitors already expressing a typical adult brain Slc. (H) SLC4A4, a sodium bicarbonate co-transporter, is highly expressed in ependymal cells lining the ventricular floor from the midbrain to the spinal cord, possibly regulating the electrolytic composition of the cerebrospinal fluid. In the adult brain SLC4A is expressed throughout the brain and co-localizes with glial cells. These rather different patterns of expression raise the possibility of distinct embryonic and adult functions for the proteins.
(2.27 MB PDF)

Figure S5 **Tissue distribution at E14.5 of the murine homologs of three human disease genes.** The human disease genes are *SALL1, GDF5*, and *SLC26A2*, responsible for Townes-Brocks syndrome, brachydactyly type C, and achondrogenesis type 1B, respectively. The expression observed is consistent with the phenotypic spectrum of the corresponding disease (see Table S7 for further details and for additional examples).
(1.69 MB PDF)

Figure S6 **Genoarchitecture of developing mouse forebrain Nissl-stained sagittal sections.** Midline (A) and progressively more lateral sections (C and E) illustrating the basic anatomy, with the pertinent anatomical structures labeled. (B, D, and F) show the same planes as in (A, C, and E) with expression patterns of several genes indicated by color. Names of genes are provided in the same colors used to delineate their sites of expression ([D] and [F] present the same genes). ac, anterior commissure; AH, anterior hypothalamus; ch, choroidal plexus; cp, commissural plate; DP, dorsal pallium; LGE, lateral ganglionic eminence; LP, lateral pallium; LT, lamina terminals; MGE, medial ganglionic eminence; ML, mantle layer; ML, mantle layer; MM, mammillar region; MP, medial pallium; OB, olfactory bulb; och, optic chiasm; POA, preoptic area; PTh, prethalamus; SCH, suprachiasmatic nucleus; Se, septum; Th, thalamus;VE, ventricular epithelium; VP, ventral pallium. The merged colored composites are the product of alignment, superposition of sections, and editing using a computer program. A detailed description of the methods used to obtain such figures is included in Text S1.
(0.22 MB PDF)

Figure S7 **Eurexpress data management architecture.** Each process on the outer pipeline is tracked by data exchange with the tracking database (TDB). The yellow arrows represent data flow using protocols as described in the test.
(0.66 MB PDF)

Figure S8 **Screen view of the FIATAS annotation interface.** The image displayed in the left-hand view can be expanded to full resolution and panned at will. The right-hand side image selector also shows which images are annotated. The upper, partially hidden dialog box shows the current “inbox” and which user is currently annotating which assay, and provides the review and quality control options. The small dialog box lower center provides the annotation options for the selected anatomical terms.
(1.42 MB PDF)

Table S1 **Comparison of independently produced ISH data for the solute carrier superfamily.**
(0.53 MB PDF)

Table S2 **Validation of Eurexpress data against published data.**
(0.21 MB DOC)

Table S3 **List of genes that display exclusive expression in selected structures.**
(0.10 MB PDF)

Table S4 **Distribution of genes with restricted spatial expression in different anatomical structures.**
(0.09 MB PDF)

Table S5 **Evaluation in the adult mouse brain of the expression of the genes expressed exclusively in the CNS at E14.5.**
(0.34 MB PDF)

Table S6 **Comparison of Slc expression patterns between embryonic and adult mouse brain.**
(0.36 MB PDF)

Table S7 **List of murine homologs of human disease genes whose tissue distribution at E14.5 is consistent with the corresponding human disease phenotype.**
(0.08 MB PDF)

Table S8 **Expression of Wnt signaling components in the E14.5 embryo.**
(0.09 MB PDF)

Table S9 **Classification of single cell expression patterns in the E14.5 liver.**
(3.64 MB PDF)

Text S1 **Supporting methods.** This file gives an overview of the methods used in this manuscript. Additional supplementary data on clustering can be found at http://www.eurexpress.org/ee.
(0.20 MB DOC)

## Abbreviations

ABA: Allen Brain Atlas
AH: anterior hypothalamic
CNS: central nervous system
E[number]: embryonic day [number]
EMAGE: Edinburgh Mouse Atlas of Gene Expression
EMAP: Edinburgh Mouse Atlas Project
FIATAS: Fast Image Annotation Software
GO: Gene Ontology
HSC: hematopoietic stem cell
ISH: in situ hybridization
MGI: Mouse Genome Informatics
TM: tuberomammillar
