# Supplementary figures and images for: A High-Resolution Anatomical Atlas of the Transcriptome in the Mouse Embryo
Source: PLoS Biol. 2011 Jan 18;9(1):e1000582. doi: 10.1371/journal.pbio.1000582 (PMC3022534; doi:10.1371/journal.pbio.1000582)

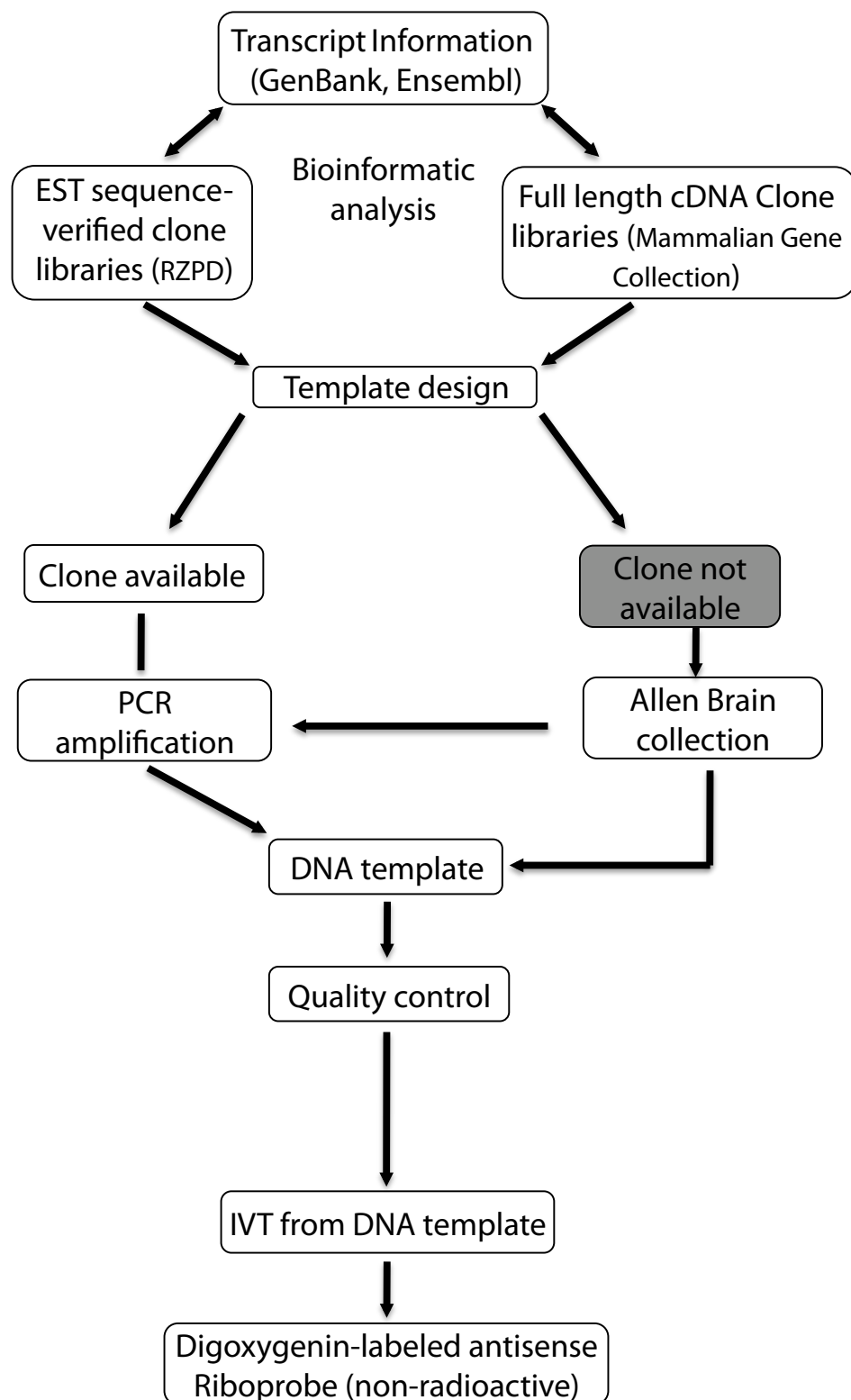

Figure S1

Supplement: Figure S1 — Eurexpress template generation and riboprobe synthesis workflow. (0.07 MB PDF) [file pbio.1000582.s001.pdf]

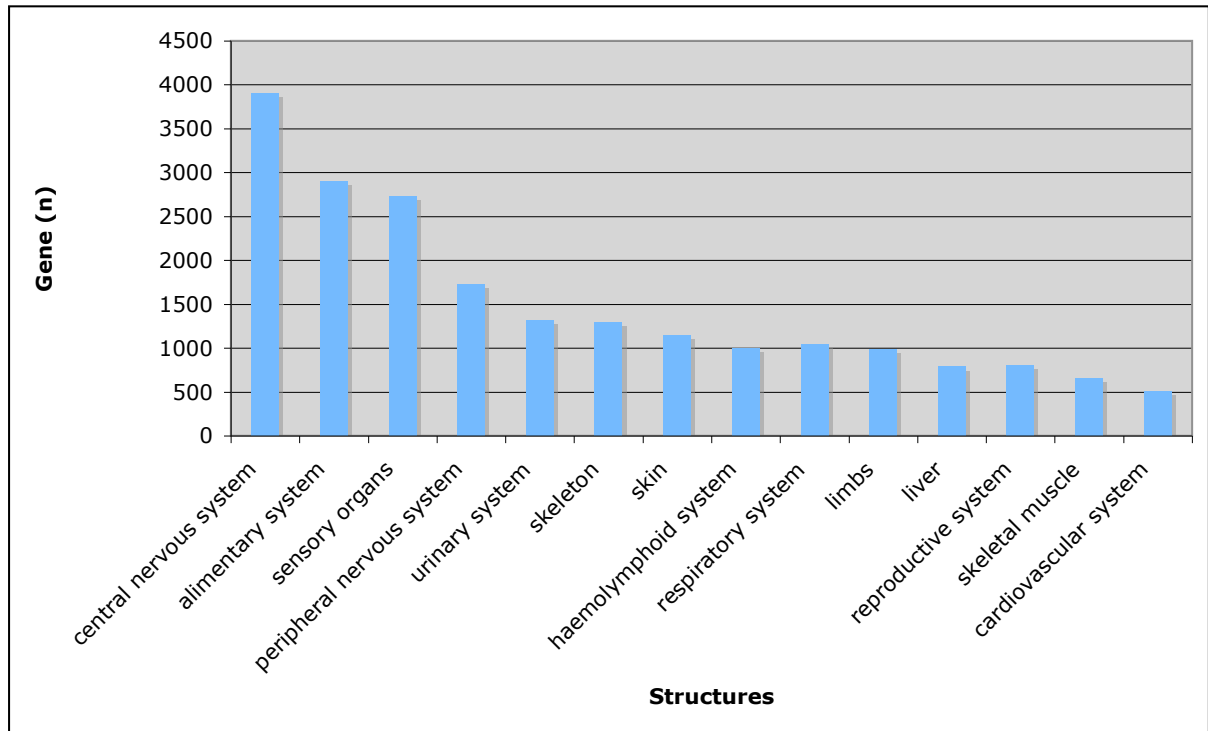

Figure S2

Supplement: Figure S2 — Transcriptome complexity of main organs and anatomical structures. The bars represent the number of genes displaying a regional expression pattern in selected organs and structures. (0.03 MB PDF) [file pbio.1000582.s002.pdf]

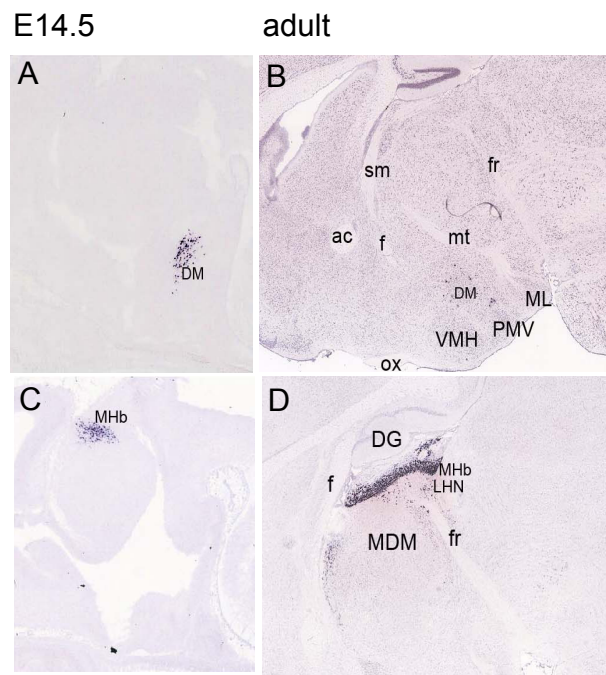

Figure S3

Supplement: Figure S3 — Comparison of expression patterns for E14.5 CNS-specific genes between embryonic and adult brain. This figure illustrates two examples of degrees of similarity between fetal and adult brain. (A and B) show partial concordance of the expression pattern of the RFamide-related peptide gene in neurons of the dorsomedial hypothalamic nucleus (DM) at E14.5 (A) and adult (B). (C and D) show coincidence of expression of the G-protein-coupled receptor 151 gene in the presumptive region of the habenular nuclei (MHb) (C) and the habenular region (MHb and LHb) (D). (1.14 MB PDF) [file pbio.1000582.s003.pdf]

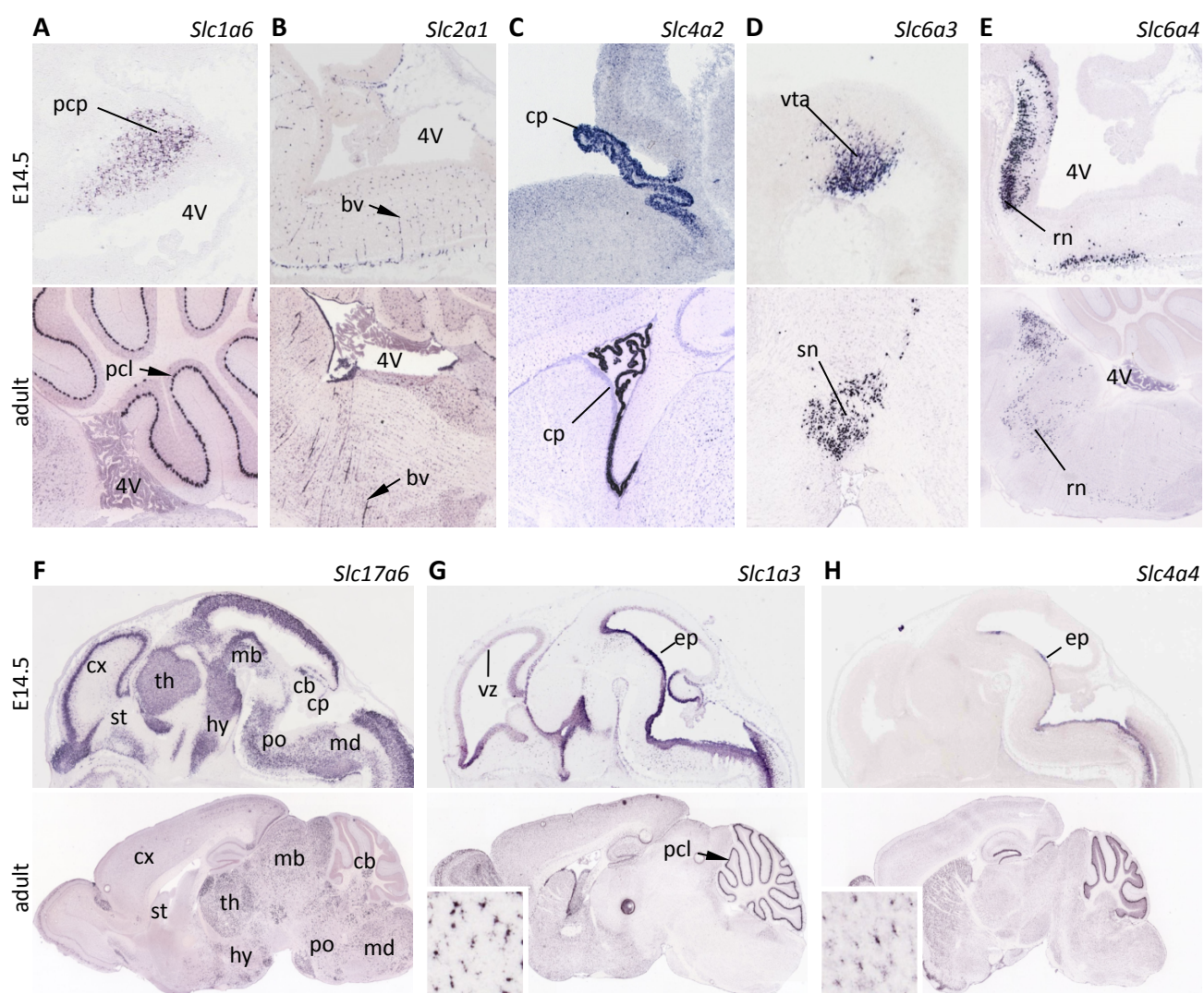

Figure S4

Supplement: Figure S4 — Comparison of expression patterns for E14.5 CNS-specific genes between embryonic and adult brain. This figure illustrates typical cases of equivalent (A–F), partially equivalent (G), and different (H) patterns. Images shown were downloaded from either the Eurexpress database or the ABA. 4V, fourth ventricle; bv, brain vasculature; cb, cerebellum; cp, choroid plexus; cx, cortex; ep, ependyma; hy, hypothalamus; mb, midbrain; md, medulla; pcp, Purkinje cell progenitors; pcl, Purkinje cell layer; po, pons; sn, substantia nigra; st, striatum; th, thalamus; vta, ventral tegmental area; vz, ventricular zone. (A) The glutamate transporter SLC1A6 is expressed in Purkinje cell progenitors of the developing cerebellum as well as in all adult cerebellar Purkinje neurons. (B) Glucose transporter SLC2A1 expression persists in both embryonic and adult brain vasculature. (C) SLC4A2, a chloride/bicarbonate transporter, is characteristically expressed in the epithelial lining of the choroid plexi. (D) SLC6A3, a dopamine transporter, is highly expressed in the substantia nigra and its progenitor region, the ventral tegmental area. (E) Serotonin transporter SLC6A4 is strongly expressed in raphe nuclei of the embryonic and adult brain. (F) SLC17A6 resides in synaptic vesicles and takes up glutamate for subsequent release into the synaptic cleft. It is broadly expressed in neurons in the adult brain, and this pattern is already seen in the E14.5 brain. (G) The glial high-affinity glutamate transporter SLC1A3 is strongly expressed in the ventricular lining of the developing brain. Later, in the adult brain, expression is most prominent in astroglia scattered throughout the brain and in the Purkinje cell layer of the cerebellum (see overview article [40]). The characteristic cell shape of SLC1A3-positive adult glia cells is already seen in embryonic SLC1A3-positive cells, suggesting that these are glial progenitors already expressing a typical adult brain Slc. (H) SLC4A4, a sodium bicarbon [file pbio.1000582.s004.pdf]

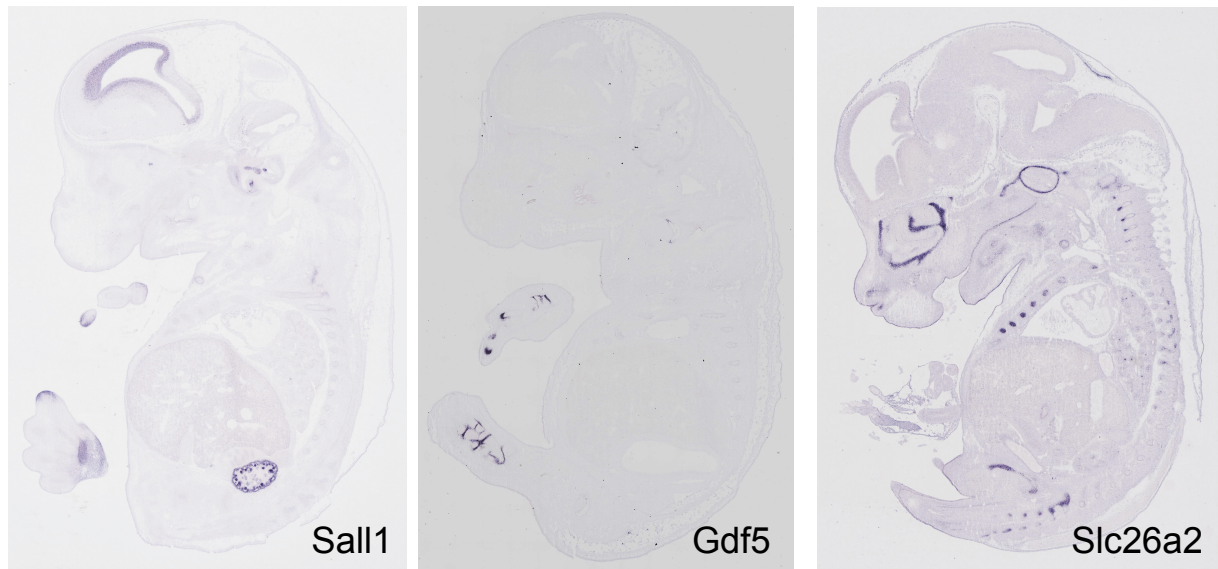

Figure S5

Supplement: Figure S5 — Tissue distribution at E14.5 of the murine homologs of three human disease genes. The human disease genes are SALL1, GDF5, and SLC26A2, responsible for Townes-Brocks syndrome, brachydactyly type C, and achondrogenesis type 1B, respectively. The expression observed is consistent with the phenotypic spectrum of the corresponding disease (see Table S7 for further details and for additional examples). (1.69 MB PDF) [file pbio.1000582.s005.pdf]

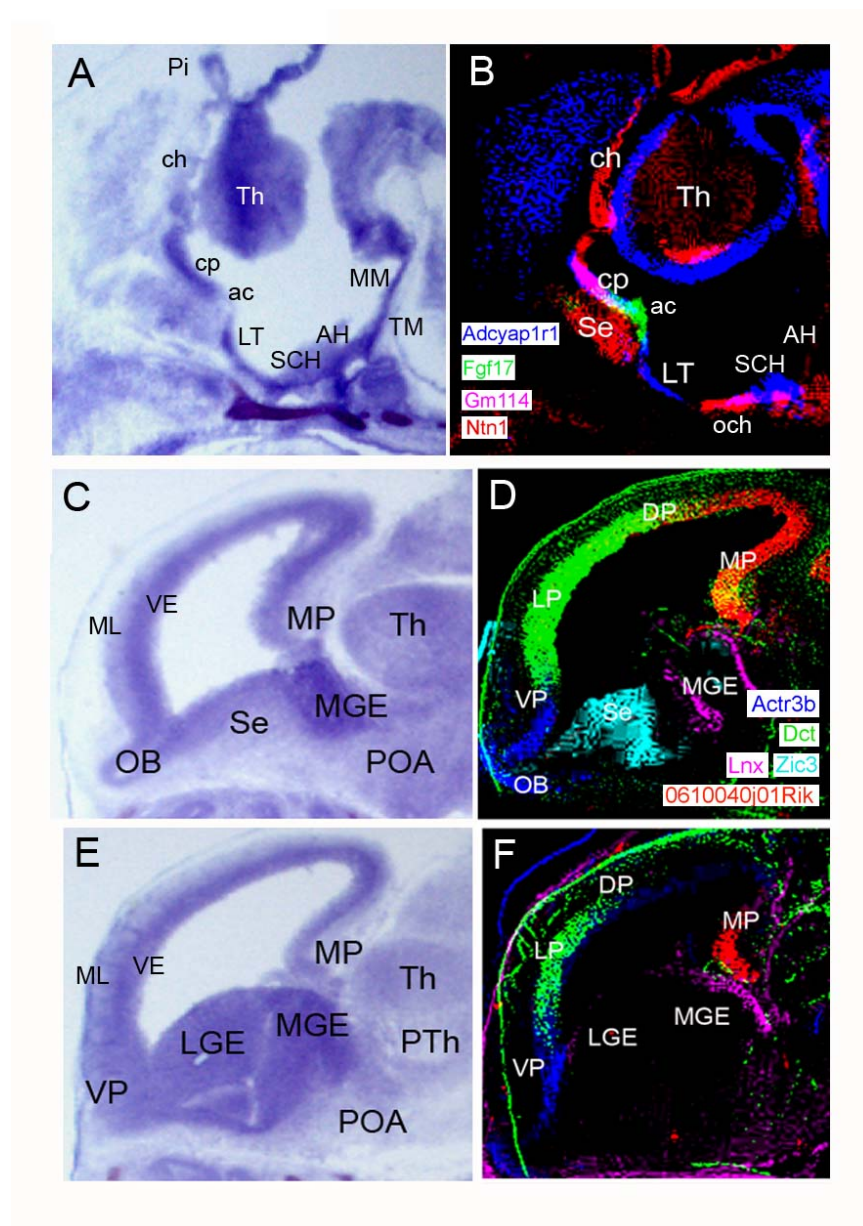

Figure S6

Supplement: Figure S6 — Genoarchitecture of developing mouse forebrain Nissl-stained sagittal sections. Midline (A) and progressively more lateral sections (C and E) illustrating the basic anatomy, with the pertinent anatomical structures labeled. (B, D, and F) show the same planes as in (A, C, and E) with expression patterns of several genes indicated by color. Names of genes are provided in the same colors used to delineate their sites of expression ([D] and [F] present the same genes). ac, anterior commissure; AH, anterior hypothalamus; ch, choroidal plexus; cp, commissural plate; DP, dorsal pallium; LGE, lateral ganglionic eminence; LP, lateral pallium; LT, lamina terminals; MGE, medial ganglionic eminence; ML, mantle layer; ML, mantle layer; MM, mammillar region; MP, medial pallium; OB, olfactory bulb; och, optic chiasm; POA, preoptic area; PTh, prethalamus; SCH, suprachiasmatic nucleus; Se, septum; Th, thalamus;VE, ventricular epithelium; VP, ventral pallium. The merged colored composites are the product of alignment, superposition of sections, and editing using a computer program. A detailed description of the methods used to obtain such figures is included in Text S1. (0.22 MB PDF) [file pbio.1000582.s006.pdf]

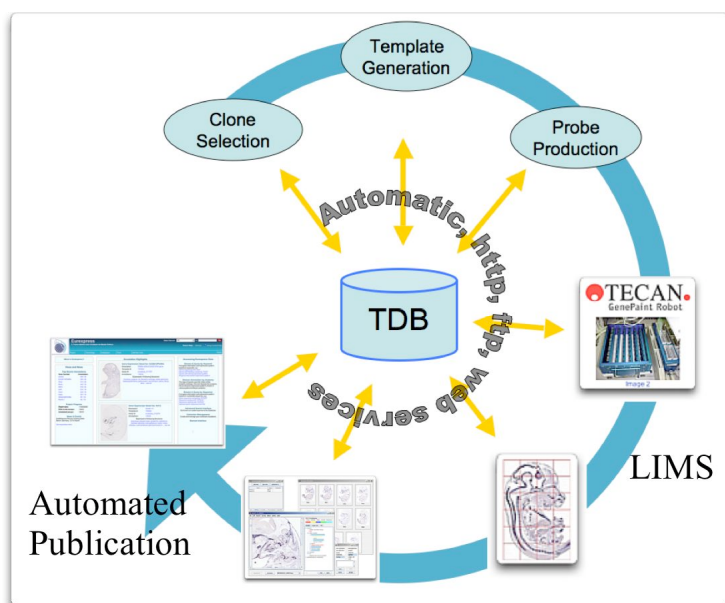

Figure S7

Supplement: Figure S7 — Eurexpress data management architecture. Each process on the outer pipeline is tracked by data exchange with the tracking database (TDB). The yellow arrows represent data flow using protocols as described in the test. (0.66 MB PDF) [file pbio.1000582.s007.pdf]

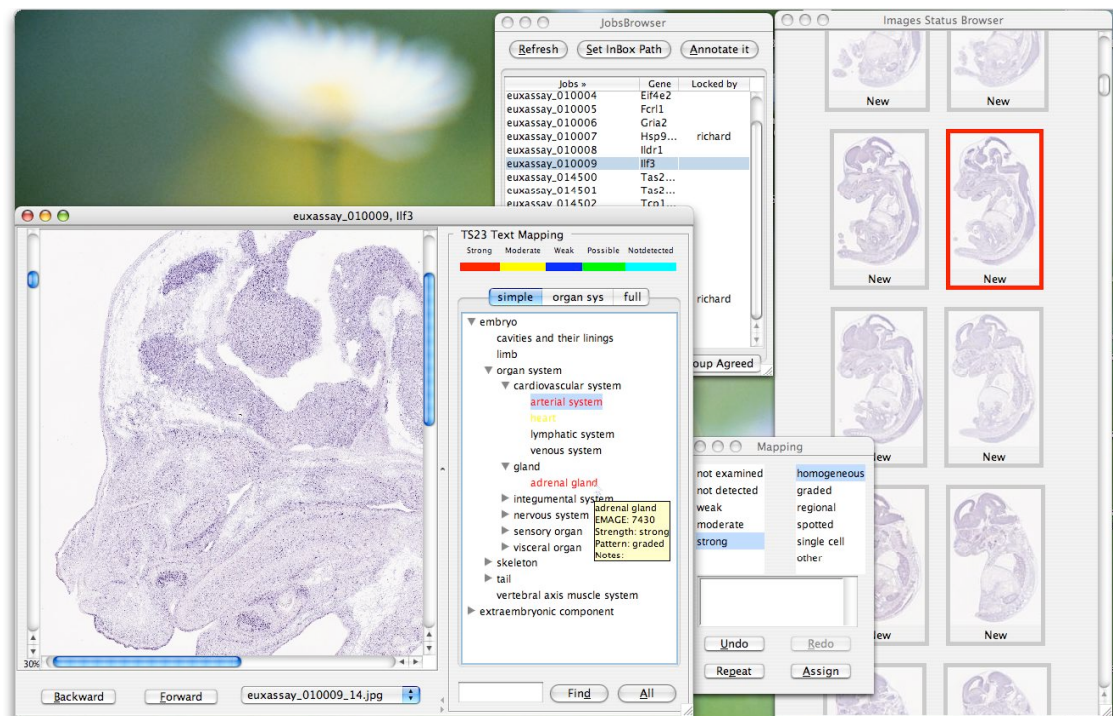

Figure S8

Supplement: Figure S8 — Screen view of the FIATAS annotation interface. The image displayed in the left-hand view can be expanded to full resolution and panned at will. The right-hand side image selector also shows which images are annotated. The upper, partially hidden dialog box shows the current “inbox” and which user is currently annotating which assay, and provides the review and quality control options. The small dialog box lower center provides the annotation options for the selected anatomical terms. (1.42 MB PDF) [file pbio.1000582.s008.pdf]
