# Supplementary material for: A High-Resolution Anatomical Atlas of the Transcriptome in the Mouse Embryo
Source: PLoS Biol. 2011 Jan 18;9(1):e1000582. doi: 10.1371/journal.pbio.1000582 (PMC3022534; doi:10.1371/journal.pbio.1000582)
Supplement: Table S1 — Comparison of independently produced ISH data for the solute carrier superfamily. (0.53 MB PDF) [file pbio.1000582.s009.pdf]

Table S1: Comparison of independently produced ISH-data for the solute carrier superfamily

Family	Gene Symbol (NCBI-link)	set 1 (DB-link)	set 2 (DB-link)	set1 Pattern	set2 Pattern	
high-affinity glutamate and neutral amino acid transporter	Slc1a1	MH2024	euxassay_010393	uwp	reg	
	Slc1a2	MH2136	euxassay_009471	reg	reg	
	Slc1a3	MH1882	euxassay_001899	reg	reg	
	Slc1a4	MH2137	euxassay_000562	reg	reg	
	Slc1a6	MH2138	euxassay_009472	reg	reg	
	Slc1a7	MH2377	euxassay_003213	ubi	ubi	
facilitative GLUT transporter	Slc2a1	MH1862	euxassay_000500	reg	uwp	
	Slc2a2	MH2211	euxassay_005057	reg	reg	
	Slc2a3	MH1883	euxassay_017628	reg	reg	
	Slc2a4	MH1858	euxassay_008805	nd	nd	
	Slc2a5	MH2092	euxassay_017789	nd	nd	
	Slc2a6	MH2145	euxassay_009476	nd	nd	
	Slc2a9	MH2344	euxassay_006278	reg	reg	
	Slc2a10	MH1917	euxassay_014490	ubi	reg	
	Slc2a12	MH3172	euxassay_014526	reg	reg	
	Slc2a13	MH2144	euxassay_002584	reg	reg	
heavy subunits of heteromeric amino acids transporters	Slc3a2	MH2366	euxassay_007752	reg	reg	
bicarbonate transporter	Slc4a5	MH3173	euxassay_013237	reg	reg	
	Slc4a10	MH2878	euxassay_005787	reg	reg	
	Slc4a11	MH1963	euxassay_012145	nd	nd	
sodium glucose cotransporter	Slc5a1	MH2892	euxassay_006141	reg	nd	
	Slc5a2	MH2255	euxassay_008654	reg	nd	
	Slc5a4a	MH2153	euxassay_002846	ubi	nd	
	Slc5a5	MH2845	euxassay_012146	reg	reg	
	Slc5a6	MH1938	euxassay_016837	uwp	uwp	
	Slc5a7	MH2213	euxassay_017136	reg	reg	
	Slc5a8	MH2806	euxassay_016829	reg	nd	
	Slc5a11	MH2829	euxassay_012346	nd	nd	
	Slc5a12	MH2830	euxassay_017111	ubi	nd	
sodium- and chloride-dependent neurotransmitter transporter	Slc6a2	MH1976	euxassay_004595	reg	reg	
	Slc6a5	MH1964	euxassay_012148	reg	reg	
	Slc6a6	MH1975	euxassay_004447	reg	reg	
	Slc6a7	MH2155	euxassay_012672	reg	reg	
	Slc6a8	MH1921	euxassay_008232	ubi	ubi	
	Slc6a9	MH2348	euxassay_003521	reg	reg	
	Slc6a11	MH1911	euxassay_009039	reg	reg	
	Slc6a14	MH1866	euxassay_016455	nd	nd	
	Slc6a15	MH2154	euxassay_012147	reg	reg	
	Slc6a17	MH2893	euxassay_016993	reg	reg	
	Slc6a18	MH2346	euxassay_007790	reg	reg	
	Slc6a19	MH2196	euxassay_012968	nd	nd	
	Slc6a20	MH2347	euxassay_004446	reg	reg	
cationic amino acid transporter/glycoprotein-associated amino acid transporter	Slc7a1	MH2156	euxassay_012248	reg	reg	
	Slc7a2	MH1890	euxassay_012150	reg	reg	
	Slc7a3	MH1965	euxassay_006447	reg	reg	
	Slc7a5	MH1993	euxassay_003902	uwp	nd	
	Slc7a6	MH2159	euxassay_005955	reg	reg	
	Slc7a9	MH2350	euxassay_006096	reg	reg	
	Slc7a10	MH2157	euxassay_006170	reg	reg	
	Slc7a11	MH2074	euxassay_012149	reg	reg	
	Slc7a12	MH2370	euxassay_016352	ubi	nd	
	Slc7a14	MH2313	euxassay_004372	reg	reg	
Na+/Ca2+ exchanger	Slc8a1	MH1892	euxassay_009439	reg	reg	
	Slc8a2	MH2831	euxassay_005720	reg	reg	

Family	Gene Symbol (NCBI-link)	set 1 (DB-link)	set 2 (DB-link)	set1 Pattern	set2 Pattern	
Na+/Ca2+ exchanger	Slc8a3	MH2034	euxassay_016353	reg	reg	
Na+/H+ exchanger	Slc9a1	MH2894	euxassay_001642	uwp	uwp	
	Slc9a2	MH2109	euxassay_012151	nd	nd	
	Slc9a4	MH2895	euxassay_012249	nd	nd	
	Slc9a6	MH1901	euxassay_012153	reg	reg	
	Slc9a7	MH1893	euxassay_012313	nd	nd	
	Slc9a9	MH3147	euxassay_014481	reg	reg	
sodium bile salt cotransporter	Slc10a1	MH2392	euxassay_016659	nd	nd	
	Slc10a4	MH2813	euxassay_010865	reg	reg	
	Slc10a5	MH3145	euxassay_013752	nd	nd	
	Slc10a6	MH2315	euxassay_006446	nd	nd	
	Slc10a7	MH2787	euxassay_017793	reg	reg	
proton coupled metal ion transporter	Slc11a1	MH2133	euxassay_009438	nd	nd	
	Slc11a2	MH2134	euxassay_002307	uwp	uwp	
electroneutral cation-Cl cotransporter	Slc12a1	MH2265	euxassay_005056	reg	uwp	
	Slc12a2	MH1948	euxassay_008837	reg	reg	
	Slc12a3	MH3148	euxassay_001990	nd	nd	
	Slc12a5	MH1950	euxassay_016890	reg	reg	
	Slc12a6	MH1951	euxassay_016520	reg	reg	
	Slc12a7	MH2022	euxassay_009467	reg	reg	
Na+-sulfate/carboxylate cotransporter	Slc13a1	MH2873	euxassay_003034	nd	reg	
	Slc13a2	MH2881	euxassay_011431	reg	nd	
	Slc13a3	MH1953	euxassay_002522	nd	nd	
urea transporter	Slc14a1	MH2207	euxassay_014569	reg	reg	
	Slc14a2	MH2270	euxassay_000491	reg	reg	
proton oligopeptide cotransporter	Slc15a1	MH2400	euxassay_016347	ubi	nd	
	Slc15a2	MH2882	euxassay_006140	reg	reg	
	Slc15a3	MH2023	euxassay_005166	reg	reg	
	Slc15a4	MH2218	euxassay_002984	uwp	uwp	
monocarboxylate transporter	Slc16a4	MH2203	euxassay_001537	reg	reg	
	Slc16a5	MH2397	euxassay_016348	nd	nd	
	Slc16a8	MH1969	euxassay_004301	ubi	nd	
	Slc16a9	MH1983	euxassay_009116	reg	reg	
	Slc16a10	MH1980	euxassay_010493	reg	reg	
	Slc16a11	MH2842	euxassay_009468	ubi	nd	
	Slc16a12	MH2001	euxassay_007957	reg	reg	
vesicular glutamate transporter	Slc17a1	MH2788	euxassay_004444	nd	nd	
	Slc17a2	MH2084	euxassay_006703	nd	nd	
	Slc17a3	MH2340	euxassay_001538	reg	reg	
	Slc17a5	MH1984	euxassay_005553	ubi	nd	
	Slc17a6	MH2208	euxassay_004371	reg	reg	
	Slc17a7	MH2789	euxassay_001627	uwp	uwp	
vesicular amine transporter	Slc18a1	MH2341	euxassay_005750	reg	reg	
	Slc18a3	MH1954	euxassay_009469	reg	reg	
folate/thiamine transporter	Slc19a1	MY266	euxassay_008691	uwp	uwp	
	Slc19a3	MH1956	euxassay_009470	nd	nd	
type-III Na+-phosphate cotransporter	Slc20a1	MH2883	euxassay_009182	reg	reg	
organic anion transporter	Slco1a1	MH2263	euxassay_001539	uwp	uwp	
	Slco1a4	MH2052	euxassay_012154	reg	reg	
	Slco1a6	MH2351	euxassay_001548	reg	reg	
	Slco1c1	MH1994	euxassay_007061	reg	reg	
	Slco2a1	MH2896	euxassay_014301	reg	reg	
	Slco2b1	MH2205	euxassay_012250	reg	reg	
	Slco3a1	MH2053	euxassay_000780	reg	reg	
	Slco5a1	MH1995	euxassay_012155	reg	reg	

Family	Gene Symbol (NCBI-link)	set 1 (DB-link)	set 2 (DB-link)	set1 Pattern	set2 Pattern	
organic anion transporter	Slco6c1	MH2381	euxassay_014527	nd	nd	
organic cation/anion/zwitterion transporter	Slc22a1	MH2085	euxassay_000534	ubi	ubi	
	Slc22a3	MH2044	euxassay_014478	reg	reg	
	Slc22a4	MH2077	euxassay_006930	reg	reg	
	Slc22a5	MH1970	euxassay_007211	uwp	nd	
	Slc22a6	MH1959	euxassay_012001	reg	reg	
	Slc22a9	MH2393	euxassay_008882	nd	nd	
	Slc22a12	MH2106	euxassay_003608	reg	reg	
	Slc22a13	MH3097	euxassay_007886	reg	reg	
	Slc22a15	MH2079	euxassay_000391	uwp	uwp	
	Slc22a16	MH1958	euxassay_016349	nd	nd	
	Slc22a18	MH2884	euxassay_006554	reg	nd	
	Slc22a20	MH2822	euxassay_005954	nd	nd	
Na+-dependent ascorbic acid transporter	Slc23a1	MH3198	euxassay_008672	reg	reg	
	Slc23a2	MH2004	euxassay_019254	reg	reg	
	Slc23a3	MH2086	euxassay_002900	nd	nd	
Na+/(Ca2+-K+) exchanger	Slc24a1	MH2231	euxassay_017761	nd	nd	
	Slc24a2	MH2139	euxassay_014525	nd	nd	
	Slc24a3	MH2005	euxassay_005602	reg	reg	
	Slc24a5	MH1987	euxassay_006990	reg	reg	
mitochondrial carrier	Slc25a1	MH1988	euxassay_007829	reg	reg	
	Slc25a2	MH2859	euxassay_014479	nd	nd	
	Slc25a4	MH2210	euxassay_010636	uwp	uwp	
	Slc25a5	MH2047	euxassay_004504	uwp	uwp	
	Ucp1	MH2096	euxassay_017846	nd	nd	
	Ucp3	MH2333	euxassay_011894	nd	nd	
	Slc25a10	MH2858	euxassay_008673	uwp	uwp	
	Slc25a11	MH2025	euxassay_006233	uwp	uwp	
	Slc25a12	MH2087	euxassay_003824	ubi	ubi	
	Slc25a13	MH2088	euxassay_006704	reg	reg	
	Slc25a14	MH2089	euxassay_004145	nd	nd	
	Slc25a15	MH2026	euxassay_012785	reg	reg	
	Slc25a16	MH2027	euxassay_010736	nd	nd	
	Slc25a17	MH2045	euxassay_004503	uwp	uwp	
	Slc25a19	MH2039	euxassay_010653	ubi	ubi	
	Slc25a20	MH2046	euxassay_007750	uwp	nd	
	Slc25a23	MH2078	euxassay_007865	uwp	uwp	
	Slc25a25	MH2028	euxassay_005554	ubi	nd	
	Slc25a26	MH2141	euxassay_002888	ubi	nd	
	Slc25a27	MH2219	euxassay_014487	reg	reg	
	Slc25a28	MH2091	euxassay_001566	nd	nd	
	Slc25a29	MH2029	euxassay_004416	ubi	nd	
	Slc25a30	MH2063	euxassay_014289	reg	nd	
	Slc25a32	MH2791	euxassay_005621	reg	nd	
	Slc25a33	MH2815	euxassay_016418	nd	nd	
	Slc25a35	MH2317	euxassay_013622	reg	reg	
	Slc25a36	MH2318	euxassay_003666	reg	uwp	
	Slc25a37	MH2319	euxassay_007768	reg	reg	
	Slc25a38	MH2310	euxassay_000811	reg	reg	
	Slc25a39	MH2306	euxassay_000139	reg	uwp	
	Slc25a40	MH2308	euxassay_007962	nd	nd	
	Slc25a43	MH2794	euxassay_013708	nd	nd	
	Slc25a44	MH2309	euxassay_003808	nd	nd	
multifunctional anion exchanger	Slc26a3	MH2342	euxassay_010893	nd	nd	
	Slc26a4	MH2844	euxassay_014488	reg	reg	

Family	Gene Symbol (NCBI-link)	set 1 (DB-link)	set 2 (DB-link)	set1 Pattern	set2 Pattern	
multifunctional anion exchanger	Slc26a5	MH2132	euxassay_016350	uwp	nd	
	Slc26a6	MH2343	euxassay_002740	ubi	nd	
	Slc26a8	MH2378	euxassay_002632	ubi	ubi	
	Slc26a9	MH2401	euxassay_014489	nd	reg	
	Slc26a11	MH1989	euxassay_012246	nd	nd	
fatty acid transporter	Slc27a1	MH2204	euxassay_010364	reg	reg	
	Slc27a2	MH1914	euxassay_011400	reg	reg	
	Slc27a3	MH1915	euxassay_009473	reg	reg	
	Slc27a6	MH1960	euxassay_009474	reg	reg	
Na+-coupled nucleoside transporter	Slc28a1	MH2379	euxassay_006962	nd	reg	
	Slc28a2	MH2191	euxassay_001709	reg	reg	
	Slc28a3	MH2886	euxassay_006705	ubi	reg	
facilitative nucleoside transporter	Slc29a1	MH1916	euxassay_002695	reg	reg	
	Slc29a2	MH2032	euxassay_010127	ubi	nd	
	Slc29a3	MH1990	euxassay_009475	uwp	nd	
	Slc29a4	MH2107	euxassay_005625	uwp	uwp	
zinc efflux transporter	Slc30a1	MH2887	euxassay_004146	reg	reg	
	Slc30a2	MH1961	euxassay_016470	reg	uwp	
	Slc30a3	MH2373	euxassay_009477	reg	reg	
	Slc30a4	MH1991	euxassay_013986	reg	nd	
	Slc30a5	MH1962	euxassay_010448	uwp	uwp	
	Slc30a6	MH1885	euxassay_005975	nd	uwp	
	Slc30a9	MH2069	euxassay_010926	uwp	uwp	
vesicular inhibitory amino acid transporter	Slc32a1	MH2889	euxassay_010420	reg	reg	
acetyle-CoA transporter	Slc33a1	MH2363	euxassay_004169	uwp	nd	
type-II Na+-phosphate cotransporter	Slc34a1	MH2064	euxassay_011507	reg	reg	
	Slc34a2	MH2395	euxassay_017064	ubi	nd	
	Slc34a3	MH2797	euxassay_016351	nd	nd	
nucleoside-sugar transporter	Slc35a1	MH1971	euxassay_006887	reg	reg	
	Slc35a3	MH1972	euxassay_003279	uwp	reg	
	Slc35a4	MH2146	euxassay_004834	uwp	uwp	
	Slc35a5	MH2071	euxassay_002309	nd	nd	
	Slc35b1	MH1929	euxassay_005005	uwp	uwp	
	Slc35b2	MH2072	euxassay_010411	uwp	uwp	
	Slc35b3	MH2192	euxassay_003609	uwp	uwp	
	Slc35c2	MH1930	euxassay_003890	uwp	nd	
	Slc35d1	MH2073	euxassay_016992	reg	reg	
	Slc35d3	MH2042	euxassay_009478	reg	reg	
	Slc35e1	MH2798	euxassay_015962	uwp	uwp	
	Slc35e3	MH2043	euxassay_004678	reg	reg	
	Slc35f1	MH2048	euxassay_008274	reg	reg	
	Slc35f2	MH3177	euxassay_003909	reg	reg	
	Slc35f3	MH2396	euxassay_012247	ubi	nd	
	Slc35f4	MH2799	euxassay_015857	nd	nd	
	Slc35f5	MH2049	euxassay_000120	uwp	uwp	
proton-coupled amino acid transporter	Slc36a1	MH1931	euxassay_012143	reg	reg	
	Slc36a2	MH2380	euxassay_003901	reg	reg	
	Slc36a4	MH2823	euxassay_004078	nd	nd	
sugar-phosphate/phosphate exchanger	Slc37a1	MH2150	euxassay_003753	ubi	nd	
	Slc37a2	MH1886	euxassay_016891	nd	nd	
	Slc37a3	MH1932	euxassay_017080	uwp	nd	
	Slc37a4	MH2875	euxassay_005966	reg	uwp	
System A and N, sodium-coupled neutral amino acid transporter	Slc38a2	MH1895	euxassay_008760	reg	reg	
	Slc38a3	MH2193	euxassay_004113	reg	reg	
	Slc38a4	MH2364	euxassay_004567	nd	reg	

Family	Gene Symbol (NCBI-link)	set 1 (DB-link)	set 2 (DB-link)	set1 Pattern	set2 Pattern
System A and N, sodium-coupled neutral amino acid transporter	Slc38a5	MH2051	euxassay_002353	reg	reg
	Slc38a6	MH2800	euxassay_016081	reg	nd
	Slc38a9	MH3154	euxassay_005652	nd	nd
	Slc38a10	MH3099	euxassay_004151	uwp	uwp
	Slc38a11	MH3154	euxassay_013418	nd	nd
metal ion transporter	Slc39a1	MH3187	euxassay_004867	uwp	uwp
	Slc39a4	MH2008	euxassay_003030	uwp	reg
	Slc39a5	MH1934	euxassay_009125	reg	ubi
	Slc39a6	MH1935	euxassay_001445	uwp	uwp
	Slc39a7	MH1918	euxassay_002762	uwp	uwp
	Slc39a8	MH2409	euxassay_011277	reg	reg
	Slc39a9	MH1919	euxassay_014261	uwp	nd
	Slc39a10	MH2006	euxassay_008838	reg	reg
basolateral ion transporter	Slc40a1	MH1936	euxassay_003910	reg	reg
MgtE-like magnesium transporter	Slc41a1	MH2877	euxassay_012144	reg	reg
	Slc41a2	MH1887	euxassay_009324	nd	nd
	Slc41a3	MH3183	euxassay_006674	reg	reg
Rh ammonium transporter (pending)	Rhag	MH2082	euxassay_009664	reg	reg
	Rhcg	MH2821	euxassay_007842	reg	reg
Na+-independent, syste-L-like amino acid transporter	Slc43a3	MH2403	euxassay_005875	reg	reg
Choline-like transporter family	Slc44a1	MH2817	euxassay_012718	reg	nd
	Slc44a2	MH2824	euxassay_003668	reg	reg
	Slc44a3	MH2818	euxassay_002530	reg	reg
	Slc44a4	MH2801	euxassay_007818	reg	reg
Putative sugar transporter family	Slc45a1	MH2803	euxassay_000403	nd	nd
	Slc45a2	MH2825	euxassay_013778	nd	nd
Heme transporter family	Slc46a1	MH2808	euxassay_001608	nd	nd
	Slc46a2	MH2826	euxassay_012435	reg	reg
	Slc46a3	MH2809	euxassay_000957	nd	nd
Multidrug and toxin extrusion	Slc47a2	MH2810	euxassay_013290	nd	nd

set 1: data from www.genepaint.org (Geffers and Eichele, unpublished data)
set 2: data from www.eurexpress.org
abbreviations: reg = regional; nd = not detected; uwp = ubiquitous with pattern; ubi = ubiquitous
color code: consistent (green); nd and weak ubi or weak uwp (yellow); inconsistent (red)

The majority of cases are consistent between sets 1 and 2 (green, n = 195, 78%). ISH often cannot distinguish between nd and weak ubi or weak uwp, and 25 (10%) cases fell into this class and are marked in yellow. 28 (12%) cases were inconsistent between sets 1 and 2 and are marked in red. Thus of the 122 reg expression patterns of set 2, 113 are also reg in set 1, while 9 are either uwp, ubi or nd. Hence in the most informative category of regional expression, the two sets agree more than 90%. A similar agreement is seen with the uwp of sets 1 and 2. As can be expected the agreement for nd is somewhat lower with an agreement of 52 out of 86 patterns.
