# Supplementary material for: A High-Resolution Anatomical Atlas of the Transcriptome in the Mouse Embryo
Source: PLoS Biol. 2011 Jan 18;9(1):e1000582. doi: 10.1371/journal.pbio.1000582 (PMC3022534; doi:10.1371/journal.pbio.1000582)
Supplement: Table S2 — Validation of Eurexpress data against published data. (0.21 MB DOC) [file pbio.1000582.s010.pdf]

Table S2: Comparison of EURExpress data against published expression data

Template ID	*RefSeq accession*	*Gene symbol*	*Concordance*	*Reference*
T1569	NM_023906	Asb3	NP	
T4116	NM_133733	9030425E11Rik	NP	
T5339	NM_133190	Cacng8	NP	
T7613	NM_133750	3110048E14Rik	NP	
T8641	NM_013581	Coq1	NP	
T8887	NM_008151	Gpr12	CONCORDANCE	Ignatov A, Lintzel J, Hermans-Borgmeyer I, Kreienkamp HJ, Joost P, Thomsen S, Methner A, Schaller HC. Role of the G-protein-coupled receptor GPR12 as high-affinity receptor for sphingosylphosphorylcholine and its expression and function in brain development. J Neurosci 2003 Feb 1,23(3):907-14
T7122	NM_016736	6330412F12Rik (Nub1)	NP	
T35200	NM_177383	Gpr21	NP	
T2778	NM_010903	Nfe2l3	NP	
T35433	NM_175647	Dmrta1	NP	
T35847	XR_005251	A930024E05Rik	NP	
T36070	XM_355752	Dgki	NP	
T9316	NM_027135	Sec24d	NP	
T31018	NM_021499	Wdr8	NP	
T31985	NM_027260	Vrk2	NP	
T30457	NM_008992	Abcd4	NP	
T37784	NM_080795	Lnx2	CONCORDANCE	Rice DS, Northcutt GM, Kurschner C. "The Lnx family proteins function as molecular scaffolds for Numb family proteins." Mol Cell Neurosci 2001 Nov,18(5):525-40
T40492	AK018398	8430408J09Rik	NP	
T45541	NM_001014390	Dyrk2	NP	
T70151	MIMAT0003491	mmu-miR-701	NP	
T36964	NM_172516	Ripk5	NP	
T3840	NM_018884	Pdzrn3	NP	
T2359	NM_023850	Chst1	NO CONCORDANCE	van der Zwaag B, Burbach JP, Scharfe C, Oefner PJ, Brunner HG, Padberg GW, van Bokhoven H. "Identifying new candidate genes for hereditary facial paresis on chromosome 3q21-q22 by RNA in situ hybridization in mouse." Genomics 2005 Jul,86(1):55-67
T4098	NM_172310	Tarsl2	NP	
T7610	NM_178891	Hrmt1l6	NP	
T35194	NM_181543	Gpr151	CONCORDANCE	Ignatov A, Hermans-Borgmeyer I, Schaller HC. "Cloning and characterization of a novel G-protein-coupled receptor with homology to galanin receptors." Neuropharmacology 2004 Jun,46(8):1114-20
T36957	NM_021892	Rfrp	NP	
T38159	NM_177892	A230097C02	NP	
T30773	NM_009383	Tial1	NP	
T8662	NM_178625	2700094F01Rik	NP	
T35706	NM_027760	Rassf8	NP	
T36162	NM_153105	Cldn19	NP	
T37488	NM_175651	9630008K15Rik	NP	
T36121	NM_175631	Cbln4	NP	
T31417	NM_029945	Smpd4	NP	
T2314	NM_025897	1500003O22Rik	NP	
T38213	NM_028065	Tnrc5	NP	
T40280	AK077915	Mipol1	NP	
T45629	XM_973549	4930589M24Rik	NP	
T45132	XM_890447	Mllt4	NP	
T6536	NM_028184	Oraov1	NP	
T35739	NM_027519	6330406I15Rik	NP	
T36476	NM_015820	Hs6st3	NP	
T37027	NM_009200	Slc1a6	NP	
T31051	NM_029933	Bcl9	NP	
T63352	NM_207237	Man1c1	NP	
T63289	NM_198678	Sh3md2	NP	
T35109	NM_009766	Brs3	NP	
T9940	NM_026925	Pnlip	NP	
T2618	NM_022408	Es2el	CONCORDANCE	Lindsay EA, Harvey EL, Scambler PJ, Baldini A. "ES2, a gene deleted in DiGeorge syndrome, encodes a nuclear protein and is expressed during early mouse development, where it shares an expression domain with a Goosecoid-like gene." Hum Mol Genet 1998 Apr,7(4):629-35
T36864	NM_001001320	Tbx10	NP	
T38784	NM_008022	Foxd4	NP	
T40239	XM_989876	4631416L12Rik	NP	
T3799	NM_023438	Gm644	NP	
T5619	NM_026012	Nradd	NP	
T35259	NM_013582	Lhcgr	NP	
T35827	NM_177358	A630033E08Rik	NP	
T35828	NM_177255	A630052C17Rik	NP	
T36821	XM_486090	Nkx6-3	PARTIAL CONCORDANCE	Alanentalo T, Chatonnet F, Karlen M, Sulniute R, Ericson J, Andersson E, Ahlgren U. "Cloning and analysis of Nkx6.3 during CNS and gastrointestinal development." Gene Expr Patterns 2006 Jan,6(2):162-70
				Pedersen JK, Nelson SB, Jorgensen MC, Henseleit KD, Fujitani Y, Wright CV, Sander M, Serup P. "Endodermal expression of Nkx6 genes depends differentially on Pdx1." Dev Biol 2005 Dec 15,288(2):487-501
T36197	NM_170597	Creg2	NP	
T31600	NM_181315	Car5b	PARTIAL CONCORDANCE	Cankaya M, Hernandez AM, Ciftci M, Beydemir S, Ozdemir H, Budak H, Gulcin I, Comakli V, Emircupani T, Ekinci D, Kuzu M, Jiang Q, Eichele G, Kufrevioglu OI. "An analysis of expression patterns of genes encoding proteins with catalytic activities." BMC Genomics 2007,8():232
T31319	NM_028248	2610301K12Rik	NP	
T38668	NM_010586	Itpr2	NP	
T5360	NM_008265	Hoxa4	NP	
T5313	NM_010454	Hoxa6	NP	
T3054	NM_144544	2210407C18Rik	NP	
T36034	NM_181422	Pkd2l1	NP	
T36325	NM_139293	Ece2	NP	
T35172	NM_177330	Ghsr	NP	
T35853	NM_175352	AA407659	NP	
T37251	NM_027401	1700010C24Rik	NP	
T30543	NM_016663	Syt3	PARTIAL CONCORDANCE	Visel A, Thaller C, Eichele G. "GenePaint.org: an atlas of gene expression patterns in the mouse embryo." Nucleic Acids Res 2004 Jan 1,32(Database issue):D552-6S,
T38441	NM_008226	Hcn2	NP	
T37397	XM_916223	4931429I11Rik	NP	
T31587	NM_026013	Tmem77	NP	
T38192	NM_138948	Cabp7	NP	
T40298	AK015488	4930459I23Rik	NP	
T39753	AK083519	D030040B21	NP	
T45276	NM_145579	P42pop	NP	
T70096	MIMAT0005292	mmu-miR-582-3p	NP	
T70406	MIMAT0000567	mmu-miR-329	NP	
T4202	NM_028207	Dusp3	NP	
T632	NM_029031	Carkl	NP	
T4705	NM_019443	Ndufa1	NP	

T5592	NM_176848	Fbxo2	PARTIAL CONCORDANCE	Hagens O, Minina E, Schweiger S, Ropers HH, Kalscheuer V. "Characterization of FBX25, encoding a novel brain-expressed F-box protein." Biochim Biophys Acta 2006 Jan,1760(1):110-8
T35378	NM_175198	1700058C01Rik	NP	
T35883	NM_009620	Adam4	NP	
T36751	NM_053144	Pcdhb19	NP	
T36312	XM_917279	Layn	NP	
T35839	NM_177045	Cc2d1b	NP	
T36635	NM_001033339	Mmp25	NP	
T37022	NM_011390	Slc12a7	NP	
T30923	NM_138650	Dgkg	CONCORDANCE	Visel A, Thaller C, Eichele G. "GenePaint.org: an atlas of gene expression patterns in the mouse embryo." Nucleic Acids Res 2004 Jan 1,32(Database issue):D552-6
T32139	NM_133914	Rasa4	NP	
T37566	NM_181728	Art3	NP	
T39008	NM_178651	Slc30a9	NP	
T39034	NM_021332	Glp1r	NP	
T37560	NM_177781	Trpa1	NP	
T30023	NM_009831	Ccng1	NP	
T37051	NM_172841	Slco5a1	NP	
T37651	NM_029879	D13Bwg1146e	NP	
T31259	NM_145497	BC016495	NP	
T37063	NM_198214	Snph	NP	
T40474	AK083647	D030059C06Rik	NP	
T40506	AK004446	1190002E22Rik	NP	
T35036	XM_134539	2310031A18Rik	NP	
T3272	NM_012026	Rgnef	NP	
T8010	NM_008213	Hand1	PARTIAL CONCORDANCE	Cserjesi P; Brown D; Lyons GE; Olson EN, "Expression of the novel basic helix-loop-helix gene eHAND in neural crest derivatives and extraembryonic membranes during mouse development." Dev Biol 1995 Aug;170(2):664-78
T3514	NM_172451	Galnt6	NP	
T35962	NM_009726	Atp7a	NP	
T1499	NM_177226	Zfp629	NP	
T3628	NM_144817	Camk1g	NP	
T3678	NM_134022	6330403K07Rik	NP	
T1199	NM_019806	Vapb	NP	
T5536	NM_023397	1810034K20Rik	NP	
T3282	NM_026552	Arpc4	NP	
T3389	NM_025947	Dncl2a	NP	
T3378	NM_177262	Pkn1	NP	
T7848	NM_178358	Lhfpl1	NP	
T3053	NM_009894	Cideb	NP	
T35608	NM_026622	3110057O12Rik	NP	
T36442	NM_010305	Gnai1	NP	
T45437	NM_199365	5330438I03Rik	NP	
T70297	MIMAT0000215	mmu-miR-186	NP	
T992	NM_026009	2610204L23Rik	NP	
T4565	NM_025884	Ccdc16	NP	
T4570	NM_027231	Polr2f	NP	
T1153	NM_126166	Tlr3	NP	
T6034	NM_025297	Nrbf1	NP	
T5194	NM_025920	Thap4	NP	
T3931	NM_019870	Ard1	NP	
T994	NM_023167	Mrpl4	NP	
T1053	NM_007991	Fbl	NP	
T4266	NM_181410	Gtf2h3	NP	
T4653	NM_146238	BC023488	NP	
T4654	NM_025644	Exosc1	NP	
T4649	NM_025298	Polr3e	NP	
T4839	NM_020483	Hcngp	NP	
T4699	NM_026421	2310057D15Rik	NP	
T4918	NM_172049	Tmem18	NP	
T6193	NM_144829	2310044P18Rik	NP	
T6824	NM_133756	Xab1	NP	
T6826	NM_025567	Cyc1	NP	
T6808	NM_145386	BC005655	NP	
T8575	NM_029418	9130401M01Rik	NP	
T8298	NM_053113	Ear11	NP	
T35764	NM_177136	9030227G01Rik	NP	
T7090	NM_053168	Trim11	NO CONCORDANCE	Hong SJ, Chae H, Lardaro T, Hong S, Kim KS. "Trim11 increases expression of dopamine beta-hydroxylase gene by interacting with Phox2b." Biochem Biophys Res Commun 2008 Apr 11,368(3):650-5
T7340	NM_019910	Dcpp	NP	
T8417	NM_026744	Mrpl53	NP	
T36466	NM_010411	Hdac3	NP	
T31763	NM_023197	2310008H09Rik	NP	
T36482	NM_010489	Hyal2	NP	
T30449	NM_028604	2410075D05Rik	NP	
T45583	NM_028274	Exosc6	NP	
T39150	NM_053170	Trim33	NP	
T38409	XM_109726	Thrap1	NP	
T39614	Y15800	Gprk6	NP	
T40565	NM_001040026	Sco1	NP	
T40143	AK013632	LOC434446	NP	
T40477	AK156793	Dnajc11	NP	
T63207	NM_178640	B3galnt2	NP	
T63369	NM_207239	Gtf3c1	NP	
T35071	XM_135805	Wdr44	NP	
T7055	NM_146236	Tceal1	NP	
T37506	NM_175511	A130092J06Rik	NP	
T37713	NM_027402	Fndc5	NP	
T39931	AK044039	LOC432809	NP	
T2189	NM_029320	D14Ertd581e	NP	
T30549	NM_178711	Plscr4	NP	
T30424	NM_148942	Serpinb6c	NP	
T3497	NM_001012392	U46068	CONCORDANCE	Hou J, Yashiro K, Okazaki Y, Saijoh Y, Hayashizaki Y, Hamada H. "Identification of a novel left-right asymmetrically expressed gene in the mouse belonging to the BPI/PLUNC superfamily." Dev Dyn 2004 Mar,229(2):373-9
T5284	NM_145469	9330161F08Rik	NP	
T35959	NM_009724	Atp4b	NP	
T37359	NM_030181	Vsig1	NP	
T37452	NM_025684	5730521E12Rik	CONCORDANCE	Li X, Udager AM, Hu C, Qiao XT, Richards N, Gumucio DL. "Dynamic patterning at the pylorus: Formation of an epithelial intestine-stomach boundary in late fetal life." Dev Dyn 2009 Oct 29,238(12):3205-3217
T37039	NM_053248	Slc5a5	NP	
T31264	NM_027299	Degs2	NP	
T37839	NM_001033366	Dpcr1	NP	
T6200	NM_010893	Neu1	NP	
T8444	NM_138646	Hps4	NP	
T7375	NM_178308	Abpg	NP	
T4828	NM_145932	D630035O19Rik	NP	
T35459	NM_013920	Hnf4g	NP	
T36820	XM_134330	Isx	NP	

T36216	XM_130038	Cubn	NP	
T36195	XM_129769	Cps1	NP	
T31175	NM_010739	Muc13	NP	
T31255	NM_144807	Chpt1	NP	
T38238	NM_027339	2210415F13Rik	NP	
T37860	NM_001011873	Xkr9	NP	
T39400	XR_003553	LOC671753	NP	
T2987	NM_027307	Golph2	NP	
T2287	NM_028189	B3gnt3	NP	
T32098	NM_007769	Dmbt1	NP	
T37786	XM_923886	Gm53	NP	
T1660	NM_011280	Trim10	NP	
T452	NM_146173	1300010A20Rik	NP	
T3791	NM_153567	5033405K12Rik	NP	
T800	NM_172759	Ces5	NP	
T855	NM_009127	Scd1	NP	
T518	NM_008934	Proc	NP	
T887	NM_010168	F2	NP	
T867	NM_133977	Trf	CONCORDANCE	Zhou Q, Law AC, Rajagopal J, Anderson WJ, Gray PA, Melton DA. "A multipotent progenitor domain guides pancreatic organogenesis." Dev Cell 2007 Jul,13(1):103-14
T1441	NM_008777	Pah	NP	
T113	NM_011270	Rhced	NP	
T928	NM_023383	Aadac	NP	
T882	NM_008124	Gjb1	NP	
T1032	NM_008878	Serpinf2	NP	
T3836	NM_138678	Butr1	NP	
T1017	NM_013782	Ptdss2	NP	
T3940	NM_019687	Slc22a4	NP	
T4696	NM_007931	Endog	NP	
T2929	NM_020583	Isg20	NP	
T2794	NM_008198	H2-Bf	NP	
T4763	NM_172577	Slc25a21	NP	
T4783	NM_010006	Cyp2d9	NP	
T4794	NM_146148	C8a	NP	
T4813	NM_174870	Slc26a1	NP	
T4015	NM_029269	Spp2	NP	
T1352	NM_174846	6230410P16Rik	NP	
T3113	NM_008385	Inpp5b	NP	
T4399	NM_016956	Hbb-b2	NP	
T4928	NM_008280	Lipc	NP	
T4954	NM_009778	C3	NP	
T4955	NM_007376	Pzp	CONCORDANCE	Lorent K, Overbergh L, Delabie J, Van Leuven F, Van den Berghe H. "Distribution of mRNA coding for alpha-2-macroglobulin, the murinoglobulins, the alpha-2-macroglobulin receptor and the alpha-2-macroglobulin receptor associated protein during mouse embryogenesis and in adult tissues." Differentiation 1994 Feb,55(3):213-23
T705	NM_013474	*Apoa2*	CONCORDANCE	Visel A, Thaller C, Eichele G. "GenePaint.org: an atlas of gene expression patterns in the mouse embryo." Nucleic Acids Res 2004 Jan 1,32(Database issue):D552-6
T647	NM_009695	Apoc2	NP	
T715	NM_023530	Pla2g12b	NP	
T5324	NM_001033981	LOC544763	NP	
T5508	NM_025988	Acbd4	NP	
T6010	NM_013551	Hmbs	NP	
T6020	NM_010196	Fga	NP	
T1930	NM_145824	Ranbp10	NP	
T914	NM_013475	Apoh	NP	
T201	NM_153143	Kctd11	NP	
T1576	NM_026157	Papd1	NP	
T4729	NM_017366	Acadvl	NP	
T4876	NM_144916	BC014685	NP	
T4914	NM_133997	Apof	NP	
T4916	NM_144869	BC021614	NP	
T4901	NM_021489	F12	NP	
T4900	NM_018816	Apom	NP	
T918	NM_009060	Rgn	CONCORDANCE	Keith Ho HC, McGrath KE, Brodbeck KC, Palis J, Schick BP. "Serglycin proteoglycan synthesis in the murine uterine decidua and early embryo." Biol Reprod 2001 Jun,64(6):1667-76
T6813	NM_011787	Amfr	NP	
T6738	NM_011804	Creg1	NP	
T7396	NM_028066	F11	NP	
T6633	NM_144533	Nmnat3	NP	
T7241	NM_008341	Igfbp1	NP	
T4958	NM_019447	Hgfac	NP	
T4997	NM_144805	Tmem40	NO CONCORDANCE	Thut CJ, Rountree RB, Hwa M, Kingsley DM. "A large-scale in situ screen provides molecular evidence for the induction of eye anterior segment structures by the developing lens." Dev Biol 2001 Mar 1,231(1):63-76
T4937	NM_080844	Serpinc1	NP	
T4953	NM_130452	Bbox1	NP	
T7876	NM_013711	Txnrd2	NP	
T6096	NM_144923	Blvrb	NP	
T7438	NM_017371	Hpxn	NP	
T7460	NM_022884	Bhmt2	NP	
T7462	NM_008439	Khk	NP	
T8819	NM_172406	Als2cr3	NP	
T8827	NM_013848	Ermap	CONCORDANCE	Ye TZ, Gordon CT, Lai YH, Fujiwara Y, Peters LL, Perkins AC, Chui DH. "Ermap, a gene coding for a novel erythroid specific adhesion/receptor membrane protein." Gene 2000 Jan 25,242(1-2):337-45
T8327	NM_011414	Slpi	NP	
T9595	NM_207204	4930519N13Rik	NP	
T6101	NM_009775	Bzrp	CONCORDANCE	Visel A, Thaller C, Eichele G. "GenePaint.org: an atlas of gene expression patterns in the mouse embryo." Nucleic Acids Res 2004 Jan 1,32(Database issue):D552-6
T7233	NM_009690	Cd5l	NP	
T2656	NM_013484	C2	NP	
T9775	NM_053149	Hemgn	CONCORDANCE	Yang LV, Nicholson RH, Kaplan J, Galy A, Li L. "Hemogen is a novel nuclear factor specifically expressed in mouse hematopoietic development and its human homologue EDAG maps to chromosome 9q22, a region containing breakpoints of hematological neoplasms." Mech Dev 2001 Jun,104(1-2):105-11
T10041	NM_008223	Serpind1	NP	
T9535	NM_009479	Uros	NP	
T103	NM_145365	Creb3l3	PARTIAL CONCORDANCE	Li X, Udager AM, Hu C, Qiao XT, Richards N, Gumucio DL. "Dynamic patterning at the pylorus: Formation of an epithelial intestine-stomach boundary in late fetal life." Dev Dyn 2009 Oct 29,238(12):3205-3217
T544	NM_010799	Minpp1	CONCORDANCE	Chi H, Yang X, Kingsley PD, O'Keefe RJ, Puzas JE, Rosier RN, Shears SB, Reynolds PR. "Targeted deletion of Minpp1 provides new insight into the activity of multiple inositol polyphosphate phosphatase in vivo." Mol Cell Biol 2000 Sep,20(17):6496-507
T659	NM_012053	Rpl8	NP	
T572	NM_031164	F13b	NP	
T663	NM_008406	Itih1	NP	
T6652	NM_026635	5730536A07Rik	NP	

T8083	NM_008114	Gfi1b	NP	
T7377	NM_011822	Pigq	NP	
T7388	NM_008277	Hpd	PARTIAL CONCORDANCE	Ruchon AF, Marcinkiewicz M, Siegfried G, Tenenhouse HS, DesGroseillers L, Crine P, Boileau G. "Pex mRNA is localized in developing mouse osteoblasts and odontoblasts." J Histochem Cytochem 1998 Apr,46(4):459-68
T7065	NM_027868	Slc41a3	NP	
T6647	NM_008522	Ltf	NP	
T6734	NM_019687	Slc22a4	NP	
T4533	NM_013547	Hgd	PARTIAL CONCORDANCE	Visel A, Thaller C, Eichele G. "GenePaint.org: an atlas of gene expression patterns in the mouse embryo." Nucleic Acids Res 2004 Jan 1,32(Database issue):D552-6
T50012	NM_010509	Ifnar2	PARTIAL CONCORDANCE	Reymond A, Marigo V, Yaylaoglu MB, Leoni A, Ucla C, Scamuffa N, Caccioppoli C, Dermitzakis ET, Lyle R, Banfi S, Eichele G, Antonarakis SE, Ballabio A. "Human chromosome 21 gene expression atlas in the mouse." Nature 2002 Dec 5,420(6915):582-6
T3028	NM_007563	Bpgm	NP	
T35297	NM_011465	Spna1	NP	
T35306	NM_013838	Trpc6	NP	
T35925	NM_007440	Alox12	NO CONCORDANCE	Sun D, McDonnell M, Chen XS, Lakkis MM, Li H, Isaacs SN, Elsea SH, Patel PI, Funk CD. Human 12(R)-lipoxygenase and the mouse ortholog. Molecular cloning, expression, and gene chromosomal assignment. J Biol Chem 1998 Dec 11,273(50):33540-7
T36774	NM_008823	Cfp	NP	
T1370	NM_007940	Ephx2	NP	
T2632	NM_134249	Timd2	NP	
T36344	NM_013513	Epb4.2	PARTIAL CONCORDANCE	Zhu L, Kahwash SB, Chang LS. "Developmental expression of mouse erythrocyte protein 4.2 mRNA: evidence for specific expression in erythroid cells." B
T36357	NM_007976	F5	NP	
T36359	NM_176935	F730015K02Rik	NP	
T35159	NM_007975	F2rl3	NP	
T35249	NM_010605	Kcnj5	NP	
T35250	NM_001033525	Kcnk6	NP	
T35158	NM_010170	F2rl2	NP	
T1306	NM_008877	Plg	NP	
T1305	NM_026673	0610008C08Rik	NP	
T36422	NM_008129	Gclm	PARTIAL CONCORDANCE	Diaz D, Krejsa CM, White CC, Charleston JS, Kavanagh TJ. "Effect of methylmercury on glutamate-cysteine ligase expression in the placenta and yolk sac during mouse development." Reprod Toxicol 2004 Nov,19(1):117-29
T36685	NM_008694	Ngp	NP	
T36891	NM_008920	Prg2	NP	
T36894	NM_016781	Prkag1	NP	
T36220	NM_007807	Cybb	NP	
T36962	NM_011269	Rhag	NO CONCORDANCE	Sansom SN, Griffiths DS, Faedo A, Kleinjan DJ, Ruan Y, Smith J, van Heyningen V, Rubenstein JL, Livesey FJ. "The level of the transcription factor Pax6 is essential for controlling the balance between neural stem cell self-renewal and neurogenesis." PLoS Genet 2009 Jun,5(6):e1000511
T36371	NM_030236	Fbxo34	NP	
T37562	XM_139463	Apol2	NP	
T30515	NM_010815	Grap2	NP	
T30513	NM_026464	Wdr55	NP	
T30262	NM_009146	Frrs1	NP	
T30528	NM_011461	Spic	NP	
T30861	NM_008525	Alad	NP	
T31177	NM_177564	BC022224	NP	
T31242	NM_181849	Fgb	NP	
T31238	NM_010531	Il18bp	NP	
T36214	NM_007800	Ctsg	NP	
T36584	NM_008491	Lcn2	NP	
T36625	NM_008572	Mcpt8	NP	
T37518	NM_177115	37682	NP	
T37548	NM_026212	Agpat2	NO CONCORDANCE	Visel A, Thaller C, Eichele G. "GenePaint.org: an atlas of gene expression patterns in the mouse embryo." Nucleic Acids Res 2004 Jan 1,32(Database issue):D552-6
T30120	NM_016751	Clec4f	NP	
T30968	NM_023737	Ehhadh	NP	
T31016	NM_027062	C8g	NP	
T31697	NM_178713	Aldh8a1	CONCORDANCE	Romand R, Kondo T, Fraulob V, Petkovich M, Dolle P, Hashino E. "Dynamic expression of retinoic acid-synthesizing and -metabolizing enzymes in the developing mouse inner ear." J Comp Neurol 2006 Jun 10,496(5):643-54
T37082	NM_138673	Stab2	NP	
T36464	NM_175000	Hbq1	NP	
T37646	NM_023785	Cxcl7	NP	
T31265	NM_178936	Tmem56	NP	
T38059	NM_178931	Tnfrsf14	NP	
T31407	NM_008254	Hmgcl	NP	
T38067	NM_027763	Treml1	NP	
T38095	NM_023500	Xkh	NP	
T31621	NM_181420	BC032265	NP	
T39081	NM_133246	Ms4a3	NP	
T39334	XM_914077	LOC545486	NP	
T39919	NM_001033488	Gm1964	NP	
T37793	XM_001002626	Gm323	NP	
T31656	NM_019949	Ube2l6	NP	
T39636	NM_013730	Slamf1	NP	
T37994	NM_173051	Serpinb1c	NP	
T63033	NM_010575	Itga2b	NP	
T45073	NM_010190	Fcnb	NP	
T45189	S66283	Spnb1	NP	
T70034	MIMAT0004870	mmu-miR-453	NP:microRNA	
T6962	NM_133993	Pwp1	NP	
T6275	NM_080434	Apoa5	NP	
T4913	NM_054094	Bucs1	NP	
T6471	NM_019734	Asah1	NP	
T6515	NM_026434	Rbm18	NP	
T6164	NM_145442	Mbip	NO CONCORDANCE	Tucker ES, Segall S, Gopalakrishna D, Wu Y, Vernon M, Polleux F, Lamantia ASJMolecular specification and patterning of progenitor cells in the lateral and medial ganglionic eminences. J. Neurosci. 2008 Sep 17,28(38):9504-18.
T2760	NM_172372	Wdr45	NP	
T8173	NM_026812	1110033O09Rik	NP	
T7968	NM_028798	2300002G24Rik	NP	
T8216	NM_008403	Itgb1bp1	NP	
T9979	NM_011359	Sftpc	NP	
T48	NM_011247	Rbbp6	NP	
T36440	XM_977462	Gm632	NP	
T36888	NM_138605	Ppp1r3f	NP	
T36222	NM_007814	Cyp2b19	NP	

T37377	XM_485433	4930544O15Rik	NP	
T37174	NM_009417	Tpo	NO CONCORDANCE	Meunier D, Aubin J, Jeannotte L. "Perturbed thyroid morphology and transient hypothyroidism symptoms in Hoxa5 mutant mice." Dev Dyn 2003 Jul,227(3):367-78
T37797	NM_001033246	Gm172	NP	
T36519	NM_172161	Irak2	NP	
T39769	AK034327	9330178D15	NP	
T472	NM_026210	1810036I24Rik	NP	
T1670	NM_013593	Mb	NP	
T2081	NM_146242	Lrrc10	NP	
T1653	NM_008726	Nppb	CONCORDANCE	Cameron VA, Aitken GD, Ellmers LJ, Kennedy MA, Espiner EA. "The sites of gene expression of atrial, brain, and C-type natriuretic peptides in mouse fetal development: temporal changes in embryos and placenta." Endocrinology 1996 Mar,137(3):817-24
T5933	NM_008590	Mest	NP	
T7325	NM_008424	Kcne1	NP	
T4941	NM_026977	1810031K17Rik	NP	
T4371	NM_145367	Txndc5	NP	
T4400	NM_011366	Sh3d4	NP	
T6397	NM_013483	Btn1a1	NP	
T7934	NM_011658	Twist1	PARTIAL CONCORDANCE	Bourgeois P, Bolcato-Bellemin AL, Danse JM, Bloch-Zupan A, Yoshiba K, Stoetzel C, Perrin-Schmitt F. "The variable expressivity and incomplete penetrance of the twist-null heterozygous mouse phenotype resemble those of human Saethre-Chotzen syndrome." Hum Mol Genet 1998 Jun,7(6):945-57
T9824	NM_023129	Pln	NP	
T4377	NM_153805	Pkn3	NP	
T10016	NM_008725	Nppa	CONCORDANCE	Zeller R, Bloch KD, Williams BS, Arceci RJ, Seidman CE. "Localized expression of the atrial natriuretic factor gene during cardiac embryogenesis." Genes Dev 1987 Sep,1(7):693-8
T10015	NM_013868	Hspb7	NP	
T9977	NM_010861	Myl2	CONCORDANCE	Chuva de Sousa Lopes SM, Hassink RJ, Feijen A, van Rooijen MA, Doevendans PA, Tertoolen L, Brutel de la Riviere A, Mummery CL. "Patterning the heart, a template for human cardiomyocyte development." Dev Dyn 2006 Apr 28,235(7):1994-2002
T9973	NM_009406	Tnni3	CONCORDANCE	Mommersteeg MT, Brown NA, Prall OW, de Gier-de Vries C, Harvey RP, Moorman AF, Christoffels VM. "Pitx2c and Nkx2-5 are required for the formation and identity of the pulmonary myocardium." Circ Res 2007 Oct 26,101(9):902-9
T9980	NM_022879	Myl7	CONCORDANCE	Kastner P, Messaddeq N, Mark M, Wendling O, Grondona JM, Ward S, Ghyselinck N, Chambon P. Vitamin A deficiency and mutations of RXRalpha, RXRbeta and RARalpha lead to early differentiation of embryonic ventricular cardiomyocytes., Development 1997 Dec,124(23):4749-58
T35348	NM_026831	Mybphl	NP	
T45	NM_028990	8430437G11Rik	NP	
T217	NM_019750	Nat6	NP	
T186	NM_009400	Tnfrsf18	NP	
T123	NM_017470	Dnalc4	NP	
T1523	NM_178641	Inpp5f	NO CONCORDANCE	Choi JD, Underkoffler LA, Wood AJ, Collins JN, Williams PT, Golden JA, Schuster EF Jr, Loomes KM, Oakey RJ. "A novel variant of inpp5f is imprinted in brain, and its expression is correlated with differential methylation of an internal CpG island." Mol Cell Biol 2005 Jul,25(13):5514-22
T303	NM_021443	Ccl8	NP	
T1567	NM_172383	6330530A05Rik	NP	
T30118	NM_008965	Ptger4	NP	
T37660	NM_001033129	D6Ertd474e	NP	
T31276	NM_145499	Cyp2c70	NP	
T31371	NM_007982	Ptk2	NP	
T31391	NM_053115	Acox2	NP	
T31387	NM_016922	Gal3st1	NP	
T31390	NM_145597	Tmem161a	NP	
T31333	XM_001003747	2010321M09Rik	NP	
T31354	NM_010949	Numb	NP	
T31310	XM_001002673	0610007L01Rik	NP	
T38248	NM_134048	Cbll1	NP	
T39138	NM_146417	Olfr877	NP	
T38777	NM_001004162	LOC432436	NP	
T39388	AJ278965	Cd80	NP	
T40560	XM_905672	Apold1	NP	
T38815	NM_139306	Asah3l	NP	
T1777	NM_010827	Msc	CONCORDANCE	Robb L, Hartley L, Wang CC, Harvey RP, Begley CG. "musculin: a murine basic helix-loop-helix transcription factor gene expressed in embryonic skeletal muscle." Mech Dev 1998 Aug,76(1-2):197-201
T3345	NM_029285	1700001C02Rik	NP	
T6874	NM_011905	Tlr2	NP	
T35464	NM_008274	Hoxd12	CONCORDANCE	Albrecht AN, Schwabe GC, Stricker S, Boddrich A, Wanker EE, Mundlos S. "The synpolydactyly homolog (spdh) mutation in the mouse -- a defect in patterning and growth of limb cartilage elements." Mech Dev 2002 Mar,112(1-2):53-67
				Dolle P, Izpisua-Belmonte JC, Falkenstein H, Renucci A, Duboule D. "Coordinate expression of the murine Hox-5 complex homoeobox-containing genes during limb pattern formation." Nature 1989 Dec 14,342(6251):767-72
				Duboule D, "The function of Hox genes in the morphogenesis of the vertebrate limb." Ann Genet 1993,36(1):24-9
T580	NM_019950	Chst5	NP	
T36606	NM_010745	Ly86	NP	
T36623	NM_133197	Mcf2	NP	
T37492	NM_172904	Fsd2	NP	
T63298	NM_026866	Disp1	NO CONCORDANCE	Rice R, Connor E, Rice DP. "Expression patterns of Hedgehog signalling pathway members during mouse palate development." Gene Expr Patterns 2006 Jan,6(2):206-12
T70079	MIMAT0004940	mmu-miR-511	NP	
T70081	MIMAT0002889	mmu-miR-532-5p	NP	
T3537	NM_198703	Prkwnk1	NP	
T2205	NM_144828	Ppp1r1b	NP	
T1700	NM_177693	Lim2	NP	
T1555	NM_020625	Zfp297	NP	
T3639	NM_023243	Ccnh	NP	
T3619	NM_139307	Slitl2	NP	
T814	NM_013664	Sh3gl1	PARTIAL CONCORDANCE	So CW, Sham MH, Chew SL, Cheung N, So CK, Chung SK, Caldas C, Wiedemann LM, Chan LC. "Expression and protein-binding studies of the EEN gene family, new interacting partners for dynamin, synaptojanin and huntingtin proteins." Biochem J 2000 Jun 1,348 Pt 2():447-58
T858	NM_025388	Ufc1	NP	
T4520	NM_178758	C730027J19Rik	NP	
T2709	NM_019575	Scamp4	NP	
T4381	NM_133831	Gltscr2	NP	
T4425	NM_026601	Hyi	NP	
T5336	NM_147097	Olfr628	NP	
T6062	NM_138668	1810047C23Rik	NP	
T3420	NM_025448	Ssr2	NP	
T2050	NM_010098	Opn3	NP	

T5133	NM_172284	Ddx19b	NP	
T5180	NM_134058	Pelo	NP	
T5899	NM_053092	Kars	NP	
T2289	NM_025395	Chchd5	NP	
T4713	NM_025974	Rpl14	NP	
T4905	NM_172935	5730457F11Rik	NP	
T1774	NM_009666	Amelx	NP	
T6421	NM_009046	Relb	NO CONCORDANCE	Carrasco D, Ryseck RP, Bravo R. "Expression of relB transcripts during lymphoid organ development: specific expression in dendritic antigen-presenting cells." Development 1993 Aug,118(4):1221-31
T5752	NM_025903	Ifrd2	NP	
T5801	NM_011970	Psmb2	NP	
T7659	NM_025872	Golt1b	NP	
T9604	NM_015781	Nap1l1	NP	
T4964	NM_028769	Syvn1	NP	
T2918	NM_011354	Serf2	NP	
T23	NM_144787	Jmjd2c	NP	
T2872	NM_026612	Ndufb2	NP	
T37220	NM_174877	Zar1	NP	
T30261	NM_016715	Tpte2	NP	
T37477	NM_001024926	Cyb5d2	NP	
T37148	NM_027865	Tmem25	NP	
T30672	NM_026000	Psmd9	NP	
T37052	NM_011408	Slfn2	NP	
T38197	NM_030137	Cstad	NP	
T39901	XM_894331	EG629441	NP	
T39900	AK038984	LOC432449	NP	
T31673	NM_007985	Fancc	NP	
T39412	NM_010748	Lyst	NP	
T31828	NM_025749	Zfp474	NP	
T39450	BC100377	AU022751	NP	
T6124	NM_007386	Aco1	NO CONCORDANCE	Cankaya M, Hernandez AM, Ciftci M, Beydemir S, Ozdemir H, Budak H, Gulcin I, Comakli V, Emircupani T, Ekinci D, Kuzu M, Jiang Q, Eichele G, Kufrevioglu OI. "An analysis of expression patterns of genes encoding proteins with catalytic activities." BMC Genomics 2007,8():232
T32146	NM_172872	Ankrd38	NP	
T35071	XM_135805	Wdr44	NP	
T7055	NM_146236	Tceal1	NP	
T37506	NM_175511	A130092J06Rik	NP	
T37713	NM_027402	Fndc5	NP	
T5857	NM_134011	Tbrg4	NP	
T6305	NM_175356	Pik4cb	NP	
T6511	NM_021524	Pbef1	NP	
T6529	NM_010209	Fh1	PARTIAL CONCORDANCE ALSO IN OTHER TISSUES	Cankaya M, Hernandez AM, Ciftci M, Beydemir S, Ozdemir H, Budak H, Gulcin I, Comakli V, Emircupani T, Ekinci D, Kuzu M, Jiang Q, Eichele G, Kufrevioglu OI. "An analysis of expression patterns of genes encoding proteins with catalytic activities." BMC Genomics 2007,8():232
T6124	NM_007386	Aco1	NP	
T10017	NM_028115	Trub1	NP	
T36425	NM_010834	Gdf8	CONCORDANCE	McPherron AC, Lawler AM, Lee SJ. "Regulation of skeletal muscle mass in mice by a new TGF-beta superfamily member." Nature 1997 May 1,387(6628):83-90
T2452	NM_009605	Adipoq	NP	
T36831	NM_172585	Larp5	NP	
T38370	XM_137117	Adamts14	NP	
T40448	AK078202	6430502G17Rik	NP	
T40349	BC027793	Scfd1	NP	
T39628	XM_001004492	LOC677542	NP	
T45652	XM_001003389	LOC676704	NP	
T1554	NM_026186	1300013D05Rik	NP	
T2218	NM_031998	Tsga14	NP	
T947	NM_020036	Calm4	NP	
T417	NM_010118	Egr2	NO CONCORDANCE	Coulpier F, Le Crom S, Maro GS, Manent J, Giovannini M, Maciorowski Z, Fischer A, Gessler M, Charnay P, Topilko P. "Novel features of boundary cap cells revealed by the analysis of newly identified molecular markers." Glia 2009 Oct,57(13):1450-7
				Wilkinson DG, Bhatt S, Chavrier P, Bravo R, Charnay P. "Segment-specific expression of a zinc-finger gene in the developing nervous system of the mouse." Nature 1989 Feb 2,337(6206):4
T4450	NM_146063	BC031593	NP	
T2531	NM_007394	Acvr1	PARTIAL CONCORDANCE	Dudas M, Nagy A, Laping NJ, Moustakas A, Kaartinen V. "Tgf-beta3-induced palatal fusion is mediated by Alk-5/Smad pathway." Dev Biol 2004 Feb 1,266(1):96-108
				Minina E, Schneider S, Rosowski M, Lauster R, Vortkamp A. "Expression of Fgf and Tgfbeta signaling related genes during embryonic endochondral ossification." Gene Expr Patterns 2005 Dec,6(1):102-109
				Vesper AH, Raetzman LT, Camper SA. "Role of prophet of Pit1 (PROP1) in gonadotrope differentiation and puberty." Endocrinology 2006 Apr,147(4):1654-63
T3020	NM_025296	Wdr39	NP	
T3331	NM_028712	Rap2b	NP	
T3349	NM_009374	Tgm3	CONCORDANCE	Zhang J, Zhi HY, Ding F, Luo AP, Liu ZH. "Transglutaminase 3 expression in C57BL/6J mouse embryo epidermis and the correlation with its differentiation." Cell Res 2005 Feb,15(2):105-10
T1585	NM_029291	Ascc2	NP	
T6367	NM_007927	Emd	NP	
T6712	NM_146097	Cbwd1	NP	
T8259	NM_180588	2700029E10Rik	NP	
T5156	NM_172845	Adamts4	NP	
T7619	NM_029810	Nt5c2	NP	
T50010	NM_010456	Hoxa9	NP	
T50016	AB038697	Olig2	NO CONCORDANCE	Shibasaki K, Takebayashi H, Ikenaka K, Feng L, Gan L, "Expression of the basic helix-loop-factor Olig2 in the developing retina: Olig2 as a new marker for retinal progenitors and late-born cells." Gene Expr Patterns 2007 Jan,7(1-2):57-65
T373	NM_021450	Trpm7	NP	
T35716	XM_001000101	Tnks2	NP	
T37586	XM_893176	Znrf3	NP	
T37334	NM_001033397	Krt26	NP	
T36617	NM_016693	Map3k6	NP	
T36993	NM_022886	Scel	NP	
T30441	NM_178381	Trp53i5	CONCORDANCE	Rock JR, Harfe BD. "Expression of TMEM16 paralogs during murine embryogenesis." Dev Dyn 2008 Sep,237(9):2566-74
T38055	NM_205820	Tlr13	NP	
T31298	NM_025911	Ccdc91	NP	
T31349	NM_029012	Sppl3	NP	
T31544	NM_025408	Phca	NP	
T38469	NM_001003911	Adamts7	NP	
T38458	XM_980092	Krt28	NP	
T38473	NM_213728	Krt72	NP	
T39062	NM_011310	S100a3	NP	
T861	NM_013640	Psmb10	NP	
T99	NM_025626	3110001A13Rik	NP	
T4485	NM_026362	5033414D02Rik	NP	
T4494	NM_013796	Nagpa	NP	

T4542	NM_145139	Eif3s6ip	NP	
T5657	NM_178698	Pigv	NP	
T3123	NM_028126	2610019A05Rik	NP	
T2708	NM_027185	Def6	NP	
T2726	NM_023260	Mrps34	NP	
T4411	NM_010545	Ii	NP	
T2550	NM_011530	Tap2	NP	
T4559	NM_009387	Tk1	NO CONCORDANCE	Sansom SN, Griffiths DS, Faedo A, Kleinjan DJ, Ruan Y, Smith J, van Heyningen V, Rubenstein JL, Livesey FJ. "The level of the transcription factor Pax6 is essential for controlling the balance between neural stem cell self-renewal and neurogenesis." PLoS Genet 2009 Jun,5(6):e1000511
T1147	NM_009812	Casp8	NP	
T4593	NM_146011	Arhgap9	NP	
T4621	NM_023372	Rpl38	NP	
T5480	NM_025624	2510048O06Rik	NO CONCORDANCE	Sansom SN, Griffiths DS, Faedo A, Kleinjan DJ, Ruan Y, Smith J, van Heyningen V, Rubenstein JL, Livesey FJ. "The level of the transcription factor Pax6 is essential for controlling the balance between neural stem cell self-renewal and neurogenesis." PLoS Genet 2009 Jun,5(6):e1000511
T4631	NM_027139	Taf9	NO CONCORDANCE	Sansom SN, Griffiths DS, Faedo A, Kleinjan DJ, Ruan Y, Smith J, van Heyningen V, Rubenstein JL, Livesey FJ. "The level of the transcription factor Pax6 is essential for controlling the balance between neural stem cell self-renewal and neurogenesis." PLoS Genet 2009 Jun,5(6):e1000511
T5489	NM_025907	1600013P15Rik	NP	
T5499	NM_025582	2810405K02Rik	NP	
T5515	NM_030711	Arts1	NP	
T5538	NM_198031	Tubgcp3	NP	
T3407	NM_010220	Fkbp5	NP	
T1867	NM_010370	Gzma	NP	
T5124	NM_146205	Armc5	NP	
T5126	NM_153795	BC032204	NP	
T2086	NM_172468	4732481H14Rik	NP	
T5288	NM_053180	Ccrk	NP	
T5298	NM_009761	Bnip3l	NO CONCORDANCE	Sansom SN, Griffiths DS, Faedo A, Kleinjan DJ, Ruan Y, Smith J, van Heyningen V, Rubenstein JL, Livesey FJ. "The level of the transcription factor Pax6 is essential for controlling the balance between neural stem cell self-renewal and neurogenesis." PLoS Genet 2009 Jun,5(6):e1000511
T2148	NM_153788	Centb1	NP	
T4840	NM_199016	Enpp4	NP	
T4679	NM_026829	Mthfs	NP	
T5383	NM_016684	Zfp96	NP	
T5916	NM_134154	AW491445	NP	
T6732	NM_010378	H2-Aa	NP	
T6710	NM_026070	2900091E11Rik	NP	
T6701	NM_010696	Lcp2	NP	
T5700	NM_007497	Atf1	NP	
T7039	NM_175397	5830484A20Rik	NP	
T7306	NM_013566	Itgb7	NP	
T6175	NM_024253	Nkg7	NP	
T6132	NM_009151	Selpl	NP	
T6119	NM_008279	Map4k1	NP	
T6395	NM_022024	Gmfg	NP	
T6429	NM_009277	Trim21	NP	
T5756	NM_010379	H2-Ab1	NP	
T6500	NM_021327	Tnip1	NP	
T8062	NM_172435	P2ry10	NP	
T8264	NM_013487	Cd3d	NP	
T9559	NM_033606	Dgx1	NP	
T9583	NM_011053	Pdcd11	NP	
T6541	NM_010386	H2-DMa	NP	
T6570	NM_008225	Hcls1	NP	
T6555	NM_010877	Ncf2	NP	
T533	NM_026192	Calcoco1	NP	
T6567	NM_145559	Slc2a9	NP	
T35102	NM_175105	Aqp11	NP	
T35018	NM_178650	Tbc1d10c	NP	
T35758	NM_030732	Tbl1xr1	NP	
T6711	NM_008152	Gpr65	NP	
T7349	NM_010693	Lck	NP	
T9917	NM_207246	Rasgrp3	NP	
T7093	NM_008368	Il2rb	NP	
T9918	NM_001004184	MGC74379	NP	
T3025	NM_011190	Psme2	NP	
T2028	NM_183264	5830405N20Rik	NP	
T35133	NM_007720	Ccr8	NP	
T35190	NM_019925	Gpr132	NP	
T35655	NM_028657	F630110N24Rik	NP	
T36240	NM_173028	Vps13a	NP	
T2801	NM_007651	Cd53	NP	
T2820	NM_134116	Gpsm3	NP	
T2888	NM_023137	Ubd	NP	
T2937	NM_016933	Ptprcap	NP	
T36133	NM_007648	Cd3e	CONCORDANCE	Hetzer-Egger C, Schorpp M, Haas-Assenbaum A, Balling R, Peters H, Boehm T. "Thymopoiesis requires Pax9 function in thymic epithelial cells." Eur J Immunol 2002 Apr,32(4):1175-81
T1223	NM_028058	Fundc1	NP	
T1211	NM_009533	Xrcc5	NP	
T3455	NM_018729	Cd244	NP	
T2454	NM_013542	Gzmb	NP	
T2424	NM_172900	Siglec10	NP	
T35818	XM_905818	A430107D22Rik	NP	
T37003	NM_017461	Sept1	NP	
T36899	NM_011159	Prkdc	NP	
T36907	NM_013585	Psmb9	NP	
T36908	NM_025959	Psmc6	NP	
T36215	NM_009985	Ctsw	NP	
T36527	NM_008400	Itgal	NP	
T36949	NM_018750	Rassf5	NP	
T31100	NM_021274	Cxcl10	PARTIAL CONCORDANCE	Visel A, Thaller C, Eichele G. "GenePaint.org: an atlas of gene expression patterns in the mouse embryo." Nucleic Acids Res 2004 Jan 1,32(Database issue):D552-6
T31131	NM_133978	Cmtm7	NP	
T31140	NM_011854	Oasl2	NP	
T30531	NM_027222	2010001M09Rik	NP	
T30282	NM_019583	Il17rb	NP	
T30799	NM_145545	Gbp6	NP	
T31172	NM_007763	Crip1	NP	
T31200	NM_009895	Cish	NP	
T31211	NM_030253	Parp9	CONCORDANCE	Hakme A, Huber A, Dolle P, Schreiber V. "The macroPARP genes Parp-9 and Parp-14 are developmentally and differentially regulated in mouse tissues." Dev Dyn 2008 Jan,237(1):209-15
T38318	NM_025846	Rras2	NP	
T38314	NM_025396	Pgls	NP	
T31243	NM_007783	Csk	NP	
T36293	XM_908064	Dnajc13	NP	

T36570	NM_013707	Krtap14	NP	
T36604	NM_013825	Ly75	NP	
T9961	NM_008527	Klrb1c	NP	
T37770	NM_183390	Klhl6	NP	
T30348	XM_486159	2310066E14Rik	NP	
T36501	NM_008359	Il17ra	NP	
T36123	NM_009138	Ccl25	NP	
T37006	NM_008458	Serpina3c	NP	
T37053	NM_011409	Slfn3	PARTIAL CONCORDANCE	Visel A, Thaller C, Eichele G. "GenePaint.org: an atlas of gene expression patterns in the mouse embryo." Nucleic Acids Res 2004 Jan 1,32(Database issue):D552-6
T31279	NM_008365	Il18r1	NP	
T31285	NM_019640	Pitpnb	NP	
T38070	NM_021053	Tscot	NP	
T31336	NM_026218	Fgfr1op2	NP	
T31435	NM_023514	Mrps9	NP	
T31590	XM_916862	Nalp6	NP	
T31931	NM_001038664	Gngt2	NP	
T38672	NM_028785	Dock8	NP	
T39052	NM_011815	Fyb	NP	
T39623	XM_622820	LOC547338	NP	
T8284	NM_010161	Evi2a	NP	
T45280	NM_175479	A330008L17Rik	NP	
T45003	AK030875	AI427122	NP	
T31637	NM_010724	Psmb8	NP	
T38286	NM_007645	Cd37	NP	
T31443	NM_016857	Exoc7	NP	
T63013	NM_018872	D1Bwg0491e	NP	
T491	NM_178911	Pld4	NP	
T30275	NM_013591	Madcam1	NP	
T30080	NM_009875	Cdkn1b	NP	
T4745	NM_177450	Cndp1	NP	
T4117	NM_207680	Bcl2l11	NP	
T1002	NM_026183	1300013J15Rik	NP	
T995	NM_008269	Hoxb6	PARTIAL CONCORDANCE	Graham A, Maden M, Krumlauf R. The murine Hox-2 genes display dynamic dorsoventral patterns of expression during central nervous system development. Development 1991 May,112(1):255-64 Concordance with picture from Hoffman BG, Zavaglia B, Witzsche J, Ruiz de Algara T, Beach M, Hoodless PA, Jones SJ, Marra MA, Helgason CD. "Identification of transcripts with enriched expression in the developing and adult pancreas." Genome Biol 2008,9(6):R99
T299	NM_146028	Stac2	NP	
T6362	AK166607	Steap2	NP	
T7563	NM_133894	Ugt2b38	NP	
T8699	NM_013910	Fbxl10	NP	
T7626	NM_009327	Tcf1	PARTIAL CONCORDANCE	Suh JM, Yu CT, Tang K, Tanaka T, Kodama T, Tsai MJ, Tsai SY, "The expression profiles of nuclear receptors in the developing and adult kidney." Mol Endocrinol 2006 Dec,20(12):3412-20
T5441	NM_153535	BC035537	NP	
T455	NM_015749	Tcn2	NP	
T9926	NM_026085	3110049J23Rik	NP	
T1358	NM_021517	Pdzk1	NP	
T35983	NM_173427	Klhdc7a	NP	
T7191	NM_146071	Muc20	NP	
T36846	NM_008982	Ptprj	NP	
T30840	NM_008116	Ggt1	NP	
T30857	NM_007607	Car4	CONCORDANCE	Cankaya M, Hernandez AM, Ciftci M, Beydemir S, Ozdemir H, Budak H, Gulcin I, Comakli V, Emircupani T, Ekinci D, Kuzu M, Jiang Q, Eichele G, Kufrevioglu OI. "An analysis of expression patterns of genes encoding proteins with catalytic activities." BMC Genomics 2007,8():232
T37514	NM_001004150	A4galt	NP	
T30962	NM_027857	Acy3	NP	
T31050	NM_016785	Tpmt	NP	
T37659	XM_284236	D630042F21Rik	NP	
T31548	NM_178413	BC051244	NP	
T31646	NM_133962	Arhgef18	NP	
T32005	NM_030021	D730039F16Rik	NP	
T38208	NM_010018	Dao1	NP	
T39640	XM_001003628	1110060D06Rik	NP	
T51042	BC022226	Slc5a2	NP	
T51027	NM_183354	Slc12a1	NP	
T3986	NM_018802	Syt8	NP	
T5860	NM_027320	Ifi35	NP	
T5995	NM_009357	Tex261	NP	
T6691	NM_029763	Polr3f	NP	
T6628	NM_027838	Senp8	NP	
T6854	NM_170591	Nupl1	NP	
T7684	NM_025274	Dppa5	NP	
T4919	NM_018869	Gprk5	NP	
T2609	NM_011901	Taf7	NP	
T484	NM_008508	Lor	NP	
T8093	NM_009337	Tcl1	NP	
T36303	NM_139218	Dppa3	NP	
T233	NM_145546	Gtf2b	NP	
T3460	NM_011328	Sct	NO CONCORDANCE	Siu FK, Sham MH, Chow BK. "Secretin, a known gastrointestinal peptide, is widely expressed during mouse embryonic development." Gene Expr Patterns 2005
T2492	NM_030564	Rnf34	NP	
T37312	NM_028034	2410004F06Rik	NP	
T31260	NM_146198	Slc5a11	NP	
T37647	XM_125673	Cxxc6	NP	
T31584	NM_001010826	Kctd14	NP	
T39844	BC059932	2410025L10Rik	NP	
T63197	NM_145833	Lin28	NP	
T39525	AK145040	Gtf3c3	NP	
T39854	NM_001042503	Trim71	NP	
T70394	MIMAT0000538	mmu-miR-31	NP:microRNA	
T35928	NM_007445	Amh	CONCORDANCE	Jamin SP, Arango NA, Mishina Y, Hanks MC, Behringer RR. "Requirement of Bmpr1a for Mullerian duct regression during male sexual development." Nat Genet 2002 Nov,32(3):408-10
T9706	NM_026489	Hormad1	NP	
T36277	NM_010029	Ddx4	NP	
T40010	XR_002340	6330411D24Rik	NP	
T4760	NM_024412	Clcnka	NP	
T36726	NM_139310	Otoa	NP	
T37121	NM_009347	Tecta	CONCORDANCE	Rau A, Legan PK, Richardson GP. "Tectorin mRNA expression is spatially and temporally restricted during mouse inner ear development." J Comp Neurol 1999 Mar 8,405(2):271-80
T37122	NM_009350	Tenr	NP	
T37639	XM_908254	Cldn22	NP	

T30400	NM_010953	Oc90	CONCORDANCE	Li S, Mark S, Radde-Gallwitz K, Schlisner R, Chin MT, Chen P. "Hey2 functions in parallel with Hes1 and Hes5 for mammalian auditory sensory organ development." BMC Dev Biol 2008,8():20
T38650	NM_001018019	AY616753	NP	
T40039	XM_992778	EG624918	NP	
T45144	NM_013624	Otog	NP	
T31019	NM_027172	2310046K01Rik	NP	
T37802	NM_001009574	Taar5	NP	
T37815	NM_001033292	Espnl	NP	
T1692	NM_153158	E130308A19Rik	NP	
T9121	NM_007774	Cryga	CONCORDANCE	Santhiya ST, Abd-alla SM, Loster J, Graw J. "Reduced levels of gamma-crystallin transcripts during embryonic development of murine Cat2nop mutant lenses." Graefes Arch Clin Exp Ophthalmol 1995 Dec,233(12):795-800
T1845	NM_009965	Cryba1	NP	
T35173	NM_016975	Gja3	NP	
T35175	NM_008123	Gja8	NP	
T30651	NM_009751	Bfsp1	NP	
T37225	NM_178679	Zfp365	NP	
T39036	NM_011939	Hsf4	NP	
T36200	NM_007773	Crybb2	NP	
T36517	XM_909063	Ipo8	NP	
T37688	XR_005138	E130119H09Rik	NP	
T36110	NM_007601	Capn3	CONCORDANCE	Herasse M, Ono Y, Fougerousse F, Kimura E, Stockholm D, Beley C, Montarras D, Pinset C, Sorimachi H, Suzuki K, Beckmann JS, Richard I. "Expression and functional characteristics of calpain 3 isoforms generated through tissue-specific transcriptional and posttranscriptional events." Mol Cell Biol 1999 Jun,19(6):4047-55
T37644	NM_153076	Crygn	NP	
T30396	NM_019689	Arid3b	NP	
T32146	NM_172872	Ankrd38	NP	
T40285	NM_028736	Grip1	NP	
T39505	AK043703	A830021M18	NP	
T39438	XM_112126	Nhs	PARTIAL CONCORDANCE	Burdon KP, McKay JD, Sale MM, Russell-Eggitt IM, Mackey DA, Wirth MG, Elder JE, Nicoll A, Clarke MP, FitzGerald LM, Stankovich JM, Shaw MA, Sharma S, Gajovic S, Gruss P, Ross S, Thomas P, Voss AK, Thomas T, Gecz J, Craig JE. "Mutations in a novel gene, NHS, cause the pleiotropic effects of Nance-Horan syndrome, including severe congenital cataract, dental anomalies, and mental retardation." Am J Hum Genet 2003 Nov,73(5):1120-30
T45578	NM_028029	Dnmbp	NP	
T818	NM_176963	Galm	NP	
T410	NM_134017	Mat2b	NP	
T910	NM_007954	Es1	NP	
T4777	NM_009997	Cyp2a4	NP	
T4445	NM_008971	Ptk9	NP	
T4562	NM_201234	D630030L16Rik	NP	
T5330	NM_147091	Olfr568	NP	
T5328	NM_146822	Olfr640	NP	
T5333	NM_147072	Olfr641	NP	
T5353	XM_358344	Cep2	NP	
T5260	NM_146177	Suv420h2	NP	
T2137	NM_024244	1200015N20Rik	NP	
T163	NM_133699	Atp6v1c2	NP	
T5331	NM_001011536	Olfr566	NP	
T5322	NM_147088	Olfr569	NP	
T5314	NM_147085	Olfr571	NP	
T5317	NM_147115	Olfr578	NP	
T5319	NM_147052	Olfr589	NP	
T5321	NM_013621	Olfr69	NP	
T1094	NM_008867	Pla2g1br	NP	
T4312	NM_203509	NP_TR6JSE50FPA	NP	
T4182	NM_144917	Rbed1	NP	
T4357	NM_198311	Ttc8	NP	
T4186	NM_173862	BC030396	NP	
T6320	NM_030074	Zfp687	NP	
T7397	NM_183161	BC019537	NP	
T5943	NM_020276	Nelf	PARTIAL CONCORDANCE	Kramer PR, Wray S. "Nasal embryonic LHRH factor (NELF) expression within the CNS and PNS of the rodent." Brain Res Gene Expr Patterns 2001 Aug,1(1):23-6
T6318	NM_027548	Serpinb7	NP	
T7771	NM_178414	BC048390	NP	
T6385	NM_198623	Ubqln3	NP	
T6328	NM_008299	Dnajb3	NP	
T6981	NM_172338	Dnajc16	NP	
T7794	NM_080467	Atp6v0a4	NP	
T7787	NM_011126	Plunc	CONCORDANCE	LeClair EE, Nguyen L, Bingle L, MacGowan A, Singleton V, Ward SJ, Bingle CD. "Genomic organization of the mouse plunc gene and expression in the developing airways and thymus." Biochem Biophys Res Commun 2001 Jun 15,284(3):792-7
T7824	NM_028934	4930452B06Rik	NP	
T7821	NM_175402	Rbm15b	NP	
T7855	NM_001008230	Gm605	NP	
T7204	NM_011183	Psen2	NP	
T6415	NM_001004066	Zfp386	NP	
T7539	NM_177406	MGC25972	NP	
T7540	NM_021372	Sertad2	NP	
T7694	NM_013809	Cyp2g1	NP	
T7704	NM_008578	Mef2b	NO CONCORDANCE	Molkentin JD, Firulli AB, Black BL, Martin JF, Hustad CM, Copeland N, Jenkins N, Lyons G, Olson EN. "MEF2B is a potent transactivator expressed in early myogenic lineages." Mol Cell Biol 1996 Jul,16(7):3814-24
T7712	NM_018751	Sult1c1	NP	
T8055	NM_201370	BC052883	NP	
T7591	NM_007647	Entpd5	NP	
T8722	NM_027977	2310001A20Rik	NP	
T8770	NM_182995	6330503K22Rik	NP	
T4143	NM_144536	Cdkal1	NP	
T348	NM_145355	Rnf185	NP	
T7494	NM_144820	1700009P13Rik	NP	
T8482	NM_029992	A930031F18Rik	NP	
T8814	NM_009370	Tgfbr1	NO CONCORDANCE	Minina E, Schneider S, Rosowski M, Lauster R, Vortkamp A. Expression of Fgf and Tgfbeta signaling related genes during embryonic endochondral ossification., Gene Expr Patterns 2005 Dec,6(1):102-109
T35025	XM_888129	2300003P22Rik	NP	
T35057	XM_485677	2410131K14Rik	NP	
T35016	NM_027238	1810054D07Rik	NP	
T35079	NM_028258	Dzip1l	NP	
T35768	XM_001005685	9130229H14Rik	NP	
T35785	NM_001024619	Lrrc54	NP	
T8171	NM_023247	4733401H18Rik	NP	
T8176	NM_009477	Upp1	NP	
T7916	NM_028226	3000004N20Rik	NP	
T9480	NM_028298	Zfp655	NO CONCORDANCE	Visel A, Thaller C, Eichele G. "GenePaint.org: an atlas of gene expression patterns in the mouse embryo." Nucleic Acids Res 2004 Jan 1,32(Database issue):D552-6

T3960	NM_145973	Ell3	NP	
T35322	NM_134216	V1rh7	NP	
T35935	NM_023617	Aox3	NP	
T3062	NM_011260	Reg3g	NP	
T35981	NM_175516	Lrrn6c	NP	
T77	NM_172891	Styk1	NP	
T387	NM_144890	BC018465	NP	
T7114	NM_016807	Sdcbp	NP	
T7206	NM_017465	Sult2b1	NP	
T35644	NM_025725	Ccdc96	NP	
T35650	XM_910825	4921528H16Rik	NP	
T36192	NM_007755	Cpeb1	NP	
T35476	NM_181853	Trim66	NP	
T35444	NM_019739	Foxo1	PARTIAL CONCORDANCE	Hoekman MF, Jacobs FM, Smidt MP, Burbach JP. "Spatial and temporal expression of FoxO transcription factors in the developing and adult murine brain. " Gene Expr Patterns 2006 Jan,6(2):134-40
T3496	NM_023631	Aox4	NP	
T36638	NM_026779	Mocos	NP	
T37020	NM_145838	St8sia6	NP	
T30873	NM_007837	Ddit3	NP	
T30926	NM_145532	Mall	NP	
T31110	NM_023135	Sult1e1	NP	
T31771	NM_134109	Ildr1	NP	
T30516	NM_172205	Sbsn	NP	
T30226	NM_145554	Ldlrap1	NP	
T30794	NM_028634	Pgea1	NP	
T31226	NM_009736	Bag1	NP	
T38110	NM_031391	Gtf2a1	NP	
T31239	BC031933	Lipl3	NP	
T38356	XM_924923	Fmo6	NP	
T9396	NM_026298	4930553F24Rik	NP	
T9430	NM_008448	Kif5b	NP	
T36587	NM_144556	Lgi4	NP	
T37399	XM_133663	4931431F19Rik	NP	
T37336	XM_143418	Ankrd35	NP	
T37388	XM_127142	4930573I19Rik	NP	
T37531	NM_177389	Mia3	NP	
T37532	NM_178778	A930041I02Rik	NP	
T30379	BC049156	Ift74	NP	
T30323	NM_009379	Thpo	NP	
T31714	NM_001001182	Baz2b	NP	
T4455	NM_198023	Rcor1	PARTIAL CONCORDANCE	Jensen P, Magdaleno S, Lehman KM, Rice DS, Lavallie ER, Collins-Racie L, McCoy JM, Curran T. "A neurogenomics approach to gene expression analysis in the developing brain." Brain Res Mol Brain Res 2004 Dec 20,132(2):116-27
T30681	NM_178775	Rps6kc1	NP	
T37038	XM_925590	Slc4a11	NP	
T35795	NM_172836	9930021J03Rik	NP	
T30419	XM_908714	Cnfn	NP	
T30412	NM_053184	Ugt2a1	NP	
T30420	NM_001039042	Klk13	NP	
T30395	XM_978169	Cep63	NP	
T31250	NM_175250	2810007J24Rik	NP	
T37668	XM_885173	D930020B18Rik	NP	
T31355	NM_053262	Dhrs8	NP	
T38250	NM_010092	Dyrk1b	NP	
T39096	NM_146867	Olfr131	NP	
T39098	NM_146852	Olfr1339	NP	
T39100	NM_146541	Olfr1361	NP	
T39103	NM_146467	Olfr1388	NP	
T39105	NM_146877	Olfr1395	NP	
T39106	NM_020515	Olfr140	CONCORDANCE	Kolterud A, Alenius M, Carlsson L, Bohm S. "The Lim homeobox gene Lhx2 is required for olfactory sensory neuron identity." Development 2004 Nov,131(21):5319-26
T39108	NM_146881	Olfr1404	NP	
T39109	NM_146491	Olfr1410	NP	
T39110	NM_146410	Olfr1420	NP	
T39111	NM_146806	Olfr143	NP	
T39114	NM_146335	Olfr19	NP	
T39115	NM_001001807	Olfr234	NP	
T39116	NM_146606	Olfr24	NP	
T39117	NM_146457	Olfr282	NP	
T39119	NM_146281	Olfr284	NP	
T39121	NM_146538	Olfr315	NP	
T39233	NM_146374	Olfr368	NP	
T39128	NM_146722	Olfr429	NP	
T39243	NM_001011742	Olfr479	NP	
T39244	NM_146952	Olfr522	NP	
T39260	NM_147100	Olfr614	NP	
T39261	NM_147080	Olfr615	NP	
T39266	NM_013616	Olfr64	NP	
T39268	NM_147074	Olfr653	NP	
T39271	NM_013619	Olfr67	NP	
T39272	NM_146760	Olfr672	NP	
T39275	NM_207557	Olfr681	NP	
T39277	NM_001011857	Olfr685	NP	
T39280	NM_147061	Olfr691	NP	
T39281	NM_019486	Olfr71	NP	
T39283	NM_146392	Olfr720	NP	
T39288	NM_146664	Olfr734	NP	
T39290	NM_146666	Olfr736	NP	
T39291	NM_146299	Olfr745	NP	
T39298	NM_001011748	Olfr867	NP	
T38655	XM_485838	Klhdc5	NP	
T39259	NM_147081	Olfr610	NP	
T39300	NM_146816	Olfr923	NP	
T39302	NM_146514	Olfr96	NP	
T39548	XM_980715	EG231836	NP	
T39251	NM_146361	Olfr557	NP	
T39050	XM_001001987	Brd1	NP	
T39556	XM_985665	LOC671232	NP	
T45078	NM_010272	Gdf11	PARTIAL CONCORDANCE	Nakashima M, Toyono T, Akamine A, Joyner A. " Expression of growth/differentiation factor 11, a new member of the BMP/TGFbeta superfamily during mouse embryogenesis." Mech Dev 1999 Feb,80(2):185-9
T30382	XM_128924	Rbm27	NP	
T39160	NM_147013	Olfr1038	NP	
T39161	NM_146577	Olfr1043	NP	
T39162	NM_147010	Olfr1052	NP	
T39164	NM_001011825	Olfr1105	NP	
T39133	NM_146821	Olfr629	NP	
T39134	NM_146814	Olfr665	NP	
T39136	NM_147032	Olfr705	NP	
T39137	NM_146682	Olfr76	NP	
T39139	NM_146330	Olfr958	NP	

T39141	NM_146854	Olfr982	NP	
T39142	NM_146855	Olfr985	NP	
T38787	NM_009701	Aqp5	NP	
T39163	NM_146767	Olfr1104	NP	
T39165	NM_146752	Olfr1106	NP	
T39168	NM_001011868	Olfr1178	NP	
T39169	NM_146917	Olfr1179	NP	
T39171	NM_146630	Olfr123	NP	
T39172	NM_146789	Olfr1230	NP	
T39173	NM_146454	Olfr1231	NP	
T39174	NM_147062	Olfr124	NP	
T39175	NM_146290	Olfr125	NP	
T39176	NM_146794	Olfr1263	NP	
T39177	NM_146793	Olfr1271	NP	
T39178	NM_146396	Olfr1277	NP	
T39181	NM_207240	Olfr1320	NP	
T39182	NM_207631	Olfr1321	NP	
T39184	NM_146390	Olfr1323	NP	
T39166	NM_146661	Olfr1112	NP	
T39365	AK047713	C030016D13Rik	NP	
T39179	NM_146400	Olfr1288	NP	
T39186	NM_146398	Olfr1325	NP	
T39187	NM_177061	Olfr1344	NP	
T39188	NM_207136	Olfr1349	NP	
T39189	NM_146389	Olfr1350	NP	
T39190	NM_001011737	Olfr1357	NP	
T39192	NM_146533	Olfr1367	NP	
T39199	NM_146683	Olfr1441	NP	
T39201	NM_146505	Olfr148	NP	
T39202	NM_001011832	Olfr1490	NP	
T39203	NM_146989	Olfr1496	NP	
T39204	NM_008762	Olfr15	NP	
T39206	NM_020514	Olfr1509	NP	
T39209	NM_010983	Olfr2	NP	
T39210	NM_146912	Olfr211	NP	
T39211	NM_146759	Olfr214	NP	
T39212	NM_001011789	Olfr222	NP	
T39213	NM_146429	Olfr223	NP	
T39649	NM_178214	Hist2h2be	NP	
T40283	AK014457	Traf3ip1	NP	
T30408	NM_027041	1700003M02Rik	NP	
T31480	NM_146107	Actr1b	NP	
T37968	XM_203999	Rapgef2	NP	
T63095	XM_358344	Cep2	NP	
T63370	NM_028932	Eaf1	NP	
T39758	AK078387	6530439I21	NP	
T45417	XM_917798	Cecr2	NP	
T7707	NM_146520	Olfr536	NP	
T7722	NM_146339	Olfr77	NP	
T38844	NM_183147	Sprn	NP	
T70019	MIMAT0001537	mmu-miR-429	NP	
T70305	MIMAT0000221	mmu-miR-191	NP	
T70269	MIMAT0000153	mmu-miR-141	NP	
T70270	MIMAT0000155	mmu-miR-142-3p	NP	
T70319	MIMAT0000519	mmu-miR-200a	CONCORDANCE	Choi PS, Zakhary L, Choi WY, Caron S, Alvarez-Saavedra E, Miska EA, McManus M, Harfe B, Giraldez AJ, Horvitz RH, Schier AF, Dulac C. "Members of the miRNA-200 family regulate olfactory neurogenesis." Neuron 2008 Jan 10,57(1):41-55
T70321	MIMAT0000657	mmu-miR-200c	NP	
T70356	MIMAT0000534	mmu-miR-26b	NP	
T70358	MIMAT0000126	mmu-miR-27b	NP	
T1897	NM_008245	Hhex	NP	
T4827	NM_027391	0610009A07Rik	NP	
T37133	NM_009375	Tg	NP	
T45492	NM_019804	B4galt4	NP	
T328	NM_029688	Srxn1	NP	
T847	NM_080289	Grhpr	NP	
T4605	NM_177322	Agtr1	NP	
T5203	NM_146154	Ppp1r8	NP	
T2996	NM_025384	Dnajd1	NP	
T2213	NM_027828	9030611O19Rik	NP	
T1096	NM_025826	Acadsb	NP	
T7620	NM_007809	Cyp17a1	CONCORDANCE	Keeney DS. "Perspectives in steroid hydroxylase gene expression: novel sites of expression during embryonic development." Endocr Res 1995 Feb-May,21(1-2):103-7
T8009	NM_013821	Hsd3b6	NP	
T8283	NM_153779	Amid	NP	
T7281	NM_026566	9430023L20Rik	NP	
T35021	NM_025929	2010109I03Rik	NP	
T8218	NM_026343	Stx17	NP	
T6795	NM_013463	Gla	NP	
T6708	NM_172398	2310005E10Rik	NP	
T7219	NM_146099	D19Wsu162e	NP	
T35261	NM_008560	Mc2r	NP	
T36524	NM_008396	Itga2	NO CONCORDANCE	
T37215	NM_011723	Xdh	NP	
T36514	NM_010568	Insr	NP	
T36592	NM_022883	Lpin3	NP	
T9948	NM_178098	4930486L24Rik	NP	
T37710	NM_177653	F830045P16Rik	NP	
T31041	NM_133688	Lyrm5	NP	
T35545	NM_032005	Tbx19	CONCORDANCE	Liu J, Lin C, Gleiberman A, Ohgi KA, Herman T, Huang HP, Tsai MJ, Rosenfeld MG. "Tbx19, a tissue-selective regulator of POMC gene expression." Proc Natl Acad Sci U S A 2001 Jul 17,98(15):8674-9
T8212	NM_020282	Nqo2	NP	
T36053	NM_138313	Bmf	NP	
T38673	NM_027533	Tspan2	NP	
T70187	MIMAT0000677	mmu-miR-7a	NP	
T3842	NM_007424	Agc1	CONCORDANCE	Gori F, Schipani E, Demay MB. "Fibromodulin is expressed by both chondrocytes and osteoblasts during fetal bone development." J Cell Biochem 2001 Apr 2-27,82(1):46-57
T50123	NM_007492	Arx	CONCORDANCE	Kawaguchi A, Ikawa T, Kasukawa T, Ueda HR, Kurimoto K, Saitou M, Matsuzaki F. "Single-cell gene profiling defines differential progenitor subclasses in mammalian neurogenesis." Development 2008 Sep,135(18):3113-24
T30231	NM_007554	Bmp4	PARTIAL CONCORDANCE	Minina E, Wenzel HM, Kreschel C, Karp S, Gaffield W, McMahon AP, Vortkamp A, BMP and Ihh/PTHrP signaling interact to coordinate chondrocyte proliferation and differentiation., Development 2001 Nov,128(22):4523-34
T1147	NM_009812	Casp8	NP	
T8264	NM_013487	Cd3d	NP	

T36133	NM_007648	Cd3e	CONCORDANCE	Dear TN, Boehm T. "Diverse mRNA expression patterns of the mouse calpain genes Capn5, Capn6 and Capn11 during development." Mech Dev 1999 Dec,89(1-2):201-9
T31237	XM_985866	Chd7	CONCORDANCE	Hurd EA, Capers PL, Blauwkamp MN, Adams ME, Raphael Y, Poucher HK, Martin DM, Loss of Chd7 function in gene-trapped reporter mice is embryonic lethal and associated with severe defects in multiple developing tissues., Mamm Genome 2007 Feb,18(2):94-104
T3863	NM_009604	Chrng	CONCORDANCE	
T4128	NM_007701	Chx10	CONCORDANCE	Rowan S, Chen CM, Young TL, Fisher DE, Cepko CL. "Transdifferentiation of the retina into pigmented cells in ocular retardation mice defines a new function of the homeodomain gene Chx10." Development 2004 Oct,131(20):5139-52
T30308	NM_025771	Cntnap2	NP	
T45058	NM_009925	Col10a1	CONCORDANCE	Long F, Zhang XM, Karp S, Yang Y, McMahon A.," Genetic manipulation of hedgehog signaling in the endochondral skeleton reveals a direct role in the regulation of chondrocyte proliferation." Development 2001 Dec,128(24):5099-108
T7865	NM_007729	Col11a1	CONCORDANCE	Yoshioka H, Iyama K, Inoguchi K, Khaleduzzaman M, Ninomiya Y, Ramirez F. "Developmental pattern of expression of the mouse alpha 1 (XI) collagen gene (Col11a1)." Dev Dyn 1995 Sep,204(1):41-7
T7815	NM_009929	Col18a1	PARTIAL CONCORDANCE	Schmidt A, Wenzel D, Ferring I, Kazemi S, Sasaki T, Hescheler J, Timpl R, Addicks K, Fleischmann BK, Bloch W. "Influence of endostatin on embryonic vasculo- and angiogenesis." Dev Dyn 2004 Jul,230(3):468-80
T38288	NM_031163	Col2a1	CONCORDANCE	Cheah KS, Lau ET, Au PK, Tam PP. "Expression of the mouse alpha 1(II) collagen gene is not restricted to cartilage during development." Development 1991 Apr,111(4):945-53
T38288	NM_031163	Col2a1	CONCORDANCE	Cheah KS, Lau ET, Au PK, Tam PP. "Expression of the mouse alpha 1(II) collagen gene is not restricted to cartilage during development." Development 1991 Apr,111(4):945-53
T9357	NM_009931	Col4a1	NO CONCORDANCE	Wu JE, Santoro SA. "Differential expression of integrin alpha subunits supports distinct roles during lung branching morphogenesis." Dev Dyn 1996 Jun,206(2):169-81
T63117	NM_007736	Col4a5	PARTIAL CONCORDANCE	Jensen P, Magdaleno S, Lehman KM, Rice DS, Lavallie ER, Collins-Racie L, McCoy JM, Curran T. "A neurogenomics approach to gene expression analysis in the developing brain." Brain Res Mol Brain Res 2004 Dec 20,132(2):116-27
T30372	NM_007740	Col9a1	CONCORDANCE	Perala M, Savontaus M, Metsaranta M, Vuorio E. "Developmental regulation of mRNA species for types II, IX and XI collagens during mouse embryogenesis." Biochem J 1997 May 15,324(Pt 1):209-16
T1730	NM_007741	Col9a2	PARTIAL CONCORDANCE	Perala M, Savontaus M, Metsaranta M, Vuorio E. "Developmental regulation of mRNA species for types II, IX and XI collagens during mouse embryogenesis." Biochem J 1997 May 15,324(Pt 1):209-16
T4617	NM_009936	Col9a3	CONCORDANCE	Perala M, Savontaus M, Metsaranta M, Vuorio E. "Developmental regulation of mRNA species for types II, IX and XI collagens during mouse embryogenesis." Biochem J 1997 May 15,324(Pt 1):209-16
T30201	NM_007770	Crx	CONCORDANCE	Chen S, Wang QL, Nie Z, Sun H, Lennon G, Copeland NG, Gilbert DJ, Jenkins NA, Zack DJ. "Crx, a novel Otx-like paired-homeodomain protein, binds to and transactivates photoreceptor cell-specific genes." Neuron 1997 Nov,19(5):1017-30
T1845	NM_009965	Cryba1	NP	
T36200	NM_007773	Crybb2	NP	
T9121	NM_007774	Cryga	CONCORDANCE	Santhiya ST, Abd-alla SM, Loster J, Graw J. "Reduced levels of gamma-crystallin transcripts during embryonic development of murine Cat2nop mutant lenses." Graefes Arch Clin Exp Ophthalmol 1995 Dec,233(12):795-800
T36210	NM_013808	Csrp3	NP	
T3071	NM_173369	Cyld	NP	
T50311	NM_010055	Dlx3	CONCORDANCE	Robinson GW, Mahon KA. "Differential and overlapping expression domains of Dlx-2 and Dlx-3 suggest distinct roles for Distal-less homeobox genes in craniofacial development." Mech Dev 1994 Dec,48(3):199-215
T8112	NM_010100	Edar	CONCORDANCE	Aberg T, Wang XP, Kim JH, Yamashiro T, Bei M, Rice R, Ryoo HM, Thesleff I. "Runx2 mediates FGF signaling from epithelium to mesenchyme during tooth morphogenesis." Dev Biol 2004 Jun 1,270(1):76-93
T8244	NM_175746	Edaradd	CONCORDANCE	Headon DJ, Emmal SA, Ferguson BM, Tucker AS, Justice MJ, Sharpe PT, Zonana J. "Overbeek PA, Gene defect in ectodermal dysplasia implicates a death domain adapter in development." Nature 2001 Dec 20-27,414(6866):913-6
T50378	NM_010110	Efnb1	CONCORDANCE	Bouillet P, Oulad-Abdelghani M, Vicaire S, Garnier JM, Schuhbaur B, Dolle P, Chambon P. "Efficient cloning of cDNAs of retinoic acid-responsive genes in P19 embryonal carcinoma cells and characterization of a novel mouse gene, Stra1 (mouse LERK-2/Eplg2)." Dev Biol 1995 Aug,170(2):420-33
T6240	NM_010162	Ext1	NP	
T3044	NM_011812	Fbln5	CONCORDANCE	Nakamura T, Ruiz-Lozano P, Lindner V, Yabe D, Taniwaki M, Furukawa Y, Kobuke K, Tashiro K, Lu Z, Andon NL, Schaub R, Matsumori A, Sasayama S, Chien KR, Honjo T. "DANCE, a novel secreted RGD protein expressed in developing, atherosclerotic, and balloon-injured arteries." J Biol Chem 1999 Aug 6,274(32):22476-83
T6765	NM_008001	Fgd1	CONCORDANCE	Gorski JL, Estrada L, Hu C, Liu Z. "Skeletal-specific expression of fgd1 during bone formation and skeletal defects in faciogenital dysplasia (FGDY, aarskog syndrome)." Dev Dyn 2000 Aug,218(4):573-86
T39648	XM_977402	Flnb	CONCORDANCE	Sheen VL, Feng Y, Graham D, Takafuta T, Shapiro SS, Walsh CA. "Filamin A and Filamin B are co-expressed within neurons during periods of neuronal migration and can physically interact." Hum Mol Genet 2002 Nov 1,11(23):2845-54
T36392	NM_175473	Fras1	PARTIAL CONCORDANCE	Petrou P, Pavlakis E, Dalezios Y, Chalepakis G. "Basement membrane localization of Frem3 is independent of the Fras1/Frem1/Frem2 protein complex within the sublamina densa." Matrix Biol 2007 Oct,26(8):652-8
T38972	NM_172862	Frem2	CONCORDANCE	Petrou P, Pavlakis E, Dalezios Y, Chalepakis G. "Basement membrane localization of Frem3 is independent of the Fras1/Frem1/Frem2 protein complex within the sublamina densa." Matrix Biol 2007 Oct,26(8):652-8
T35447	NM_008092	Gata4	CONCORDANCE	Jacobsen CM, Narita N, Bielinska M, Syder AJ, Gordon JI, Wilson DB. "Genetic mosaic analysis reveals that GATA-4 is required for proper differentiation of mouse gastric epithelium." Dev Biol 2002 Jan 1,241(1):34-46
T6369	NM_008109	Gdf5	CONCORDANCE	Kunath M, Ludecke HJ, Vortkamp A. "Expression of Trps1 during mouse embryonic development." Mech Dev 2002 Dec,119 Suppl 1():S117-20
T35451	XM_136212	Gli2	NO CONCORDANCE (apparently): incomplete analysis	Hardcastle Z, Mo R, Hui CC, Sharpe PT. "The Shh signalling pathway in tooth development: defects in Gli2 and Gli3 mutants." Development 1998 Aug,125(15):2803-11
T50449	NM_008130	Gli3	PARTIAL CONCORDANCE	Hui CC, Joyner AL. "A mouse model of greig cephalopolysyndactyly syndrome: the extra-toesJ mutation contains an intragenic deletion of the Gli3 gene." Nat Genet 1993 Mar,3(3):241-6
T4529	NM_016697	Gpc3	PARTIAL CONCORDANCE	Pellegrini M, Pilia G, Pantano S, Lucchini F, Uda M, Fumi M, Cao A, Schlessinger D, Forabosco A. "Gpc3 expression correlates with the phenotype of the Simpson-Golabi-Behmel syndrome." Dev Dyn 1998 Dec,213(4):431-9
T1060	NM_018882	Gpr56	NP	

T5309	NM_008264	Hoxa13	CONCORDANCE	Warot X, Fromental-Ramain C, Fraulob V, Chambon P, Dolle P. "Gene dosage-dependent effects of the Hoxa-13 and Hoxd-13 mutations on morphogenesis of the terminal parts of the digestive and urogenital tracts." Development 1997 Dec,124(23):4781-91
T45097	NM_008275	Hoxd13	CONCORDANCE	Albrecht AN, Schwabe GC, Stricker S, Boddrich A, Wanker EE, Mundlos S. "The synpolydactyly homolog (spdh) mutation in the mouse -- a defect in patterning and growth of limb cartilage elements." Mech Dev 2002 Mar,112(1-2):53-67
T1663	NM_010544	Ihh	CONCORDANCE	Long F, Zhang XM, Karp S, Yang Y, McMahon AP. "Genetic manipulation of hedgehog signaling in the endochondral skeleton reveals a direct role in the regulation of chondrocyte proliferation." Development 2001 Dec,128(24):5099-108
T3295	NM_016851	Irf6	CONCORDANCE	Knight AS, Schutte BC, Jiang R, Dixon MJ."Developmental expression analysis of the mouse and chick orthologues of IRF6: The gene mutated in Van der Woude syndrome." Dev Dyn 2006 May,235(5):1441-7
T30696	NM_008397	Itga6	CONCORDANCE	Salmivirta K, Gullberg D, Hirsch E, Altruda F, Ekblom P. "Integrin subunit expression associated with epithelial-mesenchymal interactions during murine tooth development." Dev Dyn 1996 Feb,205(2):104-13
T3515	NM_133663	Itgb4	CONCORDANCE	Salmivirta K, Gullberg D, Hirsch E, Altruda F, Ekblom P. "Integrin subunit expression associated with epithelial-mesenchymal interactions during murine tooth development." Dev Dyn 1996 Feb,205(2):104-13
T4827	NM_027391	Iyd (0610009A07Rik)	NP	
T63337	NM_013822	Jag1	PARTIAL CONCORDANCE	Leimeister C, Schumacher N, Gessler M. "Expression of Notch pathway genes in the embryonic mouse metanephros suggests a role in proximal tubule development." Gene Expr Patterns 2003 Oct,3(5):595-8
T2297	NM_001002011	Lmna	NP	
T7624	NM_010770	Matn3	NP	
T38227	NM_010797	Mid1	CONCORDANCE	Dal Zotto L, Quaderi NA, Elliott R, Lingerfelter PA, Carrel L , Valsecchi V , Montini E , Yen CH , Chapman V , Kalcheva I , Arrigo G , Zuffardi O , Thomas S , Willard HF , Ballabio A , Disteche CM , Rugarli EI. "The mouse Mid1 gene: implications for the pathogenesis of Opitz syndrome and the evolution of the mammalian pseudoautosomal region." Hum Mol Genet 1998 Mar,7(3):489-99
T36632	NM_008607	Mmp13	PARTIAL CONCORDANCE	Richardson RJ, Dixon J, Jiang R, Dixon MJ. "Integration of IRF6 and Jagged2 signalling is essential for controlling palatal adhesion and fusion competence." Hum Mol Genet 2009 Jul 15,18(14):2632-42
T50722	NM_008709	Mycn	CONCORDANCE	Shimono A, Okuda T, Kondoh H. "N-myc-dependent repression of ndr1, a gene identified by direct subtraction of whole mouse embryo cDNAs between wild type and N-myc mutant." Mech Dev 1999 May,83(1-2):39-52
T50700	NM_010856	Myh6	CONCORDANCE	Jones WK, Sanchez A, Robbins J. "Murine pulmonary myocardium: developmental analysis of cardiac gene expression." Dev Dyn 1994 Jun,200(2):117-28
T9977	NM_010861	Myl2	CONCORDANCE	Wang Z, Zhai W, Richardson JA, Olson EN, Meneses JJ, Firpo MT, Kang C, Skarnes WC, Tjian R. "Polybromo protein BAF180 functions in mammalian cardiac chamber maturation." Genes Dev 2004 Dec 15,18(24):3106-16
T9966	NM_010859	Myl3	CONCORDANCE	Chuva de Sousa Lopes SM, Hassink RJ, Feijen A, van Rooijen MA, Doevendans PA, Tertoolen L, Brutel de la Riviere A, Mummery CL. "Patterning the heart, a template for human cardiomyocyte development." Dev Dyn 2006 Apr 28,235(7):1994-2002
T6555	NM_010877	Ncf2	NP	
T36683	NM_010907	Nfkbia	PARTIAL CONCORDANCE	Schmidt-Ullrich R, Tobin DJ, Lenhard D, Schneider P, Paus R, Scheidereit C." NF-{kappa}B transmits Eda A1/EdaR signalling to activate Shh and cyclin D1 expression, and controls post-initiation hair placode down growth." Development 2006 Mar,133(6):1045-57
T36692	NM_008711	Nog	PARTIAL CONCORDANCE	Minina E, Schneider S, Rosowski M, Lauster R, Vortkamp A, Expression of Fgf and Tgfbeta signaling related genes during embryonic endochondral ossification., Gene Expr Patterns 2005 Dec,6(1):102-109
T4975	NM_173788	Npr2	CONCORDANCE	DiCicco-Bloom E, Lelievre V, Zhou X, Rodriguez W, Tam J, Waschek JA. "Embryonic expression and multifunctional actions of the natriuretic peptides and receptors in the developing nervous system." Dev Biol 2004 Jul 1,271(1):161-75
T50755	NM_007430	Nr0b1	CONCORDANCE	Ikeda Y, Swain A, Weber TJ, Hentges KE, Zanaria E, Lalli E, Tamai KT , Sassone-Corsi P , Lovell-Badge R , Camerino G , Parker KL. "Steroidogenic factor 1 and Dax-1 colocalize in multiple cell lineages: potential links in endocrine development." Mol Endocrinol 1996 Oct,10(10):1261-72
T36726	NM_139310	Otoa	NP	
T4347	NM_144841	Otx2	CONCORDANCE	Rowan S, Chen CM, Young TL, Fisher DE, Cepko CL. "Transdifferentiation of the retina into pigmented cells in ocular retardation mice defines a new function of the homeodomain gene Chx10." Development 2004 Oct,131(20):5139-52
T4347	NM_144841	Otx2	CONCORDANCE	Rowan S, Chen CM, Young TL, Fisher DE, Cepko CL, Transdifferentiation of the retina into pigmented cells in ocular retardation mice defines a new function of the homeodomain gene Chx10., Development 2004 Oct,131(20):5139-52
T10059	NM_011864	Papss2	NP	
T36840	NM_011037	Pax2	CONCORDANCE	Dressler GR, Deutsch U, Chowdhury K, Nornes HO, Gruss P. "Pax2, a new murine paired-box-containing gene and its expression in the developing excretory system." Development 1990 Aug,109(4):787-95
T30426	NM_008781	Pax3	PARTIAL CONCORDANCE	Goulding MD, Chalepakis G, Deutsch U, Erselius JR, Gruss P, Pax-3, a novel murine DNA binding protein expressed during early neurogenesis., EMBO J 1991 May,10(5):1135-47
T50782	X63963	Pax6	CONCORDANCE	Pulkkinen MA, Spencer-Dene B, Dickson C, Otonkoski T. "The IIIb isoform of fibroblast growth factor receptor 2 is required for proper growth and branching of pancreatic ductal epithelium but not for differentiation of exocrine or endocrine cells." Mech Dev 2003 Feb,120(2):167-75
T7528	NM_011040	Pax8	CONCORDANCE	Meunier D, Aubin J, Jeannotte L. "Perturbed thyroid morphology and transient hypothyroidism symptoms in Hoxa5 mutant mice." Dev Dyn 2003 Jul,227(3):367-78
T3544	NM_175933	Pex5	NP	
T55145	NM_011098	Pitx2	CONCORDANCE	Martin DM, Skidmore JM, Fox SE, Gage PJ, Camper SA. "Pitx2 distinguishes subtypes of terminally differentiated neurons in the developing mouse neuroepithelium." Dev Biol 2002 Dec 1,252(1):84-99
T7331	NM_026651	Pomgnt1	NP	
T50868	NM_008901	Pou3f4	PARTIAL CONCORDANCE	Sornson MW, Wu W, Dasen JS, Flynn SE, Norman DJ, O'Connell SM, Gukovsky I, Carriere C, Ryan AK, Miller AP, Zuo L, Gleiberman AS, Andersen B, Beamer WG, Rosenfeld MG. "Pituitary lineage determination by the Prophet of Pit-1 homeodomain factor defective in Ames dwarfism." Nature 1996 Nov 28,384(6607):327-33
T36892	XM_355243	Prg4	NP	
T63072	NM_008957	Ptch1	CONCORDANCE	Hardcastle Z, Mo R, Hui CC, Sharpe PT. "The Shh signalling pathway in tooth development: defects in Gli2 and Gli3 mutants." Development 1998 Aug,125(15):2803-11

T30126	NM_011199	Pthr1	PARTIAL CONCORDANCE	Karperien M, van Dijk TB, Hoeijmakers T, Cremers F, Abou-Samra AB, Boonstra J, de Laat SW, Defize LH, Expression pattern of parathyroid hormone/parathyroid hormone related peptide receptor mRNA in mouse postimplantation embryos indicates involvement in multiple developmental processes., Mech Dev 1994 Jul,47(1):29-42
T36944	NM_009023	Rapsn	CONCORDANCE	Michalk A, Stricker S, Becker J, Rupps R, Pantzar T, Miertus J, Botta G, Naretto VG, Janetzki C, Yaqoob N, Ott CE, Seelow D, Wieczorek D, Fiebig B, Wirth B, Hoopmann M, Walther M, Korber F, Blankenburg M, Mundlos S, Heller R, Hoffmann K." Acetylcholine receptor pathway mutations explain various fetal akinesia deformation sequence disorders." Am J Hum Genet 2008 Feb,82(2):464-76
T5407	NM_013833	Rax	CONCORDANCE	Shimogori T; Lee DA; Miranda-Angulo A; Yang Y; Wang H; Jiang L. Yoshida AC; Kataoka A; Mashiko H; Avetisyan M; Qi L; Qian J; Blackshaw S. A genomic atlas of mouse hypothalamic development. Nat Neurosci 2010 Jun;13(6):767-75.
T1490	NM_021390	Sall1	CONCORDANCE	Buck A, Kispert A, Kohlhase J. "Embryonic expression of the murine homologue of SALL1, the gene mutated in Townes-Brocks syndrome." Mech Dev 2001 Jun,104(1-2):143-6
T51007	X76290	Shh	CONCORDANCE	Aberg T, Wang XP, Kim JH, Yamashiro T, Bei M, Rice R, Ryoo HM, Thesleff I, Runx2 mediates FGF signaling from epithelium to mesenchyme during tooth morphogenesis., Dev Biol 2004 Jun 1,270(1):76-93
T55175	NM_023990	Six3	CONCORDANCE	Oliver G, Mailhos A, Wehr R, Copeland NG, Jenkins NA, Gruss P. "Six3, a murine homologue of the sine oculis gene, demarcates the most anterior border of the developing neural plate and is expressed during eye development." Development 1995 Dec,121(12):4045-55
T52	NM_007885	Slc26a2	NP	
T5186	NM_011448	Sox9	CONCORDANCE	Lioubinski O, Muller M, Wegner M, Sander M. "Expression of Sox transcription factors in the developing mouse pancreas." Dev Dyn 2003 Jul,227(3):402-8
T63422	NM_009291	Stra6	NP	
T2550	NM_011530	Tap2	NP	
T35545	NM_032005	Tbx19	CONCORDANCE	Liu J, Lin C, Gleiberman A, Ohgi KA, Herman T, Huang HP, Tsai MJ, Rosenfeld MG. "Tbx19, a tissue-selective regulator of POMC gene expression." Proc Natl Acad Sci U S A 2001 Jul 17,98(15):8674-9
T38911	NM_194263	Tbx20	CONCORDANCE	Mommersteeg MT, Brown NA, Prall OW, de Gier-de Vries C, Harvey RP, Moorman AF, Christoffels VM, Pitx2c and Nkx2-5 are required for the formation and identity of the pulmonary myocardium., Circ Res 2007 Oct 26,101(9):902-9
T38116	NM_011535	Tbx3	CONCORDANCE	Gibson-Brown JJ, Mech Dev 1996 May;56(1-2):93-101
T37121	NM_009347	Tecta	CONCORDANCE	Rau A, Legan PK, Richardson GP." Tectorin mRNA expression is spatially and temporally restricted during mouse inner ear development." J Comp Neurol 1999 Mar 8,405(2):271-80
T6462	NM_011577	Tgfb1	CONCORDANCE	Aberg T, Wang XP, Kim JH, Yamashiro T, Bei M, Rice R, Ryoo HM, Thesleff I, Runx2 mediates FGF signaling from epithelium to mesenchyme during tooth morphogenesis., Dev Biol 2004 Jun 1,270(1):76-93
T3076	NM_009372	Tgif	NP	
T4020	NM_009405	Tnni2	NP	
T9973	NM_009406	Tnni3	CONCORDANCE	Mommersteeg MT, Brown NA, Prall OW, de Gier-de Vries C, Harvey RP, Moorman AF, Christoffels VM. "Pitx2c and Nkx2-5 are required for the formation and identity of the pulmonary myocardium." Circ Res 2007 Oct 26,101(9):902-9
T3683	NM_011619	Tnnt2	NP	
T6295	NM_011620	Tnnt3	NP	
T2646	NM_022017	Trpv4	NP	
T38075	NM_011652	Ttn	NP	
T38083	NM_023478	Upk3a	NP	
**Notes: NP- not present in GXD database**				

Notes

			NB STUDY IS IN THE EYE			
			NB sections AT 13.5			

				NB STUDY ON TEETH		
				NB study is on placenta		
				NB in both references not specific of liver,2 probes in EE, 1 not annotated yet		
			NB NOT liver specific in visel AND GP,2 assays in EE, 1 not annotated resembling GP			

			Jernvall J, Development 1998 Jan;125(2):161-9		

				Dacquin R, Dev Dyn 2004 Apr;229(4):826-34		
				Thut CJ, Dev Biol 2001 Mar 1;231(1):63-76		
				Headon DJ, Nature 2001 Dec 20-27;414(6866):913-6		

				Rice R, Gene Expr Patterns 2006 Jan;6(2):206-12		
					Le TT, Dev Biol 2009 Apr 1;328(1):118-26	
			Brain Res Mol Brain Res 2004 Dec 20;132(2):116-27			
				Nornes HO, Development 1990 Aug;109(4):797-809		
				Rowan S, Development 2004 Oct;131(20):5139-52		
			Dev Dyn 2007 May;236(5):1307-12			

				Long F, Development 2001 Dec;128(24): 5099-108		
			Development 1998 Aug;125(15):2803-11			
			Development 2008 Sep;135(18):3113-24			
				Minina E, Gene Expr Patterns 2005 Dec;6(1):102 109		
