# Supplementary material for: A High-Resolution Anatomical Atlas of the Transcriptome in the Mouse Embryo
Source: PLoS Biol. 2011 Jan 18;9(1):e1000582. doi: 10.1371/journal.pbio.1000582 (PMC3022534; doi:10.1371/journal.pbio.1000582)
Supplement: Table S3 — List of genes that display exclusive expression in selected structures. (0.10 MB PDF) [file pbio.1000582.s011.pdf]

**Table S3**. List of genes that display exclusive expression in selected structures			
TISSUE	Template ID	RefSeq accession	Gene symbol
CENTRAL NERVOUS SYSTEM			
Cerebral cortex	T1569	NM_023906	Asb3
Cerebral cortex	T4116	NM_133733	9030425E11Rik
Cerebral cortex	T5339	NM_133190	Cacng8
Cerebral cortex	T7613	NM_133750	3110048E14Rik
Cerebral cortex	T8641	NM_013581	Cog1
Cerebral cortex	T8887	NM_008151	Gpr12
Cerebral cortex	T7122	NM_016736	6330412F12Rik (Nub1)
Cerebral cortex	T35200	NM_177383	Gpr21
Cerebral cortex	T2778	NM_010903	Nfe2l3
Cerebral cortex	T35433	NM_175647	Dmrta1
Cerebral cortex	T35847	XR_005251	A930024E05Rik
Cerebral cortex	T36070	XM_355752	Dgki
Cerebral cortex	T9316	NM_027135	Sec24d
Cerebral cortex	T31018	NM_021499	Wdr8
Cerebral cortex	T31985	NM_027260	Vrk2
Cerebral cortex	T30457	NM_008992	Abcd4
Cerebral cortex	T37784	NM_080795	Lnx2
Cerebral cortex	T40492	AK018398	8430408J09Rik
Cerebral cortex	T45541	NM_001014390	Dyrk2
Cerebral cortex	T70151	MIMAT0003491	mmu-miR-701
Corpus striatum	T36964	NM_172516	Ripk5
Thalamus	T3840	NM_018884	Pdzrn3
Thalamus	T2359	NM_023850	Chst1
Thalamus	T4098	NM_172310	Tarsl2
Thalamus	T7610	NM_178891	Hrmt1l6
Thalamus	T35194	NM_181543	Gpr151
Hypothalamus	T36957	NM_021892	Rfrp
Hypothalamus	T38159	NM_177892	A230097C02
Hypothalamus	T30773	NM_009383	Tial1
Midbrain	T8662	NM_178625	2700094F01Rik
Midbrain	T35706	NM_027760	Rassf8
Midbrain	T36162	NM_153105	Cldn19
Midbrain	T37488	NM_175651	9630008K15Rik
Midbrain	T36121	NM_175631	Cbln4
Midbrain	T31417	NM_029945	Smpd4
Midbrain	T2314	NM_025897	1500003O22Rik
Midbrain	T38213	NM_028065	Tnrc5
Midbrain	T40280	AK077915	Mipol1
Midbrain	T45629	XM_973549	4930589M24Rik
Midbrain	T45132	XM_890447	Mllt4
Cerebellum	T6536	NM_028184	Oraov1
Cerebellum	T35739	NM_027519	6330406I15Rik
Cerebellum	T36476	NM_015820	Hs6st3
Cerebellum	T37027	NM_009200	Slc1a6
Cerebellum	T31051	NM_029933	Bcl9
Cerebellum	T63352	NM_207237	Man1c1
Cerebellum	T63289	NM_198678	Sh3md2
Pons	T35109	NM_009766	Brs3
Pons	T9940	NM_026925	Pnlip
Pons	T2618	NM_022408	Es2el
Pons	T36864	NM_001001320	Tbx10
Pons	T38784	NM_008022	Foxd4
Pons	T40239	XM_989876	4631416L12Rik
Medulla oblongata	T3799	NM_023438	Gm644
Medulla oblongata	T5619	NM_026012	Nradd
Medulla oblongata	T35259	NM_013582	Lhcgr
Medulla oblongata	T35827	NM_177358	A630033E08Rik
Medulla oblongata	T35828	NM_177255	A630052C17Rik
Medulla oblongata	T36821	XM_486090	Nkx6-3
Medulla oblongata	T36197	NM_170597	Creg2
Medulla oblongata	T31600	NM_181315	Car5b
Medulla oblongata	T31319	NM_028248	2610301K12Rik
Medulla oblongata	T38668	NM_010586	Itpr2
Spinal cord	T5360	NM_008265	Hoxa4
Spinal cord	T5313	NM_010454	Hoxa6
Spinal cord	T3054	NM_144544	2210407C18Rik
Spinal cord	T36034	NM_181422	Pkd2l1
Spinal cord	T36325	NM_139293	Ece2
Spinal cord	T35172	NM_177330	Ghsr
Spinal cord	T35853	NM_175352	AA407659
Spinal cord	T37251	NM_027401	1700010C24Rik
Spinal cord	T30543	NM_016663	Syt3
Spinal cord	T38441	NM_008226	Hcn2
Spinal cord	T37397	XM_916223	4931429I11Rik
Spinal cord	T31587	NM_026013	Tmem77
Spinal cord	T38192	NM_138948	Cabp7
Spinal cord	T40298	AK015488	4930459I23Rik
Spinal cord	T39753	AK083519	D030040B21
Spinal cord	T45276	NM_145579	P42pop
Spinal cord	T70096	MIMAT0005292	mmu-miR-582-3p
Spinal cord	T70406	MIMAT0000567	mmu-miR-329

Cranial ganglia	T4202	NM_028207	Dusp3
Cranial ganglia	T632	NM_029031	Carkl
Cranial ganglia	T4705	NM_019443	Ndufa1
Cranial ganglia	T5592	NM_176848	Fbxo2
Cranial ganglia	T35378	NM_175198	1700058C01Rik
Cranial ganglia	T35883	NM_009620	Adam4
Cranial ganglia	T36751	NM_053144	Pcdhb19
Cranial ganglia	T36312	XM_917279	Layn
Cranial ganglia	T35839	NM_177045	Cc2d1b
Cranial ganglia	T36635	NM_001033339	Mmp25
Cranial ganglia	T37022	NM_011390	Slc12a7
Cranial ganglia	T30923	NM_138650	Dgkg
Cranial ganglia	T32139	NM_133914	Rasa4
Cranial ganglia	T37566	NM_181728	Art3
Cranial ganglia	T39008	NM_178651	Slc30a9
Cranial ganglia	T39034	NM_021332	Glp1r
Cranial ganglia	T37560	NM_177781	Trpa1
Cranial ganglia	T30023	NM_009831	Ccng1
Cranial ganglia	T37051	NM_172841	Slco5a1
Cranial ganglia	T37651	NM_029879	D13Bwg1146e
Cranial ganglia	T31259	NM_145497	BC016495
Cranial ganglia	T37063	NM_198214	Snph
Cranial ganglia	T40474	AK083647	D030059C06Rik
Cranial ganglia	T40506	AK004446	1190002E22Rik
Cranial ganglia	T35036	XM_134539	2310031A18Rik
PERIPHERAL NERVOUS SYSTEM			
Sympathetic ganglia	T3272	NM_012026	Rgnef
Sympathetic ganglia	T8010	NM_008213	Hand1
Sympathetic ganglia	T3514	NM_172451	Galnt6
Sympathetic ganglia	T35962	NM_009726	Atp7a
Dorsal root ganglia	T1499	NM_177226	Zfp629
Dorsal root ganglia	T3628	NM_144817	Camk1g
Dorsal root ganglia	T3678	NM_134022	6330403K07Rik
Dorsal root ganglia	T1199	NM_019806	Vapb
Dorsal root ganglia	T5536	NM_023397	1810034K20Rik
Dorsal root ganglia	T3282	NM_026552	Arpc4
Dorsal root ganglia	T3389	NM_025947	Dncl2a
Dorsal root ganglia	T3378	NM_177262	Pkn1
Dorsal root ganglia	T7848	NM_178358	Lhfpl1
Dorsal root ganglia	T3053	NM_009894	Cideb
Dorsal root ganglia	T35608	NM_026622	3110057O12Rik
Dorsal root ganglia	T36442	NM_010305	Gnai1
Dorsal root ganglia	T45437	NM_199365	5330438I03Rik
Dorsal root ganglia	T70297	MIMAT0000215	mmu-miR-186
ALIMENTARY SYSTEM			
Salivary glands	T992	NM_026009	2610204L23Rik
Salivary glands	T4565	NM_025884	Ccdc16
Salivary glands	T4570	NM_027231	Polr2f
Salivary glands	T1153	NM_126166	Tlr3
Salivary glands	T6034	NM_025297	Nrbf1
Salivary glands	T5194	NM_025920	Thap4
Salivary glands	T3931	NM_019870	Ard1
Salivary glands	T994	NM_023167	Mrpl4
Salivary glands	T1053	NM_007991	Fbl
Salivary glands	T4266	NM_181410	Gtf2h3
Salivary glands	T4653	NM_146238	BC023488
Salivary glands	T4654	NM_025644	Exosc1
Salivary glands	T4649	NM_025298	Polr3e
Salivary glands	T4839	NM_020483	Hcngp
Salivary glands	T4699	NM_026421	2310057D15Rik
Salivary glands	T4918	NM_172049	Tmem18
Salivary glands	T6193	NM_144829	2310044P18Rik
Salivary glands	T6824	NM_133756	Xab1
Salivary glands	T6826	NM_025567	Cyc1
Salivary glands	T6808	NM_145386	BC005655
Salivary glands	T8575	NM_029418	9130401M01Rik
Salivary glands	T8298	NM_053113	Ear11
Salivary glands	T35764	NM_177136	9030227G01Rik
Salivary glands	T7090	NM_053168	Trim11
Salivary glands	T7340	NM_019910	Dcpp
Salivary glands	T8417	NM_026744	Mrpl53
Salivary glands	T36466	NM_010411	Hdac3
Salivary glands	T31763	NM_023197	2310008H09Rik
Salivary glands	T36482	NM_010489	Hyal2
Salivary glands	T30449	NM_028604	2410075D05Rik
Salivary glands	T45583	NM_028274	Exosc6
Salivary glands	T39150	NM_053170	Trim33
Salivary glands	T38409	XM_109726	Thrap1
Salivary glands	T39614	Y15800	Gprk6
Salivary glands	T40565	NM_001040026	Sco1
Salivary glands	T40143	AK013632	LOC434446
Salivary glands	T40477	AK156793	Dnajc11
Salivary glands	T63207	NM_178640	B3galnt2
Salivary glands	T63369	NM_207239	Gtf3c1

pharynx	T35071	XM_135805	Wdr44
pharynx	T7055	NM_146236	Tceal1
pharynx	T37506	NM_175511	A130092J06Rik
pharynx	T37713	NM_027402	Fndc5
pharynx	T39931	AK044039	LOC432809
Oesophagus	T2189	NM_029320	D14Ertd581e
Oesophagus	T30549	NM_178711	Plscr4
Oesophagus	T30424	NM_148942	Serpinb6c
Stomach	T3497	NM_001012392	U46068
Stomach	T5284	NM_145469	9330161F08Rik
Stomach	T35959	NM_009724	Atp4b
Stomach	T37359	NM_030181	Vsig1
Stomach	T37452	NM_025684	5730521E12Rik
Stomach	T37039	NM_053248	Slc5a5
Stomach	T31264	NM_027299	Degs2
Stomach	T37839	NM_001033366	Dpcr1
Intestine	T6200	NM_010893	Neu1
Intestine	T8444	NM_138646	Hps4
Intestine	T7375	NM_178308	Abpg
Intestine	T4828	NM_145932	D630035O19Rik
Intestine	T35459	NM_013920	Hnf4g
Intestine	T36820	XM_134330	Isx
Intestine	T36216	XM_130038	Cubn
Intestine	T36195	XM_129769	Cps1
Intestine	T31175	NM_010739	Muc13
Intestine	T31255	NM_144807	Chpt1
Intestine	T38238	NM_027339	2210415F13Rik
Intestine	T37860	NM_001011873	Xkr9
Intestine	T39400	XR_003553	LOC671753
Intestine	T2987	NM_027307	Golph2
Intestine	T2287	NM_028189	B3gnt3
Intestine	T32098	NM_007769	Dmbt1
Intestine	T37786	XM_923886	Gm53
Liver	T1660	NM_011280	Trim10
Liver	T452	NM_146173	1300010A20Rik
Liver	T3791	NM_153567	5033405K12Rik
Liver	T800	NM_172759	Ces5
Liver	T855	NM_009127	Scd1
Liver	T518	NM_008934	Proc
Liver	T887	NM_010168	F2
Liver	T867	NM_133977	Trf
Liver	T1441	NM_008777	Pah
Liver	T113	NM_011270	Rhced
Liver	T928	NM_023383	Aadac
Liver	T882	NM_008124	Gjb1
Liver	T1032	NM_008878	Serpinf2
Liver	T3836	NM_138678	Butr1
Liver	T1017	NM_013782	Ptdss2
Liver	T3940	NM_019687	Slc22a4
Liver	T4696	NM_007931	Endog
Liver	T2929	NM_020583	Isg20
Liver	T2794	NM_008198	H2-Bf
Liver	T4763	NM_172577	Slc25a21
Liver	T4783	NM_010006	Cyp2d9
Liver	T4794	NM_146148	C8a
Liver	T4813	NM_174870	Slc26a1
Liver	T4015	NM_029269	Spp2
Liver	T1352	NM_174846	6230410P16Rik
Liver	T3113	NM_008385	Inpp5b
Liver	T4399	NM_016956	Hbb-b2
Liver	T4928	NM_008280	Lipc
Liver	T4954	NM_009778	C3
Liver	T4955	NM_007376	Pzp
Liver	T705	NM_013474	Apoa2
Liver	T647	NM_009695	Apoc2
Liver	T715	NM_023530	Pla2g12b
Liver	T5324	NM_001033981	LOC544763
Liver	T5508	NM_025988	Acbd4
Liver	T6010	NM_013551	Hmbs
Liver	T6020	NM_010196	Fga
Liver	T1930	NM_145824	Ranbp10
Liver	T914	NM_013475	Apoh
Liver	T201	NM_153143	Kctd11
Liver	T1576	NM_026157	Papd1
Liver	T4729	NM_017366	Acadvl
Liver	T4876	NM_144916	BC014685
Liver	T4914	NM_133997	Apof
Liver	T4916	NM_144869	BC021614
Liver	T4901	NM_021489	F12
Liver	T4900	NM_018816	Apom
Liver	T918	NM_009060	Rgn
Liver	T6813	NM_011787	Amfr
Liver	T6738	NM_011804	Creg1
Liver	T7396	NM_028066	F11

Liver	T6633	NM_144533	Nmnat3
Liver	T7241	NM_008341	Igfbp1
Liver	T4958	NM_019447	Hgfac
Liver	T4997	NM_144805	Tmem40
Liver	T4937	NM_080844	Serpinc1
Liver	T4953	NM_130452	Bbox1
Liver	T7876	NM_013711	Txnrd2
Liver	T6096	NM_144923	Blvrb
Liver	T7438	NM_017371	Hpxn
Liver	T7460	NM_022884	Bhmt2
Liver	T7462	NM_008439	Khk
Liver	T8819	NM_172406	Als2cr3
Liver	T8827	NM_013848	Ermap
Liver	T8327	NM_011414	Slpi
Liver	T9595	NM_207204	4930519N13Rik
Liver	T6101	NM_009775	Bzrp
Liver	T7233	NM_009690	Cd5l
Liver	T2656	NM_013484	C2
Liver	T9775	NM_053149	Hemgn
Liver	T10041	NM_008223	Serpind1
Liver	T9535	NM_009479	Uros
Liver	T103	NM_145365	Creb3l3
Liver	T544	NM_010799	Minpp1
Liver	T659	NM_012053	Rpl8
Liver	T572	NM_031164	F13b
Liver	T663	NM_008406	Itih1
Liver	T6652	NM_026635	5730536A07Rik
Liver	T8083	NM_008114	Gfi1b
Liver	T7377	NM_011822	Pigq
Liver	T7388	NM_008277	Hpd
Liver	T7065	NM_027868	Slc41a3
Liver	T6647	NM_008522	Ltf
Liver	T6734	NM_019687	Slc22a4
Liver	T4533	NM_013547	Hgd
Liver	T50012	NM_010509	Ifnar2
Liver	T3028	NM_007563	Bpgm
Liver	T35297	NM_011465	Spna1
Liver	T35306	NM_013838	Trpc6
Liver	T35925	NM_007440	Alox12
Liver	T36774	NM_008823	Cfp
Liver	T1370	NM_007940	Ephx2
Liver	T2632	NM_134249	Timd2
Liver	T36344	NM_013513	Epb4.2
Liver	T36357	NM_007976	F5
Liver	T36359	NM_176935	F730015K02Rik
Liver	T35159	NM_007975	F2rl3
Liver	T35249	NM_010605	Kcnj5
Liver	T35250	NM_001033525	Kcnk6
Liver	T35158	NM_010170	F2rl2
Liver	T1306	NM_008877	Plg
Liver	T1305	NM_026673	0610008C08Rik
Liver	T36422	NM_008129	Gclm
Liver	T36685	NM_008694	Ngp
Liver	T36891	NM_008920	Prg2
Liver	T36894	NM_016781	Prkag1
Liver	T36220	NM_007807	Cybb
Liver	T36962	NM_011269	Rhag
Liver	T36371	NM_030236	Fbxo34
Liver	T37562	XM_139463	Apol2
Liver	T30515	NM_010815	Grap2
Liver	T30513	NM_026464	Wdr55
Liver	T30262	NM_009146	Frrs1
Liver	T30528	NM_011461	Spic
Liver	T30861	NM_008525	Alad
Liver	T31177	NM_177564	BC022224
Liver	T31242	NM_181849	Fgb
Liver	T31238	NM_010531	Il18bp
Liver	T36214	NM_007800	Ctsg
Liver	T36584	NM_008491	Lcn2
Liver	T36625	NM_008572	Mcpt8
Liver	T37518	NM_177115	37682
Liver	T37548	NM_026212	Agpat2
Liver	T30120	NM_016751	Clec4f
Liver	T30968	NM_023737	Ehhadh
Liver	T31016	NM_027062	C8g
Liver	T31697	NM_178713	Aldh8a1
Liver	T37082	NM_138673	Stab2
Liver	T36464	NM_175000	Hbq1
Liver	T37646	NM_023785	Cxcl7
Liver	T31265	NM_178936	Tmem56
Liver	T38059	NM_178931	Tnfrsf14
Liver	T31407	NM_008254	Hmgcl
Liver	T38067	NM_027763	Treml1
Liver	T38095	NM_023500	Xkh

Liver	T31621	NM_181420	BC032265
Liver	T39081	NM_133246	Ms4a3
Liver	T39334	XM_914077	LOC545486
Liver	T39919	NM_001033488	Gm1964
Liver	T37793	XM_001002626	Gm323
Liver	T31656	NM_019949	Ube2l6
Liver	T39636	NM_013730	Slamf1
Liver	T37994	NM_173051	Serpinb1c
Liver	T63033	NM_010575	Itga2b
Liver	T45073	NM_010190	Fcnb
Liver	T45189	S66283	Spnb1
Liver	T70034	MIMAT0004870	mmu-miR-453
RESPIRATORY SYSTEM			
Trachea	T6962	NM_133993	Pwp1
Lung	T6275	NM_080434	Apoa5
Lung	T4913	NM_054094	Bucs1
Lung	T6471	NM_019734	Asah1
Lung	T6515	NM_026434	Rbm18
Lung	T6164	NM_145442	Mbip
Lung	T2760	NM_172372	Wdr45
Lung	T8173	NM_026812	1110033O09Rik
Lung	T7968	NM_028798	2300002G24Rik
Lung	T8216	NM_008403	Itgb1bp1
Lung	T9979	NM_011359	Sftpc
Lung	T48	NM_011247	Rbbp6
Lung	T36440	XM_977462	Gm632
Lung	T36888	NM_138605	Ppp1r3f
Lung	T36222	NM_007814	Cyp2b19
Lung	T37377	XM_485433	4930544O15Rik
Lung	T37174	NM_009417	Tpo
Lung	T37797	NM_001033246	Gm172
Lung	T36519	NM_172161	Irak2
Lung	T39769	AK034327	9330178D15
Lung	T472	NM_026210	1810036I24Rik
CARDIOVASCULAR SYSTEM			
Heart	T1670	NM_013593	Mb
Heart	T2081	NM_146242	Lrrc10
Heart	T1653	NM_008726	Nppb
Heart	T5933	NM_008590	Mest
Heart	T7325	NM_008424	Kcne1
Heart	T4941	NM_026977	1810031K17Rik
Heart	T4371	NM_145367	Txndc5
Heart	T4400	NM_011366	Sh3d4
Heart	T6397	NM_013483	Btn1a1
Heart	T7934	NM_011658	Twist1
Heart	T9824	NM_023129	Pln
Heart	T4377	NM_153805	Pkn3
Heart	T10016	NM_008725	Nppa
Heart	T10015	NM_013868	Hspb7
Heart	T9977	NM_010861	Myl2
Heart	T9973	NM_009406	Tnni3
Heart	T9980	NM_022879	Myl7
Heart	T35348	NM_026831	Mybphl
Heart	T45	NM_028990	8430437G11Rik
Heart	T217	NM_019750	Nat6
Heart	T186	NM_009400	Tnfrsf18
Heart	T123	NM_017470	Dnalc4
Heart	T1523	NM_178641	Inpp5f
Heart	T303	NM_021443	Ccl8
Heart	T1567	NM_172383	6330530A05Rik
Heart	T30118	NM_008965	Ptger4
Heart	T37660	NM_001033129	D6Ertd474e
Heart	T31276	NM_145499	Cyp2c70
Heart	T31371	NM_007982	Ptk2
Heart	T31391	NM_053115	Acox2
Heart	T31387	NM_016922	Gal3st1
Heart	T31390	NM_145597	Tmem161a
Heart	T31333	XM_001003747	2010321M09Rik
Heart	T31354	NM_010949	Numb
Heart	T31310	XM_001002673	0610007L01Rik
Heart	T38248	NM_134048	Cbll1
Heart	T39138	NM_146417	Olfr877
Heart	T38777	NM_001004162	LOC432436
Heart	T39388	AJ278965	Cd80
Heart	T40560	XM_905672	Apold1
Heart	T38815	NM_139306	Asah3l
LIMBS			
Limbs	T1777	NM_010827	Msc
Limbs	T3345	NM_029285	1700001C02Rik
Limbs	T6874	NM_011905	Tlr2
Limbs	T35464	NM_008274	Hoxd12
Limbs	T580	NM_019950	Chst5
Limbs	T36606	NM_010745	Ly86
Limbs	T36623	NM_133197	Mcf2

Limbs	T37492	NM_172904	Fsd2
Limbs	T63298	NM_026866	Disp1
Limbs	T70079	MIMAT0004940	mmu-miR-511
Limbs	T70081	MIMAT0002889	mmu-miR-532-5p
SKELETON			
Skeleton	T3537	NM_198703	Prkwnk1
Skeleton	T2205	NM_144828	Ppp1r1b
Skeleton	T1700	NM_177693	Lim2
Skeleton	T1555	NM_020625	Zfp297
Skeleton	T3639	NM_023243	Ccnh
Skeleton	T3619	NM_139307	Slitl2
Skeleton	T814	NM_013664	Sh3gl1
Skeleton	T858	NM_025388	Ufc1
Skeleton	T4520	NM_178758	C730027J19Rik
Skeleton	T2709	NM_019575	Scamp4
Skeleton	T4381	NM_133831	Gltscr2
Skeleton	T4425	NM_026601	Hyi
Skeleton	T5336	NM_147097	Olfr628
Skeleton	T6062	NM_138668	1810047C23Rik
Skeleton	T3420	NM_025448	Ssr2
Skeleton	T2050	NM_010098	Opn3
Skeleton	T5133	NM_172284	Ddx19b
Skeleton	T5180	NM_134058	Pelo
Skeleton	T5899	NM_053092	Kars
Skeleton	T2289	NM_025395	Chchd5
Skeleton	T4713	NM_025974	Rpl14
Skeleton	T4905	NM_172935	5730457F11Rik
Skeleton	T1774	NM_009666	Amelx
Skeleton	T6421	NM_009046	Relb
Skeleton	T5752	NM_025903	Ifrd2
Skeleton	T5801	NM_011970	Psmb2
Skeleton	T7659	NM_025872	Golt1b
Skeleton	T9604	NM_015781	Nap1l1
Skeleton	T4964	NM_028769	Syvn1
Skeleton	T2918	NM_011354	Serf2
Skeleton	T23	NM_144787	Jmjd2c
Skeleton	T2872	NM_026612	Ndufb2
Skeleton	T37220	NM_174877	Zar1
Skeleton	T30261	NM_016715	Tpte2
Skeleton	T37477	NM_001024926	Cyb5d2
Skeleton	T37148	NM_027865	Tmem25
Skeleton	T30672	NM_026000	Psmd9
Skeleton	T37052	NM_011408	Slfn2
Skeleton	T38197	NM_030137	Cstad
Skeleton	T39901	XM_894331	EG629441
Skeleton	T39900	AK038984	LOC432449
Skeleton	T31673	NM_007985	Fancc
Skeleton	T39412	NM_010748	Lyst
Skeleton	T31828	NM_025749	Zfp474
Skeleton	T39450	BC100377	AU022751
SKELETAL MUSCLE			
Skeletal muscle	T6124	NM_007386	Aco1
Skeletal muscle	T32146	NM_172872	Ankrd38
Skeletal muscle	T35071	XM_135805	Wdr44
Skeletal muscle	T7055	NM_146236	Tceal1
Skeletal muscle	T37506	NM_175511	A130092J06Rik
Skeletal muscle	T37713	NM_027402	Fndc5
Skeletal muscle	T5857	NM_134011	Tbrg4
Skeletal muscle	T6305	NM_175356	Pik4cb
Skeletal muscle	T6511	NM_021524	Pbef1
Skeletal muscle	T6529	NM_010209	Fh1
Skeletal muscle	T6124	NM_007386	Aco1
Skeletal muscle	T10017	NM_028115	Trub1
Skeletal muscle	T36425	NM_010834	Gdf8
Skeletal muscle	T2452	NM_009605	Adipoq
Skeletal muscle	T36831	NM_172585	Larp5
Skeletal muscle	T38370	XM_137117	Adamts14
Skeletal muscle	T40448	AK078202	6430502G17Rik
Skeletal muscle	T40349	BC027793	Scfd1
Skeletal muscle	T39628	XM_001004492	LOC677542
Skeletal muscle	T45652	XM_001003389	LOC676704
SKIN			
Skin	T1554	NM_026186	1300013D05Rik
Skin	T2218	NM_031998	Tsga14
Skin	T947	NM_020036	Calm4
Skin	T417	NM_010118	Egr2
Skin	T4450	NM_146063	BC031593
Skin	T2531	NM_007394	Acvr1
Skin	T3020	NM_025296	Wdr39
Skin	T3331	NM_028712	Rap2b
Skin	T3349	NM_009374	Tgm3
Skin	T1585	NM_029291	Ascc2
Skin	T6367	NM_007927	Emd
Skin	T6712	NM_146097	Cbwd1

Skin	T8259	NM_180588	2700029E10Rik
Skin	T5156	NM_172845	Adamts4
Skin	T7619	NM_029810	Nt5c2
Skin	T50010	NM_010456	Hoxa9
Skin	T50016	AB038697	Olig2
Skin	T373	NM_021450	Trpm7
Skin	T35716	XM_001000101	Tnks2
Skin	T37586	XM_893176	Znrf3
Skin	T37334	NM_001033397	Krt26
Skin	T36617	NM_016693	Map3k6
Skin	T36993	NM_022886	Scel
Skin	T30441	NM_178381	Trp53i5
Skin	T38055	NM_205820	Tlr13
Skin	T31298	NM_025911	Ccdc91
Skin	T31349	NM_029012	Sppl3
Skin	T31544	NM_025408	Phca
Skin	T38469	NM_001003911	Adamts7
Skin	T38458	XM_980092	Krt28
Skin	T38473	NM_213728	Krt72
Skin	T39062	NM_011310	S100a3
HAEMOLYMPHOID SYSTEM			
Thymus	T861	NM_013640	Psmb10
Thymus	T99	NM_025626	3110001A13Rik
Thymus	T4485	NM_026362	5033414D02Rik
Thymus	T4494	NM_013796	Nagpa
Thymus	T4542	NM_145139	Eif3s6ip
Thymus	T5657	NM_178698	Pigv
Thymus	T3123	NM_028126	2610019A05Rik
Thymus	T2708	NM_027185	Def6
Thymus	T2726	NM_023260	Mrps34
Thymus	T4411	NM_010545	Ii
Thymus	T2550	NM_011530	Tap2
Thymus	T4559	NM_009387	Tk1
Thymus	T1147	NM_009812	Casp8
Thymus	T4593	NM_146011	Arhgap9
Thymus	T4621	NM_023372	Rpl38
Thymus	T5480	NM_025624	2510048O06Rik
Thymus	T4631	NM_027139	Taf9
Thymus	T5489	NM_025907	1600013P15Rik
Thymus	T5499	NM_025582	2810405K02Rik
Thymus	T5515	NM_030711	Arts1
Thymus	T5538	NM_198031	Tubgcp3
Thymus	T3407	NM_010220	Fkbp5
Thymus	T1867	NM_010370	Gzma
Thymus	T5124	NM_146205	Armc5
Thymus	T5126	NM_153795	BC032204
Thymus	T2086	NM_172468	4732481H14Rik
Thymus	T5288	NM_053180	Ccrk
Thymus	T5298	NM_009761	Bnip3l
Thymus	T2148	NM_153788	Centb1
Thymus	T4840	NM_199016	Enpp4
Thymus	T4679	NM_026829	Mthfs
Thymus	T5383	NM_016684	Zfp96
Thymus	T5916	NM_134154	AW491445
Thymus	T6732	NM_010378	H2-Aa
Thymus	T6710	NM_026070	2900091E11Rik
Thymus	T6701	NM_010696	Lcp2
Thymus	T5700	NM_007497	Atf1
Thymus	T7039	NM_175397	5830484A20Rik
Thymus	T7306	NM_013566	Itgb7
Thymus	T6175	NM_024253	Nkg7
Thymus	T6132	NM_009151	Selpl
Thymus	T6119	NM_008279	Map4k1
Thymus	T6395	NM_022024	Gmfg
Thymus	T6429	NM_009277	Trim21
Thymus	T5756	NM_010379	H2-Ab1
Thymus	T6500	NM_021327	Tnip1
Thymus	T8062	NM_172435	P2ry10
Thymus	T8264	NM_013487	Cd3d
Thymus	T9559	NM_033606	Dqx1
Thymus	T9583	NM_011053	Pdcd11
Thymus	T6541	NM_010386	H2-DMa
Thymus	T6570	NM_008225	Hcls1
Thymus	T6555	NM_010877	Ncf2
Thymus	T533	NM_026192	Calcoco1
Thymus	T6567	NM_145559	Slc2a9
Thymus	T35102	NM_175105	Aqp11
Thymus	T35018	NM_178650	Tbc1d10c
Thymus	T35758	NM_030732	Tbl1xr1
Thymus	T6711	NM_008152	Gpr65
Thymus	T7349	NM_010693	Lck
Thymus	T9917	NM_207246	Rasgrp3
Thymus	T7093	NM_008368	Il2rb
Thymus	T9918	NM_001004184	MGC74379

Thymus	T3025	NM_011190	Psme2
Thymus	T2028	NM_183264	5830405N20Rik
Thymus	T35133	NM_007720	Ccr8
Thymus	T35190	NM_019925	Gpr132
Thymus	T35655	NM_028657	F630110N24Rik
Thymus	T36240	NM_173028	Vps13a
Thymus	T2801	NM_007651	Cd53
Thymus	T2820	NM_134116	Gpsm3
Thymus	T2888	NM_023137	Ubd
Thymus	T2937	NM_016933	Ptprcap
Thymus	T36133	NM_007648	Cd3e
Thymus	T1223	NM_028058	Fundc1
Thymus	T1211	NM_009533	Xrcc5
Thymus	T3455	NM_018729	Cd244
Thymus	T2454	NM_013542	Gzmb
Thymus	T2424	NM_172900	Siglec10
Thymus	T35818	XM_905818	A430107D22Rik
Thymus	T37003	NM_017461	Sept1
Thymus	T36899	NM_011159	Prkdc
Thymus	T36907	NM_013585	Psmb9
Thymus	T36908	NM_025959	Psmc6
Thymus	T36215	NM_009985	Ctsw
Thymus	T36527	NM_008400	Itgal
Thymus	T36949	NM_018750	Rassf5
Thymus	T31100	NM_021274	Cxcl10
Thymus	T31131	NM_133978	Cmtm7
Thymus	T31140	NM_011854	Oasl2
Thymus	T30531	NM_027222	2010001M09Rik
Thymus	T30282	NM_019583	Il17rb
Thymus	T30799	NM_145545	Gbp6
Thymus	T31172	NM_007763	Crip1
Thymus	T31200	NM_009895	Cish
Thymus	T31211	NM_030253	Parp9
Thymus	T38318	NM_025846	Rras2
Thymus	T38314	NM_025396	Pgls
Thymus	T31243	NM_007783	Csk
Thymus	T36293	XM_908064	Dnajc13
Thymus	T36570	NM_013707	Krtap14
Thymus	T36604	NM_013825	Ly75
Thymus	T9961	NM_008527	Klrb1c
Thymus	T37770	NM_183390	Klhl6
Thymus	T30348	XM_486159	2310066E14Rik
Thymus	T36501	NM_008359	Il17ra
Thymus	T36123	NM_009138	Ccl25
Thymus	T37006	NM_008458	Serpina3c
Thymus	T37053	NM_011409	Slfn3
Thymus	T31279	NM_008365	Il18r1
Thymus	T31285	NM_019640	Pitpnb
Thymus	T38070	NM_021053	Tscot
Thymus	T31336	NM_026218	Fgfr1op2
Thymus	T31435	NM_023514	Mrps9
Thymus	T31590	XM_916862	Nalp6
Thymus	T31931	NM_001038664	Gngt2
Thymus	T38672	NM_028785	Dock8
Thymus	T39052	NM_011815	Fyb
Thymus	T39623	XM_622820	LOC547338
Thymus	T8284	NM_010161	Evi2a
Thymus	T45280	NM_175479	A330008L17Rik
Thymus	T45003	AK030875	AI427122
Thymus	T31637	NM_010724	Psmb8
Thymus	T38286	NM_007645	Cd37
Thymus	T31443	NM_016857	Exoc7
Thymus	T63013	NM_018872	D1Bwg0491e
Thymus	T491	NM_178911	Pld4
Spleen	T30275	NM_013591	Madcam1
Spleen	T30080	NM_009875	Cdkn1b
URINARY SYSTEM			
Kidney	T4745	NM_177450	Cndp1
Kidney	T4117	NM_207680	Bcl2l11
Kidney	T1002	NM_026183	1300013J15Rik
Kidney	T995	NM_008269	Hoxb6
Kidney	T299	NM_146028	Stac2
Kidney	T6362	AK166607	Steap2
Kidney	T7563	NM_133894	Ugt2b38
Kidney	T8699	NM_013910	Fbxl10
Kidney	T7626	NM_009327	Tcf1
Kidney	T5441	NM_153535	BC035537
Kidney	T455	NM_015749	Tcn2
Kidney	T9926	NM_026085	3110049J23Rik
Kidney	T1358	NM_021517	Pdzk1
Kidney	T35983	NM_173427	Klhdc7a
Kidney	T7191	NM_146071	Muc20
Kidney	T36846	NM_008982	Ptprj
Kidney	T30840	NM_008116	Ggt1

Kidney	T30857	NM_007607	Car4
Kidney	T37514	NM_001004150	A4galt
Kidney	T30962	NM_027857	Acy3
Kidney	T31050	NM_016785	Tpmt
Kidney	T37659	XM_284236	D630042F21Rik
Kidney	T31548	NM_178413	BC051244
Kidney	T31646	NM_133962	Arhgef18
Kidney	T32005	NM_030021	D730039F16Rik
Kidney	T38208	NM_010018	Dao1
Kidney	T39640	XM_001003628	1110060D06Rik
Kidney	T51042	BC022226	Slc5a2
Kidney	T51027	NM_183354	Slc12a1
Bladder	T3986	NM_018802	Syt8
Bladder	T5860	NM_027320	Ifi35
REPRODUCTIVE SYSTEM			
Male	T5995	NM_009357	Tex261
Male	T6691	NM_029763	Polr3f
Male	T6628	NM_027838	Senp8
Male	T6854	NM_170591	Nupl1
Male	T7684	NM_025274	Dppa5
Male	T4919	NM_018869	Gprk5
Male	T2609	NM_011901	Taf7
Male	T484	NM_008508	Lor
Male	T8093	NM_009337	Tcl1
Male	T36303	NM_139218	Dppa3
Male	T233	NM_145546	Gtf2b
Male	T3460	NM_011328	Sct
Male	T2492	NM_030564	Rnf34
Male	T37312	NM_028034	2410004F06Rik
Male	T31260	NM_146198	Slc5a11
Male	T37647	XM_125673	Cxxc6
Male	T31584	NM_001010826	Kctd14
Male	T39844	BC059932	2410025L10Rik
Male	T63197	NM_145833	Lin28
Male	T39525	AK145040	Gtf3c3
Male	T39854	NM_001042503	Trim71
Male	T70394	MIMAT0000538	mmu-miR-31
Male	T35928	NM_007445	Amh
Female	T9706	NM_026489	Hormad1
Female	T36277	NM_010029	Ddx4
Female	T40010	XR_002340	6330411D24Rik
SENSORY ORGANS			
Ear	T4760	NM_024412	Clcnka
Ear	T36726	NM_139310	Otoa
Ear	T37121	NM_009347	Tecta
Ear	T37122	NM_009350	Tenr
Ear	T37639	XM_908254	Cldn22
Ear	T30400	NM_010953	Oc90
Ear	T38650	NM_001018019	AY616753
Ear	T40039	XM_992778	EG624918
Ear	T45144	NM_013624	Otog
Ear	T31019	NM_027172	2310046K01Rik
Ear	T37802	NM_001009574	Taar5
Ear	T37815	NM_001033292	Espnl
Eye	T1692	NM_153158	E130308A19Rik
Eye	T9121	NM_007774	Cryga
Eye	T1845	NM_009965	Cryba1
Eye	T35173	NM_016975	Gja3
Eye	T35175	NM_008123	Gja8
Eye	T30651	NM_009751	Bfsp1
Eye	T37225	NM_178679	Zfp365
Eye	T39036	NM_011939	Hsf4
Eye	T36200	NM_007773	Crybb2
Eye	T36517	XM_909063	Ipo8
Eye	T37688	XR_005138	E130119H09Rik
Eye	T36110	NM_007601	Capn3
Eye	T37644	NM_153076	Crygn
Eye	T30396	NM_019689	Arid3b
Eye	T32146	NM_172872	Ankrd38
Eye	T40285	NM_028736	Grip1
Eye	T39505	AK043703	A830021M18
Eye	T39438	XM_112126	Nhs
Eye	T45578	NM_028029	Dnmbp
Nose	T818	NM_176963	Galm
Nose	T410	NM_134017	Mat2b
Nose	T910	NM_007954	Es1
Nose	T4777	NM_009997	Cyp2a4
Nose	T4445	NM_008971	Ptk9
Nose	T4562	NM_201234	D630030L16Rik
Nose	T5330	NM_147091	Olfr568
Nose	T5328	NM_146822	Olfr640
Nose	T5333	NM_147072	Olfr641
Nose	T5353	XM_358344	Cep2
Nose	T5260	NM_146177	Suv420h2

Nose	T2137	NM_024244	1200015N20Rik
Nose	T163	NM_133699	Atp6v1c2
Nose	T5331	NM_001011536	Olfr566
Nose	T5322	NM_147088	Olfr569
Nose	T5314	NM_147085	Olfr571
Nose	T5317	NM_147115	Olfr578
Nose	T5319	NM_147052	Olfr589
Nose	T5321	NM_013621	Olfr69
Nose	T1094	NM_008867	Pla2g1br
Nose	T4312	NM_203509	NP_TR6JSE50FPA
Nose	T4182	NM_144917	Rbed1
Nose	T4357	NM_198311	Ttc8
Nose	T4186	NM_173862	BC030396
Nose	T6320	NM_030074	Zfp687
Nose	T7397	NM_183161	BC019537
Nose	T5943	NM_020276	Nelf
Nose	T6318	NM_027548	Serpinb7
Nose	T7771	NM_178414	BC048390
Nose	T6385	NM_198623	Ubqln3
Nose	T6328	NM_008299	Dnajb3
Nose	T6981	NM_172338	Dnajc16
Nose	T7794	NM_080467	Atp6v0a4
Nose	T7787	NM_011126	Plunc
Nose	T7824	NM_028934	4930452B06Rik
Nose	T7821	NM_175402	Rbm15b
Nose	T7855	NM_001008230	Gm605
Nose	T7204	NM_011183	Psen2
Nose	T6415	NM_001004066	Zfp386
Nose	T7539	NM_177406	MGC25972
Nose	T7540	NM_021372	Sertad2
Nose	T7694	NM_013809	Cyp2g1
Nose	T7704	NM_008578	Mef2b
Nose	T7712	NM_018751	Sult1c1
Nose	T8055	NM_201370	BC052883
Nose	T7591	NM_007647	Entpd5
Nose	T8722	NM_027977	2310001A20Rik
Nose	T8770	NM_182995	6330503K22Rik
Nose	T4143	NM_144536	Cdkal1
Nose	T348	NM_145355	Rnf185
Nose	T7494	NM_144820	1700009P13Rik
Nose	T8482	NM_029992	A930031F18Rik
Nose	T8814	NM_009370	Tgfbr1
Nose	T35025	XM_888129	2300003P22Rik
Nose	T35057	XM_485677	2410131K14Rik
Nose	T35016	NM_027238	1810054D07Rik
Nose	T35079	NM_028258	Dzip1l
Nose	T35768	XM_001005685	9130229H14Rik
Nose	T35785	NM_001024619	Lrrc54
Nose	T8171	NM_023247	4733401H18Rik
Nose	T8176	NM_009477	Upp1
Nose	T7916	NM_028226	3000004N20Rik
Nose	T9480	NM_028298	Zfp655
Nose	T3960	NM_145973	Ell3
Nose	T35322	NM_134216	V1rh7
Nose	T35935	NM_023617	Aox3
Nose	T3062	NM_011260	Reg3g
Nose	T35981	NM_175516	Lrrn6c
Nose	T77	NM_172891	Styk1
Nose	T387	NM_144890	BC018465
Nose	T7114	NM_016807	Sdcbp
Nose	T7206	NM_017465	Sult2b1
Nose	T35644	NM_025725	Ccdc96
Nose	T35650	XM_910825	4921528H16Rik
Nose	T36192	NM_007755	Cpeb1
Nose	T35476	NM_181853	Trim66
Nose	T35444	NM_019739	Foxo1
Nose	T3496	NM_023631	Aox4
Nose	T36638	NM_026779	Mocos
Nose	T37020	NM_145838	St8sia6
Nose	T30873	NM_007837	Ddit3
Nose	T30926	NM_145532	Mall
Nose	T31110	NM_023135	Sult1e1
Nose	T31771	NM_134109	Ildr1
Nose	T30516	NM_172205	Sbsn
Nose	T30226	NM_145554	Ldlrap1
Nose	T30794	NM_028634	Pgea1
Nose	T31226	NM_009736	Bag1
Nose	T38110	NM_031391	Gtf2a1
Nose	T31239	BC031933	Lipl3
Nose	T38356	XM_924923	Fmo6
Nose	T9396	NM_026298	4930553F24Rik
Nose	T9430	NM_008448	Kif5b
Nose	T36587	NM_144556	Lgi4
Nose	T37399	XM_133663	4931431F19Rik

Nose	T37336	XM_143418	Ankrd35
Nose	T37388	XM_127142	4930573I19Rik
Nose	T37531	NM_177389	Mia3
Nose	T37532	NM_178778	A930041I02Rik
Nose	T30379	BC049156	Ift74
Nose	T30323	NM_009379	Thpo
Nose	T31714	NM_001001182	Baz2b
Nose	T4455	NM_198023	Rcor1
Nose	T30681	NM_178775	Rps6kc1
Nose	T37038	XM_925590	Slc4a11
Nose	T35795	NM_172836	9930021J03Rik
Nose	T30419	XM_908714	Cnfn
Nose	T30412	NM_053184	Ugt2a1
Nose	T30420	NM_001039042	Klk13
Nose	T30395	XM_978169	Cep63
Nose	T31250	NM_175250	2810007J24Rik
Nose	T37668	XM_885173	D930020B18Rik
Nose	T31355	NM_053262	Dhrs8
Nose	T38250	NM_010092	Dyrk1b
Nose	T39096	NM_146867	Olfr131
Nose	T39098	NM_146852	Olfr1339
Nose	T39100	NM_146541	Olfr1361
Nose	T39103	NM_146467	Olfr1388
Nose	T39105	NM_146877	Olfr1395
Nose	T39106	NM_020515	Olfr140
Nose	T39108	NM_146881	Olfr1404
Nose	T39109	NM_146491	Olfr1410
Nose	T39110	NM_146410	Olfr1420
Nose	T39111	NM_146806	Olfr143
Nose	T39114	NM_146335	Olfr19
Nose	T39115	NM_001001807	Olfr234
Nose	T39116	NM_146606	Olfr24
Nose	T39117	NM_146457	Olfr282
Nose	T39119	NM_146281	Olfr284
Nose	T39121	NM_146538	Olfr315
Nose	T39233	NM_146374	Olfr368
Nose	T39128	NM_146722	Olfr429
Nose	T39243	NM_001011742	Olfr479
Nose	T39244	NM_146952	Olfr522
Nose	T39260	NM_147100	Olfr614
Nose	T39261	NM_147080	Olfr615
Nose	T39266	NM_013616	Olfr64
Nose	T39268	NM_147074	Olfr653
Nose	T39271	NM_013619	Olfr67
Nose	T39272	NM_146760	Olfr672
Nose	T39275	NM_207557	Olfr681
Nose	T39277	NM_001011857	Olfr685
Nose	T39280	NM_147061	Olfr691
Nose	T39281	NM_019486	Olfr71
Nose	T39283	NM_146392	Olfr720
Nose	T39288	NM_146664	Olfr734
Nose	T39290	NM_146666	Olfr736
Nose	T39291	NM_146299	Olfr745
Nose	T39298	NM_001011748	Olfr867
Nose	T38655	XM_485838	Klhdc5
Nose	T39259	NM_147081	Olfr610
Nose	T39300	NM_146816	Olfr923
Nose	T39302	NM_146514	Olfr96
Nose	T39548	XM_980715	EG231836
Nose	T39251	NM_146361	Olfr557
Nose	T39050	XM_001001987	Brd1
Nose	T39556	XM_985665	LOC671232
Nose	T45078	NM_010272	Gdf11
Nose	T30382	XM_128924	Rbm27
Nose	T39160	NM_147013	Olfr1038
Nose	T39161	NM_146577	Olfr1043
Nose	T39162	NM_147010	Olfr1052
Nose	T39164	NM_001011825	Olfr1105
Nose	T39133	NM_146821	Olfr629
Nose	T39134	NM_146814	Olfr665
Nose	T39136	NM_147032	Olfr705
Nose	T39137	NM_146682	Olfr76
Nose	T39139	NM_146330	Olfr958
Nose	T39141	NM_146854	Olfr982
Nose	T39142	NM_146855	Olfr985
Nose	T38787	NM_009701	Aqp5
Nose	T39163	NM_146767	Olfr1104
Nose	T39165	NM_146752	Olfr1106
Nose	T39168	NM_001011868	Olfr1178
Nose	T39169	NM_146917	Olfr1179
Nose	T39171	NM_146630	Olfr123
Nose	T39172	NM_146789	Olfr1230
Nose	T39173	NM_146454	Olfr1231
Nose	T39174	NM_147062	Olfr124

Nose	T39175	NM_146290	Olfr125
Nose	T39176	NM_146794	Olfr1263
Nose	T39177	NM_146793	Olfr1271
Nose	T39178	NM_146396	Olfr1277
Nose	T39181	NM_207240	Olfr1320
Nose	T39182	NM_207631	Olfr1321
Nose	T39184	NM_146390	Olfr1323
Nose	T39166	NM_146661	Olfr1112
Nose	T39365	AK047713	C030016D13Rik
Nose	T39179	NM_146400	Olfr1288
Nose	T39186	NM_146398	Olfr1325
Nose	T39187	NM_177061	Olfr1344
Nose	T39188	NM_207136	Olfr1349
Nose	T39189	NM_146389	Olfr1350
Nose	T39190	NM_001011737	Olfr1357
Nose	T39192	NM_146533	Olfr1367
Nose	T39199	NM_146683	Olfr1441
Nose	T39201	NM_146505	Olfr148
Nose	T39202	NM_001011832	Olfr1490
Nose	T39203	NM_146989	Olfr1496
Nose	T39204	NM_008762	Olfr15
Nose	T39206	NM_020514	Olfr1509
Nose	T39209	NM_010983	Olfr2
Nose	T39210	NM_146912	Olfr211
Nose	T39211	NM_146759	Olfr214
Nose	T39212	NM_001011789	Olfr222
Nose	T39213	NM_146429	Olfr223
Nose	T39649	NM_178214	Hist2h2be
Nose	T40283	AK014457	Traf3ip1
Nose	T30408	NM_027041	1700003M02Rik
Nose	T31480	NM_146107	Actr1b
Nose	T37968	XM_203999	Rapgef2
Nose	T63095	XM_358344	Cep2
Nose	T63370	NM_028932	Eaf1
Nose	T39758	AK078387	6530439I21
Nose	T45417	XM_917798	Cecr2
Nose	T7707	NM_146520	Olfr536
Nose	T7722	NM_146339	Olfr77
Nose	T38844	NM_183147	Sprn
Nose	T70019	MIMAT0001537	mmu-miR-429
Nose	T70305	MIMAT0000221	mmu-miR-191
Nose	T70269	MIMAT0000153	mmu-miR-141
Nose	T70270	MIMAT0000155	mmu-miR-142-3p
Nose	T70319	MIMAT0000519	mmu-miR-200a
Nose	T70321	MIMAT0000657	mmu-miR-200c
Nose	T70356	MIMAT0000534	mmu-miR-26b
Nose	T70358	MIMAT0000126	mmu-miR-27b
ENDOCRINE ORGANS			
Thyroid	T1897	NM_008245	Hhex
Thyroid	T4827	NM_027391	0610009A07Rik
Thyroid	T37133	NM_009375	Tg
Thyroid	T45492	NM_019804	B4galt4
Adrenal gland	T328	NM_029688	Srxn1
Adrenal gland	T847	NM_080289	Grhpr
Adrenal gland	T4605	NM_177322	Agtr1
Adrenal gland	T5203	NM_146154	Ppp1r8
Adrenal gland	T2996	NM_025384	Dnajd1
Adrenal gland	T2213	NM_027828	9030611O19Rik
Adrenal gland	T1096	NM_025826	Acadsb
Adrenal gland	T7620	NM_007809	Cyp17a1
Adrenal gland	T8009	NM_013821	Hsd3b6
Adrenal gland	T8283	NM_153779	Amid
Adrenal gland	T7281	NM_026566	9430023L20Rik
Adrenal gland	T35021	NM_025929	2010109I03Rik
Adrenal gland	T8218	NM_026343	Stx17
Adrenal gland	T6795	NM_013463	Gla
Adrenal gland	T6708	NM_172398	2310005E10Rik
Adrenal gland	T7219	NM_146099	D19Wsu162e
Adrenal gland	T35261	NM_008560	Mc2r
Adrenal gland	T36524	NM_008396	Itga2
Adrenal gland	T37215	NM_011723	Xdh
Adrenal gland	T36514	NM_010568	Insr
Adrenal gland	T36592	NM_022883	Lpin3
Adrenal gland	T9948	NM_178098	4930486L24Rik
Adrenal gland	T37710	NM_177653	F830045P16Rik
Adrenal gland	T31041	NM_133688	Lyrm5
Pituitary gland	T35545	NM_032005	Tbx19
Pituitary gland	T8212	NM_020282	Nqo2
Pituitary gland	T36053	NM_138313	Bmf
Pituitary gland	T38673	NM_027533	Tspan2
Pituitary gland	T70187	MIMAT0000677	mmu-miR-7a
