# Supplementary material for: A High-Resolution Anatomical Atlas of the Transcriptome in the Mouse Embryo
Source: PLoS Biol. 2011 Jan 18;9(1):e1000582. doi: 10.1371/journal.pbio.1000582 (PMC3022534; doi:10.1371/journal.pbio.1000582)
Supplement: Table S4 — Distribution of genes with restricted spatial expression in different anatomical structures. (0.09 MB PDF) [file pbio.1000582.s012.pdf]

Table S4. Distribution of genes with restricted spatial expression in different anatomical structures.

Structure	Structure -specific genes (n)	Genes with unknown function (n)
**Central nervous system**		
Forebrain		
Cerebral cortex	20	4
Corpus striatum	1	
Thalamus	5	-
Hypothalamus	3	1
Midbrain	*11*	3
Hindbrain		
Cerebellum	7	1
Pons	6	1
Medulla oblongata	10	3
Spinal cord	18	6
Cranial ganglia	25	6
**Peripheral nervous system**		
Spinal ganglia	14	4
Sympathetic ganglia	4	-
**Alimentary system**		
Salivary glands	39	10
Pharynx	5	2
Esophagus	3	1
Stomach	8	3
Intestine	17	3
Liver	146	15
**Respiratory system**		
Trachea	1	-
Lung	20	7
**Heart**	41	7

**Limbs**	11	1
**Skeleton**	45	6
**Skeletal muscle**	20	4
**Skin**	32	3
**Hemolymphoid system**		
Thymus	127	21
Spleen	2	-
**Urinary system**		
Kidney	29	7
Bladder	2	-
**Reproductive system**		
Male	23	2
Female	3	1
**Sensory organs**		
Ear	12	3
Eye	19	3
Nose	226	33
**Endocrine organs**		
Thyroid	4	-
Adrenal gland	24	7
Pituitary	5	-
