# Supplementary material for: A High-Resolution Anatomical Atlas of the Transcriptome in the Mouse Embryo
Source: PLoS Biol. 2011 Jan 18;9(1):e1000582. doi: 10.1371/journal.pbio.1000582 (PMC3022534; doi:10.1371/journal.pbio.1000582)
Supplement: Table S5 — Evaluation in the adult mouse brain of the expression of the genes expressed exclusively in the CNS at E14.5. (0.34 MB PDF) [file pbio.1000582.s013.pdf]

Table S5. Comparison between embryonic and adult brain expression of a set of genes exclusively expressed in the mouse CNS at E14.5.

Gene Symbol	Stage/tissue	Cerebral Cortex	Striatum	Thalamus	Hypothalamus	Midbrain	Cerebellum	Pons	Medulla	Spinal cord	
Asb3	E14.5 embryo	+									
	Adult brain	++	+	+	+	+	+	+	+	+	
9030425E11Rik	E14.5 embryo	++									
	Adult brain	++	+	+	+	+	+	+	+	+	
Cacng8	E14.5 embryo	++									
	Adult brain	++	++	+							
3110048E14Rik	E14.5 embryo	++									
	Adult brain	++	+	+	+	+	+				
Cog1	E14.5 embryo	++									
	Adult brain	++	+	+	+	+	+	+	+	+	
Gpr12	E14.5 embryo	+									
	Adult brain	+	+	++	++						
6330412F12Rik	E14.5 embryo	++									
	Adult brain	++	++	++	++	++	+	+	+	++	
Gpr21	E14.5 embryo	++									
	Adult brain	++	+	+	+	+	+	+	+	+	
Nfe2l3	E14.5 embryo	++									
	Adult brain	+	+	++	+	+	++	+	++	+	
Dmrta1	E14.5 embryo	+									
	Adult brain										
A930024E05Rik	E14.5 embryo	++									
	Adult brain	++	+	+	+	+	+	+	+	+	
Dgki	E14.5 embryo	+									
	Adult brain	++	++	++	+	+	+	+	+	+	
Sec24d	E14.5 embryo	++									
	Adult brain	+									
Wdr8	E14.5 embryo	++									
	Adult brain										
Vrk2	E14.5 embryo	++									
	Adult brain	+	+								
Abcd4	E14.5 embryo	++									
	Adult brain	++	+	+	+	+	+	+	+	+	
Lnx2	E14.5 embryo	++									
	Adult brain		+	+	+	+	+	+	+	+	
8430408J09Rik	E14.5 embryo	+									
	Adult brain										
Dyrk2	E14.5 embryo	++									
	Adult brain	++									
mmu-miR-701	E14.5 embryo	++									
	Adult brain										
Ripk5	E14.5 embryo		++								
	Adult brain	++	++	+	+	+	+	+	+	+	
Pdzrn3	E14.5 embryo	+		++							
	Adult brain	++		+							
Chst1	E14.5 embryo	++		++							
	Adult brain	++	++	++	++	++	++	++	++	++	
Epha8	E14.5 embryo	+		++		++	+	+			
	Adult brain			++		++	++	+			
Tarsl2	E14.5 embryo			++							
	Adult brain	+	++	+	+	+	+	+	++	+	
Hrmt1l6	E14.5 embryo			++							
	Adult brain			++							

Gene Symbol	Stage/tissue	Cerebral Cortex	Striatum	Thalamus	Hypothalamus	Midbrain	Cerebellum	Pons	Medulla	Spinal cord	
Gpr151	E14.5 embryo			++							
	Adult brain			++							
Rfrp	E14.5 embryo	++			++						
	Adult brain	++	+	+	+	+	++	+	+	+	
A230097C02	E14.5 embryo				++						
	Adult brain				++						
Tial1	E14.5 embryo				++						
	Adult brain	++									
2700094F01Rik	E14.5 embryo					+					
	Adult brain										
Rassf8	E14.5 embryo					++					
	Adult brain	++	++	+	+	+	++	+	+	+	
Cldn19	E14.5 embryo					++	++				
	Adult brain										
9630008K15Rik	E14.5 embryo					++	++				
	Adult brain										
Clbn4	E14.5 embryo					++	+				
	Adult brain	++			+	++	+				
Smpd4	E14.5 embryo					+					
	Adult brain	++	+	+	+	+	+	+	+	+	
1500003O22Rik	E14.5 embryo					++					
	Adult brain										
Tnrc5	E14.5 embryo					++					
	Adult brain	++	+	+	+	+	+	+	+	+	
Mipol1	E14.5 embryo					++					
	Adult brain					++					
4930589M24Rik	E14.5 embryo					++		+	+		
	Adult brain	++				++		+	+		
Mllt4	E14.5 embryo					++					
	Adult brain	+	+	+	+	+	+	+	+		
C1qtnf11	E14.5 embryo					++					
	Adult brain					++					
Oraov1	E14.5 embryo						+				
	Adult brain	+	+	+	+	+	+	+	+		
6330406I15Rik	E14.5 embryo						++				
	Adult brain	+	+	+	+	+	++	+	+	+	
Hs6st3	E14.5 embryo						++				
	Adult brain	++	+	+	+	+	+	+	+	+	
Slc1a6	E14.5 embryo						++				
	Adult brain						++				
Bcl9	E14.5 embryo						++				
	Adult brain	++									
Man1c1	E14.5 embryo						++				
	Adult brain	+	+	+	+	+	+	+	+		
Sh3md2	E14.5 embryo						++				
	Adult brain	++	++	+	+	+	+	+			
Brs3	E14.5 embryo							++			
	Adult brain							+	+		
Pnlip	E14.5 embryo							++			
	Adult brain										
Es2el	E14.5 embryo							++			
	Adult brain							++			
Tbx10	E14.5 embryo							++			
	Adult brain	+	+	+	+	+	++	++	+		
Foxd4	E14.5 embryo							++			
	Adult brain	+					++	++	+		
Gm644	E14.5 embryo							+	++		
	Adult brain	+	+	+	+	+	+	+	+	+	

Gene Symbol	Stage/tissue	Cerebral Cortex	Striatum	Thalamus	Hypothalamus	Midbrain	Cerebellum	Pons	Medulla	Spinal cord	
Nradd	E14.5 embryo								++		
	Adult brain	++									
Lhcgr	E14.5 embryo								++		
	Adult brain										
A630033E08Rik	E14.5 embryo								++		
	Adult brain										
A630052C17Rik	E14.5 embryo								++		
	Adult brain										
Nkx6.3	E14.5 embryo								++		
	Adult brain								++		
Creg2	E14.5 embryo								++		
	Adult brain	++	++	++	++	++	++	++	++	++	
Car5b	E14.5 embryo								++		
	Adult brain										
2610301K12Rik	E14.5 embryo								+	+	
	Adult brain										
Itpr2	E14.5 embryo								++		
	Adult brain								++		
Hoxa4	E14.5 embryo								++	++	
	Adult brain										
Hoxa6	E14.5 embryo								++	++	
	Adult brain										
2210407C18Rik	E14.5 embryo								++	++	
	Adult brain									+	
Pkd2l1	E14.5 embryo								+	++	
	Adult brain									++	
Ece2	E14.5 embryo					+			+	+	
	Adult brain	+	+	+	+	+	+	+	+	+	
Ghsr	E14.5 embryo					+			+	++	
	Adult brain	+	+	+	+	+	+	+	+	++	
AA407659	E14.5 embryo					+			+	+	
	Adult brain										
1700010C24Rik	E14.5 embryo								+	+	
	Adult brain									++	
Syt3	E14.5 embryo									+	
	Adult brain	+	+	+	+	+	+	+	+	+	
Hcn2	E14.5 embryo									++	
	Adult brain	+	+	+	+	+	+	+	+	+	
4931429I11Rik	E14.5 embryo									++	
	Adult brain										
Tmem77	E14.5 embryo									+	
	Adult brain										
Cabp7	E14.5 embryo									+	
	Adult brain	+	+	+	+	+	++	++	++	++	
4930459I23Rik	E14.5 embryo									++	
	Adult brain	+	+	+	+	+	+	+	+	+	
D030040B21	E14.5 embryo									++	
	Adult brain	+	+	+	+	+	+	+	+		
P42pop	E14.5 embryo					+			+	+	
	Adult brain	+	+	+	+	+	+	+	+	++	

In the brain regions analyzed signal intensity was scored (+, ++, +++).
Color code for expression analysis comparison between embryonic and adult stages: equivalent (green), partial equivalence (yellow) and different (red).
