# Supplementary material for: A High-Resolution Anatomical Atlas of the Transcriptome in the Mouse Embryo
Source: PLoS Biol. 2011 Jan 18;9(1):e1000582. doi: 10.1371/journal.pbio.1000582 (PMC3022534; doi:10.1371/journal.pbio.1000582)
Supplement: Table S6 — Comparison of Slc expression patterns between embryonic and adult mouse brain. (0.36 MB PDF) [file pbio.1000582.s014.pdf]

Table S6. Comparison of Slc gene expression patterns between embryonic and adult mouse brain

Gene Symbol	Stage/tissue	Cerebral Cortex	Striatum	Thalamus	Hypothalamus	Midbrain	Cerebellum	Pons	Medulla	Vasculature	Choroid Plexus	Meninges
Slc1a2	E14.5 embryo	++	++	+++	++	++	++	++	+++			
	Adult brain	+++	++	+++	++	++	+++	++	+++			
Slc1a3	E14.5 embryo	+++	+++	+++	+++	+++	+++	+++	+++			++
	Adult brain	+++	+++		+++		+++					
Slc1a4	E14.5 embryo									+++		+++
	Adult brain											
Slc1a5	E14.5 embryo	+++				+++					+	+++
	Adult brain	++	++	++	++	++		++	++		++	++
Slc1a6	E14.5 embryo						+++					
	Adult brain						+++					
Slc2a1	E14.5 embryo									+++		+++
	Adult brain									+++		+++
Slc2a3	E14.5 embryo	+	+	+	+	+	+	++	++			
	Adult brain	+	+	+	+	+	+	+	+			
Slc2a12	E14.5 embryo	++	++	++	++	++	+	++	++		++	
	Adult brain	++	++	+	+	+	+	+			+++	
Slc3a2	E14.5 embryo									+++	+	+++
	Adult brain											
Slc4a2	E14.5 embryo										+++	
	Adult brain					+	+	+	+		+++	+++
Slc4a3	E14.5 embryo	+		+++	+++	+++	+++	+++	+++		++	
	Adult brain	+++	+++	+++	+++	+++	+++	+++	+++		++	
Slc4a4	E14.5 embryo					+++		+++	+++			
	Adult brain		+++				+++					
Slc4a5	E14.5 embryo										++	
	Adult brain										+++	
Slc4a8	E14.5 embryo	+++		+++	+++	+++	+++	+++	+++			
	Adult brain											
Slc4a10	E14.5 embryo	+++				+	+	+	+		+++	
	Adult brain	+++	+	+	+	+	++	+	+		+++	
Slc5a3	E14.5 embryo										+++	
	Adult brain										+++	
Slc5a7	E14.5 embryo					++		++	++			
	Adult brain		+			+++		+++	+++			
Slc6a1	E14.5 embryo	+++	+++	+++	+++	+++	+++	+++	+++			+++
	Adult brain	+++	+++		+++	++	+++		++			
Slc6a2	E14.5 embryo							+++				
	Adult brain							+++				
Slc6a3	E14.5 embryo					+++						
	Adult brain					+++						
Slc6a4	E14.5 embryo					+++		+++	+++			
	Adult brain					+++		+++	+++			
Slc6a5	E14.5 embryo					+	++	+++	+++			
	Adult brain					+	+++	+++	+++			
Slc6a6	E14.5 embryo									+++		+++
	Adult brain						++					
Slc6a7	E14.5 embryo			++		++		+	+			
	Adult brain	++		++	++	++		++	++			
Slc6a9	E14.5 embryo					+++		+++	+++			+++
	Adult brain											++
Slc6a11	E14.5 embryo			+	+	+		+	+			
	Adult brain	+		++	++	++		++	++			
Slc6a12	E14.5 embryo											+++
	Adult brain											+++

Gene Symbol	Stage/tissue	Cerebral Cortex	Striatum	Thalamus	Hypothalamus	Midbrain	Cerebellum	Pons	Medulla	Vasculature	Choroid Plexus	Meninges	
Slc6a13	E14.5 embryo											+++	
	Adult brain									+		+++	
Slc6a15	E14.5 embryo	+				+		+	+		+++		
	Adult brain	+	+	+	+	+	+	+	+				
Slc6a17	E14.5 embryo	+++	+	+	++	+++	+	++	++				
	Adult brain	+++	+++	+++	+++	+++	+++	+++	+++				
Slc6a20	E14.5 embryo									+	++	+++	
	Adult brain									+	++	+++	
Slc7a1	E14.5 embryo									+++		+++	
	Adult brain												
Slc7a4	E14.5 embryo	+		+	+	+	+	+	+		+		
	Adult brain	+	+	+	+	+	+	+	+		++		
Slc7a10	E14.5 embryo										+++		
	Adult brain										++		
Slc7a11	E14.5 embryo										+++	+++	
	Adult brain									+++	+++	+++	
Slc8a1	E14.5 embryo	++		+	+	++	+	++	++				
	Adult brain	++		+	+	+	+	+	+				
Slc8a2	E14.5 embryo	++											
	Adult brain	+++	+++				++						
Slc8a3	E14.5 embryo	+		+	+	+	+	+	+				
	Adult brain	+	+	+	+	+	+	+	+				
Slc9a6	E14.5 embryo	+		+	+	+	+	+	+				
	Adult brain	++	++	++	++	++	++	++	++				
Slc10a3	E14.5 embryo										++		
	Adult brain										++	+++	
Slc10a4	E14.5 embryo		++			++		++	++				
	Adult brain		+++			+++		+++	+++				
Slc12a2	E14.5 embryo										++		
	Adult brain										++		
Slc13a2	E14.5 embryo	+		+	+	++	+	++	++				
	Adult brain												
Slc13a4	E14.5 embryo										++	+++	
	Adult brain										+++	+++	
Slc14a1	E14.5 embryo	+		+		+++		+++	+++				
	Adult brain												
Slc14a2	E14.5 embryo	+		++		++							
	Adult brain												
Slc15a2	E14.5 embryo	++		++		++		++	++				
	Adult brain												
Slc16a1	E14.5 embryo	++		+		++	+						
	Adult brain												
Slc16a2	E14.5 embryo	++									+++		
	Adult brain	+++									+++		
Slc16a4	E14.5 embryo										++		
	Adult brain										+		
Slc16a6	E14.5 embryo										++	++	
	Adult brain										++	+	
Slc16a9	E14.5 embryo										++	+++	
	Adult brain										+++	+++	
Slc16a10	E14.5 embryo										+++		
	Adult brain										+	+++	
Slc16a12	E14.5 embryo										++		
	Adult brain										+		
Slc16a14	E14.5 embryo	+		+	+	++	+	+	+				
	Adult brain												
Slc17a6	E14.5 embryo	+++		+++	+++	+++		+++	+++				
	Adult brain	+++		+++	+++	+++		+++	+++				

Gene Symbol	Stage/tissue	Cerebral Cortex	Striatum	Thalamus	Hypothalamus	Midbrain	Cerebellum	Pons	Medulla	Vasculature	Choroid Plexus	Meninges	
Slc17a8	E14.5 embryo		+++	++		+++		+++	+++				
	Adult brain	++	+++	++		+++		+++	++				
Slc18a2	E14.5 embryo		+++	+++	+++	+++		+++	+++				
	Adult brain				+++	+++		+++					
Slc18a3	E14.5 embryo					+++		+++	+++				
	Adult brain		+++			+++		+++	+++				
Slc20a1	E14.5 embryo	+++		+	+	+	+	+	+				
	Adult brain	++		+	+	+	+++	+	+				
Slco1a4	E14.5 embryo										++		
	Adult brain									+++	++		
Slco1a5	E14.5 embryo										++		
	Adult brain										+		
Slco1c1	E14.5 embryo									+++	+++	++	
	Adult brain									++	+++		
Slco2b1	E14.5 embryo									++		+	
	Adult brain									+			
Slco3a1	E14.5 embryo	+		+	+	+	+	+	+				
	Adult brain	+		+	+	++	+	++	++				
Slco5a1	E14.5 embryo	+	+	+	+	+	+	+	+		+		
	Adult brain	+	+	+	+	+	+	+	+		+		
Slco1a6	E14.5 embryo										+++		
	Adult brain												
Slc22a2	E14.5 embryo											+++	
	Adult brain											+	
Slc22a3	E14.5 embryo							+++	+++				
	Adult brain							+++	+++				
Slc22a4	E14.5 embryo	++	+	+	+	++	+	++	++				
	Adult brain	+		+		+	+						
Slc22a13	E14.5 embryo	++	+	+	+	+	+	+	++				
	Adult brain												
Slc23a2	E14.5 embryo										+++		
	Adult brain										+++		
Slc24a3	E14.5 embryo			++	++	++		++	++				
	Adult brain	++		++	++	++		++	++				
Slc25a1	E14.5 embryo							++	++				
	Adult brain		+++					+++	+++				
Slc25a13	E14.5 embryo	+				+					+		
	Adult brain										++		
Slc25a22	E14.5 embryo	+		+	+	+	+	+	+				
	Adult brain	+		+	+	+	+	+	+				
Slc25a27	E14.5 embryo	+	+	+	+	+	+	+	+				
	Adult brain	++	++	++	++	++	++	++	++				
Slc26a7	E14.5 embryo			+		++		+					
	Adult brain											+	
Slc27a1	E14.5 embryo	++		++	+++	+++	++	+++	+++		+		
	Adult brain	+++	+++	+++	+++	+++	+++	+++	+++		+++	+++	
Slc30a1	E14.5 embryo									++		+	
	Adult brain												
Slc30a3	E14.5 embryo			+++	+++	+++	+++	+++	+++				
	Adult brain	+++		++		++	++	++	++				
Slc31a1	E14.5 embryo	+								++	+++		
	Adult brain										++		
Slc32a1	E14.5 embryo	++	+++	+++	+++	+++	+++	+++	+++				
	Adult brain	+++	+++	+++	+++	+++	+++	+++	+++				
Slc35f1	E14.5 embryo	++		++	+	++	+++	++	++				
	Adult brain	+	+	++	+	+	+++	+	+				
Slc35f2	E14.5 embryo	++						++	++	+		+	
	Adult brain							++	++				

**Gene Symbol**	**Stage/tissue**	**Cerebral Cortex**	**Striatum**	**Thalamus**	**Hypothalamus**	**Midbrain**	**Cerebellum**	**Pons**	**Medulla**	**Vasculature**	**Choroid Plexus**	**Meninges**	
Slc36a1	E14.5 embryo					+	++	++	++				
	Adult brain	+++	+++	+++		+++	+++	+++	+++				
Slc38a1	E14.5 embryo	++		++	++	++	++	++	++				
	Adult brain	++		++	++	++	+	++	++				
Slc38a2	E14.5 embryo									+++		+++	
	Adult brain											+++	
Slc38a3	E14.5 embryo									+++	++	++	
	Adult brain									++	+++	+++	
Slc38a5	E14.5 embryo									+++		+++	
	Adult brain									++			
Slc39a3	E14.5 embryo	++	++	++	++	++	++	++	++				
	Adult brain	+	+	+	+	+	+	+	+				
Slc39a8	E14.5 embryo									+++	+	+++	
	Adult brain												
Slc39a10	E14.5 embryo									++		++	
	Adult brain	++											
Slc39a12	E14.5 embryo										+++		
	Adult brain										++		
Slc40a1	E14.5 embryo									+++	++	+++	
	Adult brain										+		
Slc41a1	E14.5 embryo											++	
	Adult brain										++	+++	
Slc43a2	E14.5 embryo	+		+	+	+	+	+	+	++		+	
	Adult brain	+	+	+	+	+	+	+	+				
Slc44a5	E14.5 embryo	+		++		++	++	+++	+				
	Adult brain												
Slc45a3	E14.5 embryo	+	+	+++		+++							
	Adult brain					+		+	+				
Slc47a1	E14.5 embryo											++	
	Adult brain											+++	

In the brain regions analyzed signal intensity was scored (+, ++, +++).
Color code for Slc gene expression analysis comparison: equivalent (green), partial equivalence (yellow) and different (red).
