# Supplementary material for: A High-Resolution Anatomical Atlas of the Transcriptome in the Mouse Embryo
Source: PLoS Biol. 2011 Jan 18;9(1):e1000582. doi: 10.1371/journal.pbio.1000582 (PMC3022534; doi:10.1371/journal.pbio.1000582)
Supplement: Table S7 — List of murine homologs of human disease genes whose tissue distribution at E14.5 is consistent with the corresponding human disease phenotype. (0.08 MB PDF) [file pbio.1000582.s015.pdf]

Table S7. List of murine homologs of human disease genes whose tissue distribution at E14.5 is consistent with the corresponding disease phenotype.

Human disease	OMIM #	Endocrine	Genitourinary	Cardiovascular	CNS	Eye	Ear	Nose	Skeletal muscle	Skeleton	Gastrointestinal	Liver	Respiratory	Skin	Teeth	Limbs	Immune system	gene symbol (mouse ortholog)
AHC	300200																	*Nr0b1*
ALGS1	118450																	*Jag1*
HPE9	610829																	*Gli2*
KNOBLOCH SYNDROME, TYPE I	267750																	*Col18a1*
MEB	253280																	*Pomgnt1*
BFPP	606854																	*Gpr56*
CHARGE	214800																	*Chd7*
OSX	300000																	*Mid1*
HPE4	142946																	*Tgif*
NALD	202370																	*Pex5*
MENTAL RETARDATION, X-LINKED	300419																	*Arx*
DFNB22	607039																	*Otoa*
DFNA12	601543																	*Tecta*
CCZS	600881																	*Cryba1*
RP, LCA	268000																	*Crx*
MULTIPLE PTERYGIUM SYNDROME	265000																	*Chrng*
MCOP3	611038																	*Rax*
MCOPS9	601186																	*Stra6*
MAC and related disorders	610125																	*Otx2*
MCOPS6	607932																	*Bmp4*
FRASER SYNDROME	219000																	*Fras1*
FRASER SYNDROME	219000																	*Frem2*
RENAL ADYSPLASIA	191830																	*Upk3a*
PPS	119500																	*Irf6*
ACG2	200610																	*Col2a1*
ACG1B	600972																	*Slc26a2*
WS1	193500																	*Pax3*
STL2	604841																	*Col11a1*
ECTODERMAL DYSPLASIA, ANHIDROTIC	612132																	*Nfkbia*
SGBS1	312870																	*Gpc3*
ATS	301050																	*Col4a5*
SPD1	186000																	*Hoxd13*
HAND-FOOT-UTERUS SYNDROME	140000																	*Hoxa13*
CMH7	192600																	*Tnni3*
CMH10	608758																	*Myl2*
CMH12	612124																	*Csrp3*
ASD4	611363																	*Tbx20*
ASD2	607941																	*Gata4*
EDMD2	181350																	*Lmna*
CMH2	115195																	*Tnnt2*
CMH9	192600																	*Myl3*
CMH8	608751																	*Ttn*
ASD3	108800																	*Myh6*
AMDH, BDC	201250																	*Gdf5*
TBS	107480																	*Sall1*
AOI	108720																	*Flnb*
FACIOGENITAL DYSPLASIA	305400																	*FGD1*
CHONDRODYSPLASIA, BLOMSTRAND TYPE; BOCD	215045																	*Pthr1*
DA2B	601680																	*Tnnt3*
DA2B	601680																	*Tnni2*
CMPD	114290																	*Sox9*
AMDM	602875																	*Npr2*
UMS	181450																	*Tbx3*
PTHSL1	610042																	*Cntnap2*
HPE3	142945																	*Shh*
HPE2	157170																	*Six3*
Aniridia and related disorders	106210																	*Pax6*
MCOPCB3	610092																	*Chx10*
CCA2	601547																	*Crybb2*
CATARACT, CONGENITAL, CERULEAN TYPE, 3; CCA3	608983																	*Cryga*
RIEG1	180500																	*Pitx2*
AI4, TDO	104510																	*Dlx3*
EXT1	133700																	*EXT1*
BDA1, ACFD	112500																	*Ihh*
BCNS	109400																	*Ptch1*
FEINGOLD SYNDROME	164280																	*Mycn*
ACLS	200990																	*vera*
CFNS	304110																	*Efnb1*
ACTH deficiency	201400																	*Tbx19*
FADS	208150																	*Rapsn*
DPD1	131300																	*Tgfb1*
SPONDYLOEPIMETAPHYSEAL DYSPLASIA	602111																	*Mmp13*
EDM2	600204																	*Col9a2*
SPONDYLOEPIPHYSEAL DYSPLASIA, KIMBERLEY TYPE	608361																	*Agc1*
SPONDYLOEPIMETAPHYSEAL DYSPLASIA, PAKISTANI TYPE	612847																	*Papss2*
MCDS	156500																	*Col10a1*
BRACHYOLMIA TYPE 3	113500																	*Trpv4*
EDM1	132400																	*Col9a1*
EDM5	607078																	*Matn3*
EDM3	600969																	*Col9a3*
SEMDC	184250																	*Col2a1*
SYNS1	186500																	*Nog*
CACP	208250																	*Prg4*
ARCL1	219100																	*Fbln5*
BSS	605041																	*Cyld*
HED	129490																	*Edaradd*
EPIDERMOLYSIS BULLOSA JUNCTIONALIS WITH PYLORIC ATRESIA	226730																	*Itgb4*
EPIDERMOLYSIS BULLOSA JUNCTIONALIS WITH PYLORIC ATRESIA	226730																	*Itga6*
HED	129490																	*Edar*
ALPS2B	607271																	*Casp8*
BLS, TYPE I	604571																	*Tap2*
SCID, AUTOSOMAL RECESSIVE	608971																	*Cd3d*
SCID, AUTOSOMAL RECESSIVE	608971																	*Cd3e*
CGD, AUTOSOMAL RECESSIVE CYTOCHROME b-POSITIVE, TYPE II	233710																	*Ncf2*
THYROID HORMONOGENESIS, GENETIC DEFECT IN, 4	274800																	*Ivd (0610009A07Rik)*
CHNG2	218700																	*Pax8*
ADT1P	175780																	*Col4a1*
DFNX2	304400																	*Pou3f4*
MCOPS5	610125																	*Otx2*
PAPILLORENAL SYNDROME	120330																	*Pax2*

Rows: selected human genetic diseases and corresponding responsible genes (mouse orthologs). Columns: tissues affected in the listed diseases, as assessed from OMIM analysis. Green cell, the gene is annotated to be expressed in the affected tissue; red cell, the gene is not annotated to be expressed in the affected tissue.
