# Supplementary material for: A High-Resolution Anatomical Atlas of the Transcriptome in the Mouse Embryo
Source: PLoS Biol. 2011 Jan 18;9(1):e1000582. doi: 10.1371/journal.pbio.1000582 (PMC3022534; doi:10.1371/journal.pbio.1000582)
Supplement: Table S8 — Expression of Wnt signaling components in the E14.5 embryo. (0.09 MB PDF) [file pbio.1000582.s016.pdf]

Table S8: Expression of Wnt signaling components in the E14.5 mouse embryo

Group	Gene Symbol	EURExpress Link	Neocortex	Hippocampus	Corpus Striatum	Septum	Thalamus	Hypothalamus	Pretectum	Superior Colliculu	Inferior Colliculus	Tegmentum	Cerebellum	SpinalCord	Skin	Lung	Kidney	Liver	Thyroid	Pituitary	Pancreas	Adrenal Gland	Heart	Thymus	Muscle System	Axial Skeleton
Wnt ligands	Wnt1	euxassay_018054																								
	Wnt2	euxassay_007181																								
	Wnt2b	euxassay_018055																								
	Wnt3	euxassay_018056																								
	Wnt3a	euxassay_018057																								
	Wnt4	euxassay_018058																								
	Wnt5a	euxassay_018099																								
	Wnt5b	euxassay_018311																								
	Wnt6	euxassay_018092																								
	Wnt7a	euxassay_018093																								
	Wnt7b	euxassay_018094																								
	Wnt8a	euxassay_018179																								
	Wnt8b	euxassay_018100																								
	Wnt9a	euxassay_018215																								
	Wnt9b	euxassay_018312																								
	Wnt10a	euxassay_018275																								
	Wnt10b	euxassay_018214																								
	Wnt11	euxassay_018309																								
	Wnt16	euxassay_018310																								
	Rspo1	euxassay_019762																								
	Rspo2	euxassay_018864																								
	Rspo3	euxassay_019787																								
	Rspo4	euxassay_018898																								
	Porcn	euxassay_002692																								
Wnt receptors	Fzd1	euxassay_007182																								
	Fzd2	euxassay_018254																								
	Fzd3	euxassay_019789																								
	Fzd4	euxassay_018160																								
	Fzd5	euxassay_018230																								
	Fzd6	euxassay_018161																								
	Fzd7	euxassay_018109																								
	Fzd8	euxassay_018287																								
	Fzd9	euxassay_008309																								
	Fzd10	euxassay_018076																								
	Kremen1	euxassay_019790																								
	Kremen2	euxassay_011015																								
	Lrp5	euxassay_019783																								
	Lrp6	euxassay_018221																								
Wnt extracellular inhibitors	Dkk1	euxassay_018073																								
	Dkk2	euxassay_018074																								
	Dkk3	euxassay_018106																								
	Dkk4	euxassay_018154																								
	Sfrp1	euxassay_018022																								
	Sfrp2	euxassay_019761																								
	Sfrp3/Frzb	euxassay_018108																								
	Sfrp4	euxassay_018118																								
	Sfrp5	euxassay_019791																								
	Wif1	euxassay_018178																								
Wnt canonical signaling	Apc	euxassay_019765																								
	Apc2	euxassay_019768																								
	Axin	euxassay_007183																								
	Axin2	euxassay_005683																								
	Bcl9	euxassay_013718																								
	Btrc	euxassay_004824																								
	Catnb	euxassay_018151																								
	Csnk1a1	euxassay_019763																								
	Dvl1	euxassay_019764																								
	Dvl2	euxassay_000142																								
	Dvl3	euxassay_019766																								
	Frat1	euxassay_019776																								
	Frat2	euxassay_018490																								
	Frat3/Peg12	euxassay_000559																								
	Grk4	euxassay_012852																								
	Gsk3b	euxassay_004227																								
	Lef1	euxassay_018171																								
	Nkd1	euxassay_005331																								
	Nkd2	euxassay_019779																								
	Pygo1	euxassay_007893																								
	Pygo2	euxassay_011247																								

Group	Gene Symbol	EURExpress Link	Neocortex	Hippocampus	Corpus Striatum	Septum	Thalamus	Hypothalamus	Pretectum	Superior Colliculus	Inferior Colliculus	Tegmentum	Cerebellum	SpinalCord	Skin	Lung	Kidney	Liver	Thyroid	Pituitary	Pancreas	Adrenal Gland	Heart	Thymus	Muscle System	Axial Skeleton
	Tcf3	euxassay_018176	weak/medium	weak/medium	weak/medium	weak/medium	weak/medium	weak/medium	weak/medium	weak/medium	weak/medium	weak/medium	weak/medium	weak/medium	strong	weak/medium	weak/medium	weak/medium		weak/medium		weak/medium	weak/medium		weak/medium	weak/medium
	Tcf4	euxassay_018303	weak/medium	weak/medium	weak/medium	weak/medium	weak/medium	weak/medium	weak/medium	weak/medium	weak/medium	weak/medium	weak/medium	strong	strong	weak/medium	weak/medium		weak/medium	weak/medium	weak/medium	weak/medium	weak/medium	weak/medium	weak/medium	weak/medium
	Tcf7	euxassay_018177	weak/medium	weak/medium	weak/medium		weak/medium	weak/medium						weak/medium	strong	strong	strong	weak/medium		weak/medium		weak/medium	weak/medium	strong	weak/medium	strong
**Wnt Ca2+ signaling**	Nfatc1	euxassay_002607															weak/medium									
	Nfatc2	euxassay_013856	weak/medium	weak/medium			weak/medium			weak/medium	weak/medium	weak/medium	weak/medium	weak/medium		weak/medium								weak/medium		
	Nfatc3	euxassay_008958																								
	Nfatc4	euxassay_003488	strong	strong		weak/medium						weak/medium		weak/medium	strong	weak/medium	weak/medium			weak/medium	weak/medium	weak/medium	weak/medium			weak/medium
	Nfat5	euxassay_019784								strong	weak/medium			weak/medium			strong		weak/medium	strong		strong		weak/medium		
	Plcd1	euxassay_018598													weak/medium		weak/medium						weak/medium	weak/medium		weak/medium
	Plcg1	euxassay_018782	weak/medium	weak/medium	weak/medium	weak/medium	weak/medium	weak/medium	weak/medium	weak/medium	weak/medium	weak/medium	weak/medium	weak/medium	weak/medium	weak/medium	weak/medium		weak/medium				weak/medium	weak/medium		
	Ppp3ca	euxassay_002802	strong	strong	weak/medium	weak/medium	weak/medium	weak/medium	weak/medium	strong	strong	strong	weak/medium	strong	strong	weak/medium	weak/medium	weak/medium	weak/medium	weak/medium	weak/medium	weak/medium	weak/medium	weak/medium		weak/medium
	Ppp3r2	euxassay_019785														strong										
**Wnt PCP signaling**	Cdc42	euxassay_000136	weak/medium	weak/medium	weak/medium	weak/medium	strong	strong	weak/medium	weak/medium	weak/medium	weak/medium	weak/medium	strong	strong	weak/medium	weak/medium	weak/medium	weak/medium		weak/medium	weak/medium	weak/medium	weak/medium	weak/medium	
	D10Ertd610e	euxassay_019769	strong	strong			weak/medium			weak/medium	weak/medium	weak/medium	weak/medium	weak/medium		weak/medium	weak/medium			weak/medium	weak/medium	weak/medium	weak/medium	weak/medium		
	Daam1	euxassay_019770	weak/medium	weak/medium		weak/medium	weak/medium	weak/medium	weak/medium	weak/medium		weak/medium	weak/medium	weak/medium		weak/medium										
	Daam2	euxassay_019771	weak/medium	weak/medium	weak/medium					weak/medium	weak/medium	strong	weak/medium	strong		strong		weak/medium								weak/medium
	Mapk8	euxassay_018521	weak/medium	weak/medium		weak/medium	weak/medium		weak/medium	weak/medium		weak/medium	weak/medium	weak/medium	weak/medium											
	Mapk9	euxassay_019788				weak/medium	weak/medium	weak/medium	weak/medium	weak/medium		weak/medium	weak/medium	strong												
	Mapk10	euxassay_019772	weak/medium	weak/medium	weak/medium	weak/medium	weak/medium		weak/medium	weak/medium	weak/medium	weak/medium		weak/medium												
	RhoA	euxassay_019777	weak/medium	weak/medium	weak/medium	weak/medium				weak/medium	weak/medium	weak/medium	weak/medium		strong	weak/medium	weak/medium									weak/medium
	Rock2	euxassay_014233																								
	Ror2	euxassay_018175				weak/medium	strong	weak/medium					strong	weak/medium	weak/medium	weak/medium								weak/medium		weak/medium
**Wnt GO:0016055**	Aes	euxassay_019767	weak/medium	weak/medium	weak/medium	weak/medium	weak/medium	weak/medium	weak/medium	weak/medium	strong	weak/medium	weak/medium	weak/medium	weak/medium	strong	strong		weak/medium	weak/medium	strong			weak/medium		
	Camta1	euxassay_011844																								
	Camta2	euxassay_008596	weak/medium	weak/medium	weak/medium	weak/medium	weak/medium	weak/medium	weak/medium	weak/medium	weak/medium	weak/medium	weak/medium	weak/medium												
	Csnk1d	euxassay_019773	weak/medium	weak/medium	weak/medium	weak/medium	weak/medium	weak/medium	weak/medium	weak/medium	weak/medium	weak/medium	weak/medium	weak/medium	weak/medium	weak/medium	weak/medium		weak/medium	weak/medium			weak/medium	weak/medium		
	Csnk1e	euxassay_006631	weak/medium	weak/medium	weak/medium	weak/medium	weak/medium	weak/medium	weak/medium	strong	strong	weak/medium	weak/medium	strong			weak/medium									
	Csnk1g1	euxassay_019774													weak/medium											
	Csnk1g2	euxassay_019775													weak/medium		weak/medium									
	Csnk2a1	euxassay_018819	weak/medium	weak/medium	weak/medium	weak/medium	weak/medium	weak/medium	weak/medium	weak/medium	weak/medium	weak/medium	weak/medium	weak/medium		weak/medium	weak/medium	weak/medium	weak/medium	weak/medium				weak/medium	weak/medium	
	Csnk2a2	euxassay_018707	weak/medium	weak/medium	weak/medium	weak/medium	weak/medium	weak/medium	weak/medium	weak/medium	weak/medium	weak/medium	weak/medium	weak/medium	weak/medium	weak/medium	weak/medium	weak/medium	weak/medium		weak/medium	weak/medium		weak/medium	weak/medium	
	Csnk2b	euxassay_003470	weak/medium	weak/medium	weak/medium	weak/medium				weak/medium	strong	weak/medium		weak/medium	strong	strong	weak/medium	strong	weak/medium	weak/medium	weak/medium		weak/medium	weak/medium		
	Ctnnbip1	euxassay_007108	strong	strong	weak/medium	weak/medium	weak/medium	weak/medium	weak/medium	weak/medium	weak/medium	weak/medium	weak/medium	weak/medium	strong	weak/medium	weak/medium		weak/medium	strong			weak/medium	weak/medium	weak/medium	weak/medium
	Ctnnd1	euxassay_019781													weak/medium	strong	strong						strong		weak/medium	
	CxxC4	euxassay_008607	weak/medium	weak/medium	strong	strong	strong	strong	strong	strong	strong	strong	weak/medium	strong			weak/medium		weak/medium	weak/medium	strong	weak/medium		weak/medium	strong	
	Dact1	euxassay_009577	strong	strong	strong	weak/medium	weak/medium	weak/medium	weak/medium	weak/medium		weak/medium	weak/medium	weak/medium			weak/medium									
	Fbxw4	euxassay_003165																								
	Hbp1	euxassay_018713	strong	strong	weak/medium					weak/medium	weak/medium	weak/medium	weak/medium	weak/medium	strong	weak/medium	weak/medium	weak/medium	weak/medium	strong	weak/medium	weak/medium	weak/medium	weak/medium	weak/medium	
	Invs	euxassay_019786	strong	weak/medium	weak/medium	weak/medium	weak/medium	weak/medium	weak/medium	strong	weak/medium	weak/medium	weak/medium	weak/medium		weak/medium	weak/medium		weak/medium	weak/medium	weak/medium	weak/medium	weak/medium	weak/medium	weak/medium	
	Ldb1	euxassay_006506	strong	strong	strong				strong	strong	strong	strong	weak/medium	weak/medium	weak/medium	weak/medium						weak/medium			weak/medium	strong
	Mitf	euxassay_019482	strong	weak/medium	strong		strong	strong	strong	strong	strong	strong	strong	strong												
	Pitx2	euxassay_019470					weak/medium	strong		strong	weak/medium			weak/medium					strong							strong
	Slc9a3r1	euxassay_019782		weak/medium	weak/medium							strong		strong	weak/medium	weak/medium	strong		weak/medium				weak/medium			
	Sostdc1	euxassay_019780															strong									strong
	Tle4	euxassay_018870	strong		strong	strong	strong	strong	weak/medium	strong	strong	strong	weak/medium	strong			strong	weak/medium	weak/medium				strong			
	Tle6	euxassay_018871																			strong					
	Wisp1	euxassay_006558														weak/medium	weak/medium					weak/medium				weak/medium
	Zbtb33	euxassay_019778																								

Legend: not detected (white) · weak · medium · strong

Note: The Wnt-signaling components are grouped into blocks for ligands, receptors, extracellular inhibitors, canonical-, Ca2+-, and PCP signaling, and additional genes annotated with the GO term “GO:0016055; Wnt receptor signaling pathway”. The table provides direct access to the ISH data of the full embryo via the linked EURexpress Assay ID.
