# Supplementary material for: A High-Resolution Anatomical Atlas of the Transcriptome in the Mouse Embryo
Source: PLoS Biol. 2011 Jan 18;9(1):e1000582. doi: 10.1371/journal.pbio.1000582 (PMC3022534; doi:10.1371/journal.pbio.1000582)
Supplement: Table S9 — Classification of single cell expression patterns in the E14.5 liver. (3.64 MB PDF) [file pbio.1000582.s017.pdf]

Table S9. Classification of single cell expression patterns in the E14.5 liver

Pattern	Gene	Function	Localization	Accession #
Group1: single scattered cells, d >> cd				
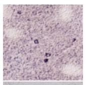	Agpat3	lipid metabolism	integral membrane protein	NM_053014
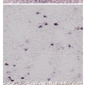	Alas1	heme biosynthesis	mitochondrial protein	NM_020559
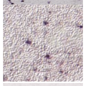	Aldoa	glycolysis	mitochondrial protein	NM_007438
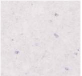	Angpt1	cytokine	secreted	NM_009640
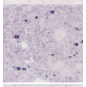	Arhgap24	GTPase activator	cytoplasmic	NM_029270
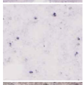	Cd200r1	receptor	integral membrane protein	NM_021325
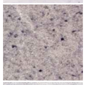	Cd48	T cell activation	plasma membrane	NM_007649
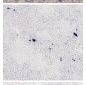	Ctgf	cytokine	secreted	NM_010217
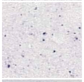	Daf2	immune response	integral membrane protein	NM_007827
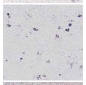	Dapp1	phosphoinositide 3-kinase regulator activity	membrane and cytoplasm	NM_011932
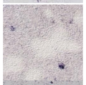	Dusp10	MAP kinase tyrosine/serine/threonine phosphatase activity	cytoplasm	NM_022019
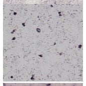	F5	blood coagulation	secreted	NM_007976
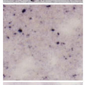	**Gata2**	transcription factor	nuclear	NM_008090
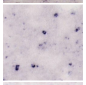	Gucy1a3	guanylate cyclase activity	cytoplasm	NM_021896
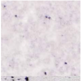	Ifitm7	unknown function	integral membrane protein	NM_028968
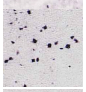	**Itga2b** (CD41)	cell adhesion	integral membrane protein	NM_010575
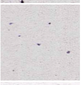	Jag1	Notch binding	integral membrane protein	NM_013822
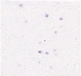	Kcnj5	ion channel	integral membrane protein	NM_010605
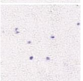	Kcnk6	ion channel	integral membrane protein	NM_001033525

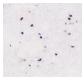	Kctd11	ion channel	integral membrane protein	NM_153143
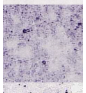	Laf4l	transcription regulation	nuclear	NM_033565
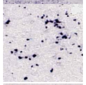	Lcn2	associated with neutrophil gelatinase	extracellular	NM_008491
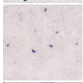	Lef1	transcription factor	nuclear	NM_010703
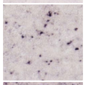	Lta4h	arachidonic acid metabolism	cytoplasm	NM_008517
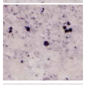	Ltbp1	Tgfb1 binding	extracellular	NM_206958
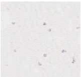	Mmrn1	candidate receptor for factor 5	secreted	XM_284198
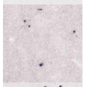	Myom1	cytoskeleton component	cytoplasm	NM_010867
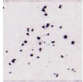	Ngp	immune response	secreted	NM_008694
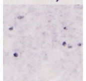	Phlda1	T-cell death associated	unknown	NM_009344
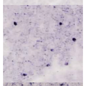	Pttg1	peptidase inhibitor activity	cytoplasm and nucleus	NM_013917
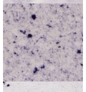	**Runx1**	transcription factor	nuclear	NM_009821
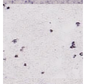	Serpine2	peptidase inhibitor activity	secreted	NM_009255
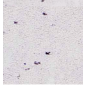	Slamf1	receptor	integral membrane protein	NM_013730
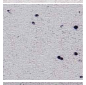	Slpi	peptidase inhibitor activity	secreted	NM_011414
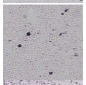	Tgfb1i1	Wnt receptor signaling pathway	focal adhesion sites	NM_009365
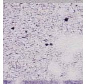	Tgfb1i4	tumor suppressor	nuclear	NM_009366
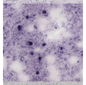	Za20d2	protein degradation	cytoplasm	NM_009551
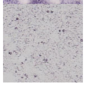	Zfhx1b	transcription factor	nuclear	AK032970

Group2: single scattered cells, d >> cd and circles of cells

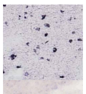	2810457I06Rik	unknown function	cytoplasm and nucleus	NM_176860
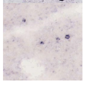	3110003A17Rik	unknown function	unknown	XM_125510

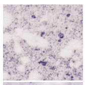	Actc1	cytoskeleton	cytoplasm	NM_009608
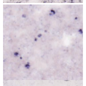	Adcy9	adenylate cyclase activity	integral membrane protein	NM_009624
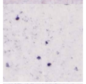	AI838661	GTP binding	cellular	NM_133884
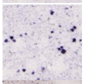	Clec1b	transmembrane receptor activity	integral membrane protein	NM_019985
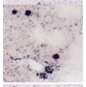	Clu	cell death: secreted	unknown	NM_013492
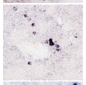	Csrp1	unknown function	unknown	BC006912
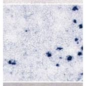	F8a	unknown function	unknown	NM_007978
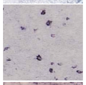	Ifi47	immune response	unknown	NM_008330
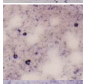	Kif5b	motor activity	cytoplasm	U86090
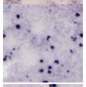	Lims1	cell-cell adhesion	focal adhesion sites	NM_026148
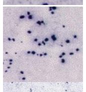	Ltf	iron ion binding	cytoplasm and extracellular	NM_008522
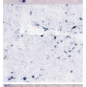	**Ly6a**		glycosylphosphotidylinositol-anchored protein	NM_010738
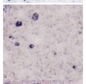	Mapk6	MAP kinase activity	cytoplasm	NM_015806
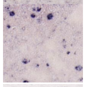	Mef2c	transcription factor	nuclear	NM_025282
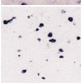	P2rx1	ion channel	integral membrane protein	NM_008771
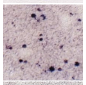	Parvb	cell adhesion	plasma membrane	AF237770
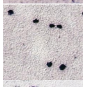	Pde5a	cyclic-nucleotide phosphodiesterase activity	cytoplasm	NM_153422
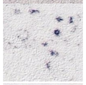	Plcg2	T cell receptor signaling pathway	unknown	NM_172285
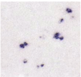	Prkca	protein kinase activity	cytoplasm	NM_011101
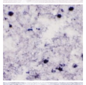	Pttg1ip	Pttg1 binding	integral membrane protein	BC025533
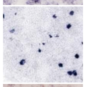	Serpinb10	peptidase inhibitor activity	cytoplasm	NM_198028
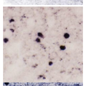	Sla	T cell receptor regulation	cytoplasm	AJ131777
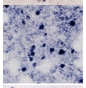	Slc14a1	solute transport	integral membrane protein	NM_028122
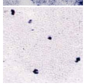	Slc6a4	solute transport	integral membrane protein	NM_010484

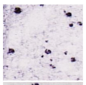	Tgfb1	cytokine	secreted	NM_011577
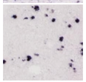	Timp3	peptidase inhibitor activity	secreted	NM_011595
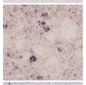	Tm7sf3	unknown function	integral membrane protein	XM_132970
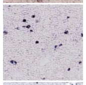	Tmem40	unknown function	integral membrane protein	NM_144805
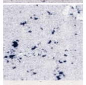	Tnnc1	cytoskeleton	cytoplasm	NM_009393
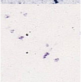	Trpc6	channel	integral membrane protein	NM_013838
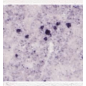	Unc119	unknown function	unknown	NM_011676
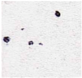	Vwf	blood coagulation	secreted	NM_011708

Group3: single scattered cells, d ≥ cd

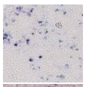	Alox5ap	arachidonic acid metabolism	integral membrane protein	NM_009663
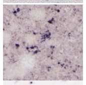	Car1	one-carbon metabolism	cytoplasm	NM_009799
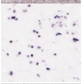	Cd34	cell adhesion	integral membrane protein	NM_133654
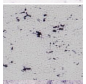	Cd5l	apoptosis	unknown	NM_009690
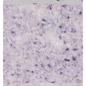	Cd68	transmembrane glycoprotein	unknown	NM_009853
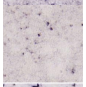	Cited2	transcription coactivator activity	nuclear	NM_010828
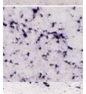	Clca2	channel	integral membrane protein	NM_030601
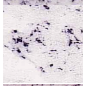	Csf1r	cytokine receptor	integral membrane protein	NM_007779
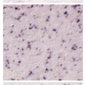	Dcn	extracellular matrix binding	secreted	NM_007833
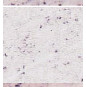	Egr1	transcription factor	nuclear	NM_007913
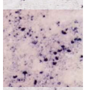	H2afy	chromatin binding	nuclear	XM_127380
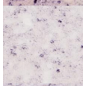	Itgb2	cell adhesion	integral membrane protein	NM_008404
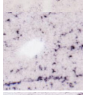	Lhx2	transcription factor	nuclear	NM_010710
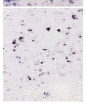	Lrmp	unknown function	integral membrane protein	NM_008511

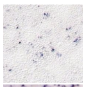	Ncf1	subunit of neutrophil NADPH oxidase	cytoplasm	NM_010876
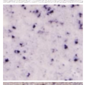	Parp8	ADP-ribose polymerization	cytoplasm	NM_027272
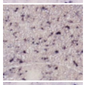	Psap	saposin precursor	secreted and integral membrane protein	NM_011179
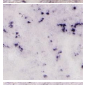	Serpinb1b	peptidase inhibitor activity	cytoplasm	NM_173052
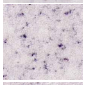	Sfpi1	transcription factor	nuclear	NM_011355
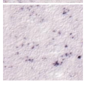	Syne1	actin binding	nuclear envelope	XM_358299

Group4: clustered cells, d ≈ cd

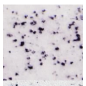	Emb	cell adhesion	integral membrane protein	NM_010330
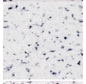	Emr1	fatty acid biosynthetic process	integral to membrane of ER	NM_010130
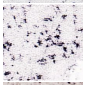	Hmox1	heme oxygenase activity	ER	NM_010442
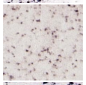	**Kit** (CD117)	protein tyrosine kinase activity	integral membrane protein	NM_021099
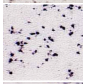	Mpo	heme binding	mitochondrial	NM_010824
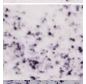	Myb	transcription factor	nuclear	NM_010848
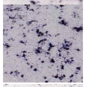	Plac8	immune response	unknown	NM_139198
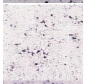	**Ptprc** (CD45R)	protein tyrosine phosphatase activity	integral membrane protein	NM_011210
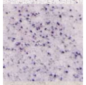	Slc17a3	transporter	integral membrane protein	NM_134069
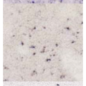	Spic	transcription factor	nuclear	NM_011461
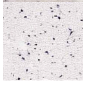	Stab2	cell adhesion	integral membrane protein	NM_138673

Abbreviations: d, distance between cells; cd, cell diameter; ER, endoplasmatic reticulum.
