# Supplementary material for: A High-Resolution Anatomical Atlas of the Transcriptome in the Mouse Embryo
Source: PLoS Biol. 2011 Jan 18;9(1):e1000582. doi: 10.1371/journal.pbio.1000582 (PMC3022534; doi:10.1371/journal.pbio.1000582)
Supplement: Text S1 — Supporting methods. This file gives an overview of the methods used in this manuscript. Additional supplementary data on clustering can be found at http://www.eurexpress.org/ee. (0.20 MB DOC) [file pbio.1000582.s018.docx]

**Supporting Methods**

**Embryo Staging**

Standard operating protocols for embryo harvesting were established in order to reduce variation (available at www.eurexpress.org). In addition, embryo staging was assessed by counting somites in assays that show expression in axial skeleton (that makes it possible to count with confidence). We focused this analysis on the somites caudal to the first 30 vertebrae. Our analysis revealed that the majority of the embryos (70%) analysed displayed between 13 and 15 somites which is highly consistent with the E14.5 stage. In addition, we looked at other anatomical structures described in the Kauffman atlas that show defined characteristics at different developmental stages such as: interdigital spaces and nail plates.

## Web and Internet Applications

The TDB and Gene Expression Data Repository (GEDR) were at the center of the EURExpress transcriptome atlas. The GEDR was implemented using the MySQL database management system and disk arrays to store the section images in various formats and sizes. The MySQL database receives textual data, which is generated as a result of processing of "raw" template, sectioning, hybridization and assay data. A number of Perl, Javascript and Shell scripts were deployed to manage and process the enormous section image data set and associated textual information. These scripts move, sort, resize and assemble the section images in order to generate assays and also to produce several versions of each image, which are available for different purposes.

The data in GEDR is accessible through the EURExpress website. This is served by a combination of Apache and Tomcat web servers. The Apache web server receives requests from users via a web browser and either handles the request directly or it passes it on to the Tomcat web server. Apache processes the requests for static content and PHP based applications (e.g. the zoom viewer). For JSP/Java Servlet-based content it proxies the request to the Tomcat web server. To enhance user’s experience and speed-up the service the website makes use of AJAX technology.

To develop the web-based applications and manage the data, the project makes use of Linux-based machines. The main tools for web development include Eclipse (Integrated Development Environment), Exadel (for JSP coding), CVS (for version control) and Ant (for automating build). All the tools and technologies used for information management by the EURExpress project are freely available and most of these are open-source.

For redundancy and reliability the entire EURExpress system is backed-up on a daily basis. The system is deployed on dual-cpu, and dual-core opteron-based linux (Mandrake 10.5) servers linked to a 25TB SAN for primary store.

**Data browsing and viewing**

The data on the EURExpress website can be searched by keyword, by sequence and by anatomy. Once the user selects a particular gene or genes an interface provides a window with a bar chart that shows summary statistics representing the strength and coverage of expression within a set of anatomical structure st groups. This statistic is defined on the web-site and is designed to capture aspects of the strength and distribution of the expression within each group. The expression strengths of ‘strong’, ‘moderate’, ‘weak’ and ‘possible’ are converted to numerical values 10, 5, 2 and 1, respectively, and a weighted average of strength is calculated then expressed as a percentage. The coverage statistic is the proportion of the terms of a structure group that are annotated (or have a parent term annotated) The precise definitions of the calculations of strength and coverage are accessible from the “?” symbol next to the bar-chart.

The bar charts provide an overview of the pattern of expression. For individual structures, the strength of expression is indicated in the anatomy tree by text colour. Since the anatomy tree shows the actual annotation provided by the editors, the coverage is explicit and readily understood. It would be possible to propagate a coverage value “up the tree” using the formula already established, but we feel this would be an unnecessarily burden on the interface since the tree can easily be expanded to allow users to assess the overall coverage for themselves.

The bar-charts provide a very efficient read-out of the overall pattern and when coupled with the query options can be used to find similar patterns across two of the structure groups. In addition for each assay we provide two more pattern similarity options. The first is provided by the “Similar Assays” tab for which assays are compared pair-wise. The second is the provision of a syn-expression link for assays that have been assigned a cluster group in the full clustering of the data.

EURExpress Anatomy Atlas.

The user can select to use the “EURExpress Anatomy Atlas” as a reference guide when viewing expression patterns. The atlas can be accessed by clicking on the main menu “Tools and Analysis” tab and selecting “Anatomy Atlas” <http://arran.hgu.mrc.ac.uk/eAtlasViewer/php/eurexpress>AnatomyAtlas.php.

This opens up a new browser window that shows the image of an embryo with the labeled anatomical structures. The images have a very simple overlay option with controllable transparency. It is up to the user to select the appropriate view from the 8 standard views that are available.

**Image annotation**

The process for image annotation is as follows:

1. *Check image and in situ pattern quality.*
2. *Examine all areas of the image for evidence of gene expression.*
3. *For each expression region select the anatomical tissues that show expression in the anatomy list (multi-select is permitted).*
4. *Select the relative strength of expression, pattern type and add comments if required.*
5. *Continue for all other regions of expression in the image.*
6. *Select the next image and repeat from 1.*
7. *When all images are complete, assign status “complete” to the assay.*
8. *All “complete” assays must be re-reviewed and status set to “group agreed”.*
9. *“Group agreed” assays are then signed-off and the annotation is automatically sent to the TDB and the website updated.*

FIATAS was designed to require completion of each stage before the status of the annotation is effectively changed.

**Image processing**

The colored images represented in Figures 6-7 and Figure S6 were generated by the alignment and superposition of sections in which a single-selected color was assigned using grey scale mode transformation of the original ISH images. These were then inversed, copied and “channel-specific pasted” using the tools available in the Photoshop program. Then, different colored layers were aligned following clear and stable anatomic landmarks, and finally combined into a single layer.

Then, all the anatomical reference points were used as a framework to superimpose equivalent sections from different assays to get the “new image” of pseudo-colored expression patterns. We used the free rotation tool in Photoshop to align the small differences in image rotation.

For Figure 6, the thalamus, for example, the following key components of the developing CNS anatomy were used as reference:

1) the retroflexus tract extension, visisble in most of its extension in one section at each side of the embryo (two images in each assay), including its origin in the epithlamus (rf and ET). Rf is a narrow axonal tract crossing the caudal thalamus from dorsal to ventral);

2) the similar level of medial pallium section (MP);

3) the presence of the mammillotegmental tract in the prethalamic and thalamic tegmentum (PThTg and TTg, respectively; this tract is longitudinal along the diencephalic basal plate),

4) the stria medullaris tract in the dorsal pole of prethalamic eminence (EPth);

The presence of these structures in all sections, which were compared, allowed identifying these sections as belonging to the same parasagittal level. Indeed, only one section (at each side of the embryo) shows all these structures and is comparable among assays.

Shape and size of ganglionic eminences, choroidal plexus insertion point in the MP and EPTh, and the previously described diencephalic landmarks were carefully observed to develop the cerebral cortex comparative composition (Figure S6).

In addition to the previous described landmarks, the insertion of choroidal tela in the rhombic lip and the cerebellum, as well as the first cervical vertebral bodies, were used as landmarks to align sections to develop the spinal cord composition (Figure 6).

## Data Management

Data management was based on interactions with a central tracking database (TDB). The link between the central database and each activity was managed via a combination of web-services and ftp with data exchanged either in excel, XML or jpeg formats. The architecture is shown in Figure S7. The TDB was implemented using mySQL on a linux server and the website and web-applications using an Apache/Tomcat server with JSP and Javascript/Ajax technologies. Details of the database implementation, schema and web-applications are available on request.
